# Supplementary material for: Health service utilization and associated factors among fee waiver beneficiaries in Ethiopia: Systematic review and meta-analysis
Source: PLoS One. 2025 Jun 11;20(6):e0326131. doi: 10.1371/journal.pone.0326131 (PMC12157077; doi:10.1371/journal.pone.0326131)
Supplement: Supplemental File 1 — (DOCX) [file pone.0326131.s001.docx]

# All databases search strategy detail for systematic review and meta-analysis to estimate the prevalence of health service utilization and associated factors among fee waiver beneficiaries in Ethiopia, 2024

# PubMed searching strategy results (#187)

| **Search number** | **Search query** | **Results** |
| --- | --- | --- |
|  | "Health Services"[MeSH Terms] | 2,537,086 |
|  | (((((((((Health Service[Title/Abstract]) OR (Services, Health[Title/Abstract])) OR (Health care[Title/Abstract])) OR (medical service*[Title/Abstract])) OR (healthcare service*[Title/Abstract])) OR (clinical service*[Title/Abstract])) OR ("Comprehensive Health Care"[MeSH Terms])) OR (Healthcare, Comprehensive[Title/Abstract])) OR (Comprehensive Healthcare[Title/Abstract])) OR (Healthcare, Comprehensive[Title/Abstract]) | [892,277](https://pubmed.ncbi.nlm.nih.gov/?term=%28%28%28%28%28%28%28%28%28Health+Service%5BTitle%2FAbstract%5D%29+OR+%28Services%2C+Health%5BTitle%2FAbstract%5D%29%29+OR+%28Health+care%5BTitle%2FAbstract%5D%29%29+OR+%28medical+service%2A%5BTitle%2FAbstract%5D%29%29+OR+%28healthcare+service%2A%5BTitle%2FAbstract%5D%29%29+OR+%28clinical+service%2A%5BTitle%2FAbstract%5D%29%29+OR+%28%22Comprehensive+Health+Care%22%5BMeSH+Terms%5D%29%29+OR+%28Healthcare%2C+Comprehensive%5BTitle%2FAbstract%5D%29%29+OR+%28Comprehensive+Healthcare%5BTitle%2FAbstract%5D%29%29+OR+%28Healthcare%2C+Comprehensive%5BTitle%2FAbstract%5D%29&ac=no&sort=relevance) |
|  | "statistics and numerical data"[MeSH Subheading] | [3,535,373](https://pubmed.ncbi.nlm.nih.gov/?term=%22statistics++and++numerical+data%22%5BMeSH+Subheading%5D&ac=no&sort=relevance) |
|  | ((((((statistics and numerical data[Title/Abstract]) OR (utili*ation[Title/Abstract])) OR (use[Title/Abstract])) OR (numerical data[Title/Abstract])) OR (Uptake[Title/Abstract])) OR (usage[Title/Abstract])) OR (consumption[Title/Abstract]) | [4,792,098](https://pubmed.ncbi.nlm.nih.gov/?term=%28%28%28%28%28%28statistics+and+numerical+data%5BTitle%2FAbstract%5D%29+OR+%28utili%2Aation%5BTitle%2FAbstract%5D%29%29+OR+%28use%5BTitle%2FAbstract%5D%29%29+OR+%28numerical+data%5BTitle%2FAbstract%5D%29%29+OR+%28Uptake%5BTitle%2FAbstract%5D%29%29+OR+%28usage%5BTitle%2FAbstract%5D%29%29+OR+%28consumption%5BTitle%2FAbstract%5D%29&ac=no&sort=relevance) |
|  | Poverty[MeSH Terms] | [51,752](https://pubmed.ncbi.nlm.nih.gov/?term=Poverty%5BMeSH+Terms%5D&ac=no&sort=relevance) |
|  | (((((((((((((((((((((((((((((((((((Indigents[Title/Abstract]) OR (Indigent[Title/Abstract])) OR (Low-Income Population[Title/Abstract])) OR (Low-Income Populations[Title/Abstract])) OR (Population, Low-Income[Title/Abstract])) OR (Low Income Population[Title/Abstract])) OR (Low Income Populations[Title/Abstract])) OR (Population, Low Income[Title/Abstract])) OR (Indigency[Title/Abstract])) OR (Extreme Poverty[Title/Abstract])) OR (Poverty, Extreme[Title/Abstract])) OR (Absolute Poverty[Title/Abstract])) OR (Poverty, Absolute[Title/Abstract])) OR (Federal Poverty Threshold[Title/Abstract])) OR (Poverty Threshold, Federal[Title/Abstract])) OR (Federal Poverty Level[Title/Abstract])) OR (Federal Poverty Levels[Title/Abstract])) OR (Level, Federal Poverty[Title/Abstract])) OR (Poverty Level, Federal[Title/Abstract])) OR ("Medically Uninsured"[MeSH Terms])) OR (Medically Underinsured[Title/Abstract])) OR (Medically Underinsured[Title/Abstract])) OR (Underinsured[Title/Abstract])) OR (Uninsured[Title/Abstract])) OR (Fee waiver recipient*[Title/Abstract])) OR (Fee waiver beneficiar*[Title/Abstract])) OR (Payment waiver beneficiar*[Title/Abstract])) OR (Fee exemption recipient*[Title/Abstract])) OR (Cost relief beneficiar*[Title/Abstract])) OR (Subsidy recipient*[Title/Abstract])) OR (Fee relief beneficiar*[Title/Abstract])) OR (Cost waiver recipient*[Title/Abstract])) OR (Financial aid beneficiar*[Title/Abstract])) OR (Fee discount recipient*[Title/Abstract])) OR ("Medical Assistance"[MeSH Terms])) OR (Assistance, Medical[Title/Abstract]) | [96,236](https://pubmed.ncbi.nlm.nih.gov/?term=%28%28%28%28%28%28%28%28%28%28%28%28%28%28%28%28%28%28%28%28%28%28%28%28%28%28%28%28%28%28%28%28%28%28%28Indigents%5BTitle%2FAbstract%5D%29+OR+%28Indigent%5BTitle%2FAbstract%5D%29%29+OR+%28Low-Income+Population%5BTitle%2FAbstract%5D%29%29+OR+%28Low-Income+Populations%5BTitle%2FAbstract%5D%29%29+OR+%28Population%2C+Low-Income%5BTitle%2FAbstract%5D%29%29+OR+%28Low+Income+Population%5BTitle%2FAbstract%5D%29%29+OR+%28Low+Income+Populations%5BTitle%2FAbstract%5D%29%29+OR+%28Population%2C+Low+Income%5BTitle%2FAbstract%5D%29%29+OR+%28Indigency%5BTitle%2FAbstract%5D%29%29+OR+%28Extreme+Poverty%5BTitle%2FAbstract%5D%29%29+OR+%28Poverty%2C+Extreme%5BTitle%2FAbstract%5D%29%29+OR+%28Absolute+Poverty%5BTitle%2FAbstract%5D%29%29+OR+%28Poverty%2C+Absolute%5BTitle%2FAbstract%5D%29%29+OR+%28Federal+Poverty+Threshold%5BTitle%2FAbstract%5D%29%29+OR+%28Poverty+Threshold%2C+Federal%5BTitle%2FAbstract%5D%29%29+OR+%28Federal+Poverty+Level%5BTitle%2FAbstract%5D%29%29+OR+%28Federal+Poverty+Levels%5BTitle%2FAbstract%5D%29%29+OR+%28Level%2C+Federal+Poverty%5BTitle%2FAbstract%5D%29%29+OR+%28Poverty+Level%2C+Federal%5BTitle%2FAbstract%5D%29%29+OR+%28%22Medically+Uninsured%22%5BMeSH+Terms%5D%29%29+OR+%28Medically+Underinsured%5BTitle%2FAbstract%5D%29%29+OR+%28Medically+Underinsured%5BTitle%2FAbstract%5D%29%29+OR+%28Underinsured%5BTitle%2FAbstract%5D%29%29+OR+%28Uninsured%5BTitle%2FAbstract%5D%29%29+OR+%28Fee+waiver+recipient%2A%5BTitle%2FAbstract%5D%29%29+OR+%28Fee+waiver+beneficiar%2A%5BTitle%2FAbstract%5D%29%29+OR+%28Payment+waiver+beneficiar%2A%5BTitle%2FAbstract%5D%29%29+OR+%28Fee+exemption+recipient%2A%5BTitle%2FAbstract%5D%29%29+OR+%28Cost+relief+beneficiar%2A%5BTitle%2FAbstract%5D%29%29+OR+%28Subsidy+recipient%2A%5BTitle%2FAbstract%5D%29%29+OR+%28Fee+relief+beneficiar%2A%5BTitle%2FAbstract%5D%29%29+OR+%28Cost+waiver+recipient%2A%5BTitle%2FAbstract%5D%29%29+OR+%28Financial+aid+beneficiar%2A%5BTitle%2FAbstract%5D%29%29+OR+%28Fee+discount+recipient%2A%5BTitle%2FAbstract%5D%29%29+OR+%28%22Medical+Assistance%22%5BMeSH+Terms%5D%29%29+OR+%252) |
|  | ("Ethiopia"[MeSH Terms]) OR (Federal Democratic Republic of Ethiopia[Title/Abstract]) | [22,721](https://pubmed.ncbi.nlm.nih.gov/?term=%28%22Ethiopia%22%5BMeSH+Terms%5D%29+OR+%28Federal+Democratic+Republic+of+Ethiopia%5BTitle%2FAbstract%5D%29&ac=no&sort=relevance) |
|  | #1 OR #2 AND #3 OR #4 AND #5 OR #6 AND #7 | 187 |

***Google scholar searching strategy results (#215)***

"Health service*" OR "Health care" OR "Medical care" OR "clinical service*" OR "Medical Service*" AND utilization OR uptake OR use OR usage OR consumption OR receipt AND "fee waiver" OR "fee exempt*" AND beneficiaries OR recipient* user* AND Ethiopia OR "federal democratic republic of Ethiopia"

***African Journals Online (AJOL) searching strategy results (#41)***

"Health service*" OR "Health care" OR "Medical care" OR "clinical service*" OR "Medical Service*" AND utilization OR uptake OR use OR usage OR consumption OR receipt AND "fee waiver" OR "fee exempt*" AND beneficiaries OR recipient* user* AND Ethiopia OR "federal democratic republic of Ethiopia"

***Research4life searching strategy results (#33)***

((Title/Abstract:("Health service*")) OR (Title/Abstract:("Health care")) OR (Title/Abstract:("Medical care")) OR (Title/Abstract:("clinical service*")) OR (Title/Abstract:("Medical Service*"))) AND (Title/Abstract:(utilization)) OR (Title/Abstract:(uptake)) OR (Title/Abstract:(use)) OR (Title/Abstract:(usage)) OR (Title/Abstract:(consumption)) OR (Title/Abstract:(receipt))) AND ((Title/Abstract:("fee waiver")) OR (Title/Abstract:("fee exempt*"))) AND ((Title/Abstract:(beneficiaries)) OR (Title/Abstract:(recipient*)) OR (Title/Abstract:(user*))) AND ((Title/Abstract:(Ethiopia)) OR (Title/Abstract:("federal democratic republic of Ethiopia")))

***Science direct searching strategy results; (#27)***

"Health service*" OR "Health care" OR "Medical care" OR "clinical service*" OR "Medical Service*" AND utilization OR uptake OR use OR usage OR consumption OR receipt AND "fee waiver" OR "fee exempt*" AND beneficiaries OR recipient* user* AND Ethiopia OR "federal democratic republic of Ethiopia"

***CINAHAL searching strategy results (#12)***

((TitleCombined:("Health service*")) OR (TitleCombined:("Health care")) OR (TitleCombined:("Medical care")) OR (TitleCombined:("clinical service*")) OR (TitleCombined:("Medical Service*"))) AND (TitleCombined:(utilization)) OR (TitleCombined:(uptake)) OR (TitleCombined:(use)) OR (TitleCombined:(usage)) OR (TitleCombined:(consumption)) OR (TitleCombined:(receipt))) AND ((TitleCombined:("fee waiver")) OR (TitleCombined:("fee exempt*"))) AND ((TitleCombined:(beneficiaries)) OR (TitleCombined:(recipient*)) OR (TitleCombined:(user*))) AND ((TitleCombined:(Ethiopia)) OR (TitleCombined:("federal democratic republic of Ethiopia")))
