# Supplementary material for: Health service utilization and associated factors among fee waiver beneficiaries in Ethiopia: Systematic review and meta-analysis
Source: PLoS One. 2025 Jun 11;20(6):e0326131. doi: 10.1371/journal.pone.0326131 (PMC12157077; doi:10.1371/journal.pone.0326131)
Supplement: Supplemental File 2 — (DOCX) [file pone.0326131.s002.docx]

# List of excluded studies from the systematic review and meta-analysis of health service utilization and associated factors among fee waiver beneficiaries in Ethiopia, 2024

| Author | Publication year | Title | Reason for exclusion |
| --- | --- | --- | --- |
| Abate, G. et al[[1](#_ENREF_1)] | 2000 | Health seeking and hygiene behaviours predict nutritional status of pre-school children in a slum area of Addis Ababa, Ethiopia | Excluded by title |
| Abayneh, S. et al[[2](#_ENREF_2)] | 2017 | Service user involvement in mental health system strengthening in a rural African setting: qualitative study | Excluded by title |
| Abdallah, W. et al[[3](#_ENREF_3)] | 2022 | Access and fees in public health care services for the poor: Bangladesh as a case study | Excluded by title |
| Abdela, S. G. et al[[4](#_ENREF_4)] | 2020 | Looking for NTDs in the skin; an entry door for offering patient centered holistic care | Excluded by title |
| Abdou Illou, M. M. et al[[5](#_ENREF_5)] | 2015 | The elimination of healthcare user fees for children under five substantially alleviates the burden on household expenses in Burkina Faso | Excluded by title |
| Abebaw, F. et al[[6](#_ENREF_6)] | 2019 | Catastrophic health expenditure and impoverishment in households of persons with depression: a cross-sectional, comparative study in rural Ethiopia | Excluded by title |
| Abessa, T. G. et al[[7](#_ENREF_7)] | 2016 | Adaptation and standardization of a Western tool for assessing child development in non-Western low-income context | Excluded by title |
| Abel-Smith, B. & Rawal, P.[[8](#_ENREF_8)] | 1992 | Can the poor afford ‘free’health services? A case study of Tanzania | Excluded by title |
| Abebe A.[[9](#_ENREF_9)] | 2014 | The Study on Effectiveness of Community Based Micro Health Insurance Scheme attached with Women Self Help GROUP (SHG) approach in Ethiopia: the Case of Jimma Town Women SHGs," St. Mary's University | Excluded by title |
| Abebe Z[[10](#_ENREF_10)] | 2016 | The Contribution of Community Based Health Insurance (CBHI) in Improving Access and Utilization of Healthcare Services: The Case of Adea District, East Shoa Zone, Oromia Region, Ethiopia," St. Mary’s University. | Excluded by title |
| Abelson, J. & Lomas, J.[[11](#_ENREF_11)] | 1990 | Do health service organizations and community health centres have higher disease prevention and health promotion levels than fee-for-service practices? | Excluded by title |
| Abrahim, O. et al[[12](#_ENREF_12)] | 2015 | A Patient-Centered Understanding of the Referral System in Ethiopian Primary Health Care Units | Excluded by title |
| Abu, D. et al[[13](#_ENREF_13)] | 2022 | Willingness to take COVID-19 vaccination in low-income countries: Evidence from Ethiopia | Excluded by title |
| Accorsi, S. et al[[14](#_ENREF_14)] | 2009 | Skin disorders and disease profile of poverty: analysis of medical records in Tigray, northern Ethiopia, 2005-2007 | Excluded by title |
| Accorsi, S. et al[[15](#_ENREF_15)] | 2009 | Poverty, inequality and health: the challenge of the double burden of disease in a non-profit hospital in rural Ethiopia | Excluded by abstract |
| Adabre A. & Chan P.[[16](#_ENREF_16)] | 2019 | Critical success factors (CSFs) for sustainable affordable housing," Building and Environment, | Excluded by title |
| Adane, K. et al[[17](#_ENREF_17)] | 2015 | The revenue generated from clinical chemistry and hematology laboratory services as determined using activity-based costing (ABC) model," Cost Effectiveness and Resource Allocation, vol. 13, pp. 1-7, | Excluded by title |
| Adane, M. et al[[18](#_ENREF_18)] | 2017 | Piped water supply interruptions and acute diarrhea among under-five children in Addis Ababa slums, Ethiopia: A matched case-control study | Excluded by title |
| Adane, M. et al[[19](#_ENREF_19)] | 2017 | Utilization of health facilities and predictors of health-seeking behavior for under-five children with acute diarrhea in slums of Addis Ababa, Ethiopia: a community-based cross-sectional study | Excluded by abstract |
| Adish, A. A. et al[[20](#_ENREF_20)] | 1999 | Risk factors for iron deficiency anaemia in preschool children in northern Ethiopia | Excluded by title |
| Admassu, T. W. et al[[21](#_ENREF_21)] | 2022 | Ethiopia has a long way to go meeting adolescent and youth sexual reproductive health needs | Excluded by title |
| Afkhar, R. [[22](#_ENREF_22)] | 2016 | Public Services, Social Protection, and Poverty," Universitäts-und Landesbibliothek Bonn | Excluded by title |
| McIntyre, D. et al | 2018 | Challenges in Financing Universal Health Coverage in Sub-Saharan Africa | Excluded by abstract |
| Agegnehu, A. & Behaylu, A.[[23](#_ENREF_23)] | 2015 | Health and Decentralization: The Case of Gozamin District, Amhara State, Ethiopia | Excluded by title |
| Agyepong, I. A.[[24](#_ENREF_24)] | 1999 | Reforming health service delivery at district level in Ghana: the perspective of a district medical officer | Excluded by title |
| Ahmed, S. et al[[25](#_ENREF_25)] | 2022 | Community health workers and health equity in low-and middle-income countries: systematic review and recommendations for policy and practice | Excluded by title |
| Ahmed, S. et al[[26](#_ENREF_26)] | 2010 | Economic status, education and empowerment: implications for maternal health service utilization in developing countries | Excluded by title |
| Ajayi, A.I[[27](#_ENREF_27)] | 2019 | I am alive; my baby is alive”: understanding reasons for satisfaction and dissatisfaction with maternal health care services in the context of user fee removal policy in Nigeria | Excluded by title |
| Ajayi, A. I. & Akpan, W. [[28](#_ENREF_28)] | 2017 | Who benefits from free institutional delivery? evidence from a cross-sectional survey of North Central and Southwestern Nigeria | Excluded by title |
| Alebachew, A. et al[[29](#_ENREF_29)] | 2017 | Ethiopia’s Progress in health financing and the contribution of the 1998 health care and financing strategy in Ethiopia | Excluded by abstract |
| Alem, A.[[30](#_ENREF_30)] | 2001 | Mental health services and epidemiology of mental health problems in Ethiopia | Excluded by title |
| Alemu, Y. M. et al[[31](#_ENREF_31)] | 2017 | Utilization of HIV testing services among pregnant mothers in low income primary care settings in northern Ethiopia: a cross sectional study | Excluded by abstract |
| Alene, G. D. & Worku, A.[[32](#_ENREF_32)] | 2009 | Examining perceptions of rapid population growth in North and South Gondar zones, northwest Ethiopia | Excluded by title |
| Alene, M. et al[[33](#_ENREF_33)] | 2019 | Health care utilization for common childhood illnesses in rural parts of Ethiopia: evidence from the 2016 Ethiopian demographic and health survey, | Excluded by abstract |
| Ali, E. E[[34](#_ENREF_34)] | 2014 | Health care financing in Ethiopia: implications on access to essential medicines | Excluded by abstract |
| Ali, E. E, et al[[35](#_ENREF_35)] | 2014 | Pharmaceutical pricing in Ethiopia | Excluded by title |
| Amarech, G. O. & John, E. A.[[36](#_ENREF_36)] | 2020 | Assessing medical impoverishment and associated factors in health care in Ethiopia | Excluded by abstract |
| Amexo, M. et al[[37](#_ENREF_37)] | 2004 | Malaria misdiagnosis: effects on the poor and vulnerable | Excluded by title |
| Ameya, G. et al[[38](#_ENREF_38)] | 2018 | Antimicrobial susceptibility pattern, and associated factors of Salmonella and Shigella infections among under five children in Arba Minch, South Ethiopia | Excluded by title |
| Amporfu, E.[[39](#_ENREF_39)] | 2013 | Effect of regulated user fee on quality of healthcare for the poor and the non-poor | Excluded by title |
| Amporfu, E.[[40](#_ENREF_40)] | 2014 | A theoretical analysis of how user fee on healthcare can waste economic resources | Excluded by title |
| Anangwe, A.[[41](#_ENREF_41)] | 2008 | Health sector reforms in Kenya: User fees | Excluded by title |
| Anderson, J. R[[42](#_ENREF_42)] | 2003 | Rural extension services | Excluded by title |
| Anderson, G. F.[[43](#_ENREF_43)] | 2007 | From ‘soak the rich’to ‘soak the poor’: recent trends in hospital pricing | Excluded by title |
| Ecorys, A. et al[[44](#_ENREF_44)] | 2020 | Mitigating the socio-economic impacts of COVID-19 in Ethiopia, with a focus on vulnerable groups | Excluded by title |
| Argaw, M. D. et al[[45](#_ENREF_45)] | 2020 | Accelerating the performance of district health systems towards achieving UHC via twinning partnerships | Excluded by title |
| Arhin-Tenkorang, D.[[46](#_ENREF_46)] | 2001 | Mobilizing resources for health: the case for user fees revisited | Excluded by title |
| Arsenault, C. et al[[47](#_ENREF_47)] | 2021 | Using health management information system data: case study and verification of institutional deliveries in Ethiopia | Excluded by title |
| Aserese, A. D. et al[[48](#_ENREF_48)] | 2021 | Adequate vitamin A rich food consumption and associated factors among lactating mothers visiting child immunization and post-natal clinic at health institutions in Gondar Town, Northwest Ethiopia | Excluded by title |
| Asher, L. et al[[49](#_ENREF_49)] | 2018 | Community-based rehabilitation intervention for people with schizophrenia in Ethiopia (RISE): a 12 month mixed methods pilot study | Excluded by title |
| Asker, I. M. & Nuh, A. M.[[50](#_ENREF_50)] | 2024 | Utilization of health service and associated factors among women in childbearing age in selected internally displaced persons camps, Hargeisa–Somaliland | Excluded by title |
| Assefa, E. M.[[51](#_ENREF_51)] | 2019 | Knowledge, attitude and practice (KAP) of health providers towards safe abortion provision in Addis Ababa health centers | Excluded by title |
| Assefa, N. et al[[52](#_ENREF_52)] | 2012 | The hazard of pregnancy loss and stillbirth among women in Kersa, East Ethiopia: a follow up study | Excluded by title |
| Atakoğlu Y. R. et al[[53](#_ENREF_53)] | 2023 | Challenges and Affecting Factors for Nurses in the Integration of Home Health Service and Palliative Care | Excluded by title |
| Atchessi, N. et al[[54](#_ENREF_54)]E | 2016 | User fees exemptions alone are not enough to increase indigent use of healthcare services | Excluded by abstract |
| Atinga, R. A. et al[[55](#_ENREF_55)] | 2012 | Migrating from user fees to social health insurance: exploring the prospects and challenges for hospital management | Excluded by title |
| Atun, R. et al.[[56](#_ENREF_56)] | 2016 | Poverty alleviation and the economic benefits of investing in health | Excluded by abstract |
| Avan, B. I. et al[[57](#_ENREF_57)] | 2016 | District decision-making for health in low-income settings: a feasibility study of a data-informed platform for health in India, Nigeria and Ethiopia | Excluded by title |
| Ayele, D. G. et al[[58](#_ENREF_58)] | 2014 | Semiparametric models for malaria rapid diagnosis test result | Excluded by title |
| Azag, M. & Haile, D.[[59](#_ENREF_59)] | 2015 | Factors affecting healthcare service utilization of mothers who had children with diarrhea in Ethiopia: evidence from a population based national survey | Excluded by abstract |
| Badasu, D. M.[[60](#_ENREF_60)] | 2004 | Implementation of Ghana’s health user fee policy and the exemption of the poor: problems and prospects | Excluded by title |
| Baird, S. et al[[61](#_ENREF_61)] | 2020 | Improving the use of focus group discussions in low income settings | Excluded by title |
| Balabanova, D. et al[[62](#_ENREF_62)] | 2004 | Health service utilization in the former Soviet Union: evidence from eight countries | Excluded by title |
| Barnett, I. & Tefera, B.[[63](#_ENREF_63)] | 2010 | Poor Households’ Experiences and Perception of User Fees for Healthcare: a mixed-method study from Ethiopia | Excluded by title |
| Baron, E. C. et al[[64](#_ENREF_64)] | 2016 | Maternal mental health in primary care in five low- and middle-income countries: a situational analysis | Excluded by title |
| Baron, E. C. et al[[65](#_ENREF_65)] | 2018 | Impact of district mental health care plans on symptom severity and functioning of patients with priority mental health conditions: the Programme for Improving Mental Health Care (PRIME) cohort protocol, | Excluded by title |
| Barrientos, A. and Smith, R.[[66](#_ENREF_66)] | 2005 | Social Assistance in Low Income Countries Database | Excluded by title |
| Baru, R. et al[[67](#_ENREF_67)] | 2010 | Inequities in access to health services in India: caste, class and region | Excluded by title |
| Barua, P. & Narattharaksa, K.[[68](#_ENREF_68)] | 2019 | Investigation into health disparities among stateless children in Tak Province, Thailand | Excluded by title |
| Basaza, R. K. et al[[69](#_ENREF_69)] | 2010 | Community health insurance amidst abolition of user fees in Uganda: the view from policy makers and health service managers | Excluded by title |
| Baschieri, A. & Hobson, J.[[70](#_ENREF_70)] | 2013 | Support for the Ethiopian Health Sector Development Programme (Federal Ministry of Health MDG Performance Fund) | Excluded by title |
| Baye, K.[[71](#_ENREF_71)] | 2019 | Prioritizing the Scale-Up of Evidence-Based Nutrition and Health Interventions to Accelerate Stunting Reduction in Ethiopia | Excluded by title |
| Bayked, E. M. et al[[72](#_ENREF_72)] | 2024 | Beneficiaries’ satisfaction with community-based health insurance services and associated factors in Ethiopia: a systematic review and meta-analysis | Excluded by title |
| Bayou, Y. T. et al[[73](#_ENREF_73)] | 2016 | The adequacy of antenatal care services among slum residents in Addis Ababa, Ethiopia | Excluded by abstract |
| Beaugé, Y.[[74](#_ENREF_74)] | 2022 | Targeted User Fee Exemption for Equitable Access to Primary Healthcare Services for the Ultra-Poor: A multi-method study using the case of Burkina Faso," 2022. | Excluded by title |
| Bedaso, A. et al[[75](#_ENREF_75)] | 2020 | Quality of sleep and associated factors among people living with HIV/AIDS attending ART clinic at Hawassa University comprehensive specialized Hospital, Hawassa, SNNPR, Ethiopia | Excluded by title |
| Bedford, J.[[76](#_ENREF_76)] | 2012 | Qualitative research to identify solutions to local barriers to care-seeking and treatment for diarrhoea, malaria and pneumonia in select high burden countries | Excluded by title |
| Bekele, M. et al[[77](#_ENREF_77)] | 2024 | Farm forests, seasonal hunger, and biomass poverty: Evidence of induced intensification from panel data in the Ethiopian Highlands | Excluded by title |
| Belay, M. & Deressa, W.[[78](#_ENREF_78)] | 2008 | Use of insecticide treated nets by pregnant women and associated factors in a pre-dominantly rural population in northern Ethiopia | Excluded by title |
| Belew, M. et al[[79](#_ENREF_79)] | 2000 | The magnitude of khat use and its association with health, nutrition and socio-economic status | Excluded by title |
| Belete, G. T. & Walle, Y.[[76](#_ENREF_76)] | 2023 | Willingness to pay for medical care and its determinants in private health care facilities among Gondar city residents, Northwest Ethiopia: Cross sectional study | Excluded by abstract |
| Berhan, Y.[[80](#_ENREF_80)] | 2014 | Predictors of perinatal mortality associated with placenta previa and placental abruption: an experience from a low income country | Excluded by title |
| Berhane D. F. & Danis, M.[[81](#_ENREF_81)] | 2019 | Bedside Rationing Under Resource Constraints-A National Survey of Ethiopian Physicians' Use of Criteria for Priority Setting | Excluded by title |
| Berhane, Y. et al[[82](#_ENREF_82)] | 2001 | Women's health in a rural setting in societal transition in Ethiopia | Excluded by abstract |
| Berhan, T. et al[[83](#_ENREF_83)] | 2024 | Catastrophic out-of-pocket payments related to non-communicable disease multimorbidity and associated factors, evidence from a public referral hospital in Addis Ababa Ethiopia | Excluded by title |
| Berk, M. & McGivern, L. M.[[84](#_ENREF_84)] | 2016 | Effects of a Facilitated Fee Waiver Program on Participation in Youth Sports Programs | Excluded by title |
| Besley, T. & Coate, S.[[85](#_ENREF_85)] | 1991 | Public provision of private goods and the redistribution of income | Excluded by title |
| Beyene, A. et al[[86](#_ENREF_86)] | 2015 | Current state and trends of access to sanitation in Ethiopia and the need to revise indicators to monitor progress in the Post-2015 era | Excluded by title |
| Bhattacharyya, S. et al[[87](#_ENREF_87)] | 2016 | District decision-making for health in low-income settings: a case study of the potential of public and private sector data in India and Ethiopia | Excluded by title |
| Biliguo, S.[[88](#_ENREF_88)] | 2020 | Access to complementary services of likelihood empowerment against poverty-(leap) social protection intervention in Nadowli-Kaleo district, Ghana | Excluded by title |
| Birara, M. et al[[89](#_ENREF_89)] | 2024 | Locally advanced cervical cancer: Neoadjuvant chemotherapy plus radical surgery an alternative approach to chemo-radiation in a low-income setting | Excluded by title |
| Bitran, R. & Martorell, B.[[90](#_ENREF_90)] | 2009 | AMFm: Reaching the poorest of the poor with effective malaria drugs | Excluded by abstract |
| Bitrán, R. & Giedion, U.[[91](#_ENREF_91)] | 2002 | Waivers and exemptions for health services in developing countries. Final draft | Excluded by title |
| Bivins, R. et al[[92](#_ENREF_92)] | 2022 | Posters, protests, and prescriptions : Cultural histories of the National Health Service in Britain | Excluded by title |
| Bogale, G. W. et al[[93](#_ENREF_93)] | 2010 | Reaching the hearts and minds of illiterate women in the Amhara highland of Ethiopia: Development and pre-testing of oral HIV/AIDS prevention message | Excluded by title |
| Bogetic, Z. el al[[94](#_ENREF_94)] | 2015 | How Does Knowledge on Public Expenditures Integrate with the Design of Development Policy Operations? | Excluded by title |
| Borde, M. T.[[95](#_ENREF_95)] | 2023 | Geographical and Socioeconomic Inequalities in Maternal Mortality in Ethiopia | Excluded by abstract |
| Borowy, I.[[96](#_ENREF_96)] | 2016 | Medical Aid, Repression, and International Relations: The East German Hospital at Metema | Excluded by title |
| Boyce, N. W. et al[[97](#_ENREF_97)] | 1997 | Health Service Outcomes, Quality and outcome indicators for acute healthcare services: A research project for the National Hospital Outcomes Program (NHOP) Health Service Outcomes Branch | Excluded by title |
| Brikci, N.[[98](#_ENREF_98)] | 2007 | The Failure of User Fees as a Health Financing Mechanism: An International Perspective | Excluded by title |
| Brikci, N. & Philips, M.[[99](#_ENREF_99)] | 2007 | User fees or equity funds in low-income countries | Excluded by title |
| Brink, A. S. & Koch, S. F.[[100](#_ENREF_100)] | 2015 | Did primary healthcare user fee abolition matter? Reconsidering South Africa's experience | Excluded by title |
| Burns, B. J. et al[[101](#_ENREF_101)] | 1995 | Children's mental health service use across service sectors | Excluded by title |
| Cafod, C. M.[[102](#_ENREF_102)] | 2005 | The cost of meeting the MDGs in Zambia | Excluded by title |
| Calhoun, L. M.et al[[103](#_ENREF_103)] | 2018 | The effect of the removal of user fees for delivery at public health facilities on institutional delivery in urban Kenya | Outside Ethiopia |
| Cambanis, A. et al[[104](#_ENREF_104)] | 2005 | Rural poverty and delayed presentation to tuberculosis services in Ethiopia | Excluded by abstract |
| Carr, D.[[105](#_ENREF_105)] | 2004 | Improving the Health | Excluded by title |
| Castro-Leal, F. et al[[106](#_ENREF_106)] | 2000 | Public spending on health care in Africa: do the poor benefit? | Excluded by title |
| Chali, J. C. et al[[107](#_ENREF_107)] | 2024 | Healthcare Paradigms | Excluded by abstract |
| Chapman, K.[[108](#_ENREF_108)] | 2006 | Using social transfers to scale up equitable access to education and health services | Excluded by abstract |
| Chapman, J. & Brown, A.[[109](#_ENREF_109)] | 2005 | Maternity units to the rescue | Excluded by title |
| Chauke, M. E. et al[[110](#_ENREF_110)] | 2024 | Trends of socioeconomic and geographic inequalities in severe wasting among under-five children in Ethiopia from 2000 to 2019: using the WHO Health Equity Assessment Toolkit | Excluded by title |
| Chauke, M. E. et al[[111](#_ENREF_111)] | 2018 | The effect of community based health insurance on catastrophic health expenditure in Northeast Ethiopia: A cross sectional study | Excluded by title |
| Chernet, M.[[112](#_ENREF_112)] | 2022 | Exploring The Facilitators of Community-Based Health Insurance Scheme Bankruptcy and its Magnitude in North Mecha District, West Gojjam Zone, Amhara, Ethiopia, | Excluded by title |
| Chersich, M. et al[[113](#_ENREF_113)] | 2016 | Safeguarding maternal and child health in South Africa by starting the Child Support Grant before birth: Design lessons from pregnancy support programmes in 27 countries | Excluded by title |
| Chikova, A.[[114](#_ENREF_114)] | 2020 | An analysis of the effects of the political environment on the governance of orphans and vulnerable children by non-state actors in Mutare, Zimbabwe | Excluded by title |
| Chirisa, I. [[115](#_ENREF_115)] | 2013 | Social protection amid increasing instability in Zimbabwe: Scope, institutions and policy options | Excluded by title |
| Chikova, A.[[114](#_ENREF_114)] | 2020 | An analysis of the effects of the political environment on the governance of orphans and vulnerable children by non-state actors in Mutare, Zimbabwe | Excluded by title |
| Chukwu, N.[[116](#_ENREF_116)] | 2024 | A blockchain-based framework and process guide for intelligent exchange and use of health information in low resource environments | Excluded by title |
| Chuma, J.et al[[117](#_ENREF_117)] | 2009 | Reducing user fees for primary health care in Kenya: Policy on paper or policy in practice? | Excluded by title |
| Cielo, B. et al[[118](#_ENREF_118)] | 2024 | Effect of a case-capped, fee-for-service payment mechanism on accessibility and affordability of health care | Excluded by title |
| Coady, D. et al[[119](#_ENREF_119)] | 2012 | Health Financing Systems in East Asia and the Pacific: Early Successes and Current Challenges | Excluded by title |
| Coady, D.[[120](#_ENREF_120)] | 2002 | The targeting of transfers in developing countries: Review of experience and lessons | Excluded by title |
| Çolakoğlu, S.[[121](#_ENREF_121)] | 2020 | Turkey’s COVID-19 Diplomacy: International cooperation in the age of global health and economic crisis | Excluded by title |
| Colantoni, A. et al[[122](#_ENREF_122)] | 2023 | Environmental enteropathy and its association with water sanitation and hygiene in slum areas of Jimma Town Ethiopia | Excluded by title |
| Congo, D. & Faso, B.[[123](#_ENREF_123)] | 2018 | Health Accounts Impacted Policy in over 30 Countries Worldwide | Excluded by title |
| Cook, J. A. et al[[124](#_ENREF_124)] | 2004 | A multi-site study of Medicaid-funded managed care versus fee-for-service plans' effects on mental health service utilization of children with severe emotional disturbance | Excluded by title |
| Coppock, D. L. et al[[125](#_ENREF_125)] | 2011 | Capacity building helps pastoral women transform impoverished communities in Ethiopia | Excluded by title |
| Cottin, R. | 2018 | Free Health Care for the Poor: a Good Way to Achieve Universal Health Coverage? | Excluded by abstract |
| Creese, A. L.[[126](#_ENREF_126)] | 1991 | User charges for health care: a review of recent experience | Excluded by title |
| Crystal, E. et al[[127](#_ENREF_127)] | 2004 | Equity Implications of Health Sector User Fees in Tanzania | Excluded by title |
| Cuellar F. I. et al[[128](#_ENREF_128)] | 2024 | Variability in the practice of ambulatory Clinical Psychology in the Health Service of Madrid: Variabilidad en la práctica de la Psicología Clínica ambulatoria | Excluded by title |
| Curry, L. et al[[129](#_ENREF_129)] | 2012 | Experiences of leadership in health care in sub-Saharan Africa | Excluded by title |
| Damerow, S.[[130](#_ENREF_130)] | 2023 | Maternal health in Sub-Saharan Africa: are national user fee waiver policies for intrapartum services the key to reducing maternal mortality? A quantitative cross-country comparison | Excluded by title |
| Damme, W. V. et al[[131](#_ENREF_131)] | 2004 | Out‐of‐pocket health expenditure and debt in poor households: evidence from Cambodia | Excluded by title |
| Damtew, S. A. & Shiferaw, S.[[132](#_ENREF_132)] | 2024 | Intimate Partner Violence during the Index Pregnancy and its correlates among a panel of pregnant women in Ethiopia, evidence from performance, and monitoring for action (PMA) 2021 cohort two baseline survey | Excluded by title |
| Dao, H. et al[[133](#_ENREF_133)] | 2008 | User fees and health service utilization in Vietnam: how to protect the poor? | Outside Ethiopia |
| Davidovich, E. et al[[134](#_ENREF_134)] | 2013 | Oral hygiene habits, dental home, and toothbrushing among immigrant and native low socioeconomic class populations | Excluded by title |
| Debebe, Z. Y. et al[[135](#_ENREF_135)] | 2014 | Impact of Ethiopia’s Community Based Health Insurance on household economic welfare | Excluded by title |
| Debie, A. et al[[136](#_ENREF_136)] | 2022 | Contributions and challenges of healthcare financing towards universal health coverage in Ethiopia: a narrative evidence synthesis, | Excluded by title |
| De Jong, J. T. et al[[137](#_ENREF_137)] | 2001 | Lifetime events and posttraumatic stress disorder in 4 postconflict settings | Excluded by title |
| Dean, C. M.[[138](#_ENREF_138)] | 2019 | A business health service: can businesses rely on professional specialist advice and assistance? | Excluded by title |
| Defar, A. et al[[139](#_ENREF_139)] | 2021 | Knowledge, practice and associated factors towards the prevention of COVID-19 among high-risk groups: A cross-sectional study in Addis Ababa, Ethiopia | Excluded by title |
| Degefa, M. B. et al[[140](#_ENREF_140)] | 2023 | Effect of community-based health insurance on catastrophic health expenditure among chronic disease patients in Asella referral hospital, Southeast Ethiopia: a comparative cross-sectional study | Excluded by title |
| De La H. W. & Alexis, S.[[141](#_ENREF_141)] | 2012 | The impact of a no-user-fee policy on the quality of patient care/service delivery in Jamaica | Excluded by title |
| Dean, C. M.[[138](#_ENREF_138)] | 2019 | A business health service: can businesses rely on professional specialist advice and assistance? | Excluded by title |
| Demelash, D.[[142](#_ENREF_142)] | 2021 | Willingness to Join and Pay for Social Health Insurance and Associated factors Among Civil Servants in Merawi Town, North West Ethiopia, | Excluded by title |
| Demissie, B. & Gutema N. K[[143](#_ENREF_143)] | 2020 | Effect of community-based health insurance on utilization of outpatient health care services in Southern Ethiopia: a comparative cross-sectional study | Excluded by abstract |
| Dennis, M. L.[[144](#_ENREF_144)] | 2020 | Pragmatic pluralism for health: Understanding the role of public financing and public-private engagement on use, quality, and equity in access to maternal health services in Kenya | Excluded by title |
| Dennis, M. L. et al[[145](#_ENREF_145)] | 2020 | Examining user fee reductions in public primary healthcare facilities in Kenya, 1997–2012: effects on the use and content of antenatal care | Excluded by title |
| Dercon, S. & Sánchez, A.[[146](#_ENREF_146)] | 2013 | Height in mid childhood and psychosocial competencies in late childhood: evidence from four developing countries | Excluded by title |
| Derseh, A.[[147](#_ENREF_147)] | 2013 | Enrolment in Ethiopia’s community based health insurance scheme | Excluded by title |
| Deyessa, N. et al[[148](#_ENREF_148)] | 2008 | Depression among women in rural Ethiopia as related to socioeconomic factors: a community-based study on women in reproductive age groups | Excluded by title |
| Dhufera, H. T.[[149](#_ENREF_149)] | 2024 | Identifying Indigent Households for Subsidy and Exemption of Community Health Insurance Premium in Ethiopia: Considerations of Technical Approaches and Implementation Challenges | Excluded by abstract |
| Diego, G.-C. et al[[150](#_ENREF_150)] | 2021 | Impact of COVID-19 prevention measures on health service quality, perceived value and user satisfaction. A structural equation modelling (SEM) approach | Excluded by title |
| Dingle, A.[[151](#_ENREF_151)] | 2016 | Equity of access to reproductive and maternal health services in Cambodia: equity trends, poverty targeting and demand-side financing," London School of Hygiene & Tropical Medicine | Excluded by title |
| Doka, B. K. et al[[152](#_ENREF_152)] | 2024 | Assessment of data quality and associated factors in the routine health information system among health workers in public health institutions of Gofa Zone, Southern Ethiopia: A mixed methods study | Excluded by title |
| Döll, M.[[153](#_ENREF_153)] | 2012 | Making People Breathe: A Case Study of the Shanghai Social Assistance Programme Di Bao | Excluded by title |
| Douglas, D. B. et al[[154](#_ENREF_154)] | 2019 | Meeting the World Health Organization Maternal Antenatal Care Guidelines Is Associated with Improved Early and Middle Childhood Cognition in Ethiopia | Excluded by title |
| Douthit, N. T. & Alemu, H. K.[[155](#_ENREF_155)] | 2016 | Social determinants of health: poverty, national infrastructure and investment | Excluded by abstract |
| Dutton, D. B.[[156](#_ENREF_156)] | 1978 | Explaining the low use of health services by the poor: costs, attitudes, or delivery systems? | Excluded by title |
| Dzakpasu, S. et al[[157](#_ENREF_157)] | 2014 | Impact of user fees on maternal health service utilization and related health outcomes: a systematic review | Excluded by title |
| Eggleston, K. et al[[158](#_ENREF_158)] | 2008 | Health service delivery in China: a literature review | Excluded by title |
| El Arifeen, S. et al[[159](#_ENREF_159)] | 2013 | Community-based approaches and partnerships: innovations in health-service delivery in Bangladesh | Excluded by title |
| El-Khoury, M. et al[[160](#_ENREF_160)] | 2012 | User fee exemptions and equity in access to caesarean sections: an analysis of patient survey data in Mali | Excluded by title |
| Enawgaw, B. et al[[161](#_ENREF_161)] | 2019 | Prevalence of Anemia and Iron Deficiency Among Pregnant Women Attending Antenatal Care Service at University of Gondar Hospital, Northwest Ethiopia | Excluded by title |
| Endriyas, M.[[162](#_ENREF_162)] | 2022 | Quality of medical records in public health facilities: A case of Southern Ethiopia, resource limited setting | Excluded by title |
| Engdawork, K. & Sintayehu, F.[[163](#_ENREF_163)] | 2023 | The State and Transformation of Female Wage Labour in Ethiopia: The Case of Textile/Garment Industries | Excluded by title |
| Ensor, T. & Ronoh, J.[[164](#_ENREF_164)] | 2005 | Effective financing of maternal health services: a review of the literature | Excluded by title |
| Estensen, M. E. et al[[165](#_ENREF_165)] | 1997 | Fighting the good fight in Ethiopia | Excluded by title |
| Etemadi, M. & Hajizadeh, M.[[166](#_ENREF_166)] | 2022 | User fee removal for the poor: a qualitative study to explore policies for social health assistance in Iran | Excluded by title |
| Ezra, M. & Kiros, G. E.[[167](#_ENREF_167)] | 2000 | Household vulnerability to food crisis and mortality in the drought-prone areas of northern Ethiopia | Excluded by title |
| Fink, E. L. et al[[168](#_ENREF_168)] | 2018 | Traumatic Brain Injury and Infectious Encephalopathy in Children From Four Resource-Limited Settings in Africa | Excluded by title |
| Fikre, R. & Fiche, Y. A.[[169](#_ENREF_169)] | 2017 | Provider's Knowledge and Availability of Emergency Obstetrics and Newborn Care Service in Case of Gedeo Zone, Southern Ethiopia | Excluded by title |
| Fikre, R. et al[[170](#_ENREF_170)] | 2024 | Correlates of adverse outcomes of adolescent pregnancy in Sidama region, Ethiopia. An unmatched case-control study | Excluded by title |
| Franzen, S. R. P. et al[[171](#_ENREF_171)] | 2017 | Strategies for developing sustainable health research capacity in low and middle-income countries: a prospective, qualitative study investigating the barriers and enablers to locally led clinical trial conduct in Ethiopia, Cameroon and Sri Lanka | Excluded by title |
| Freeman, T. et al[[172](#_ENREF_172)] | 2020 | Why do some countries do better or worse in life expectancy relative to income? An analysis of Brazil, Ethiopia, and the United States of America | Excluded by abstract |
| Gadisa, L. M.[[173](#_ENREF_173)] | 2022 | Decentralization and health sector reform: lessons from Ethiopia | Excluded by abstract |
| Gan Jingwen, G. Y.[[174](#_ENREF_174)] | 2024 | Research and Effect Evaluation of Internal Performance Management Practice in Community Health Service Center in a District of Beijing | Excluded by title |
| Gandhi K. et al[[175](#_ENREF_175)] | 2018 | Racial disparities in health service utilization among Medicare fee-for-service beneficiaries adjusting for multiple chronic conditions | Outside Ethiopia |
| Ganle, J. K. et al[[176](#_ENREF_176)] | 2014 | A qualitative study of health system barriers to accessibility and utilization of maternal and newborn healthcare services in Ghana after user-fee abolition | Excluded by title |
| Gans, J.[[177](#_ENREF_177)] | 2012 | Debates on US Immigration | Excluded by title |
| Gebrehiwot, T. & van der Veen A.[[178](#_ENREF_178)] | 2014 | Coping with food insecurity on a micro-scale: evidence from Ethiopian rural households | Excluded by title |
| Gebremariam, A. et al[[179](#_ENREF_179)] | 2021 | Delayed initiation of adjuvant chemotherapy among women with breast cancer in Addis Ababa, Ethiopia | Excluded by title |
| Gebru, T. & Lentiro, K.[[180](#_ENREF_180)] | 2018 | The impact of community-based health insurance on health-related quality of life and associated factors in Ethiopia: a comparative cross-sectional study | Excluded by title |
| Geleto, A. et al[[181](#_ENREF_181)] | 2018 | Barriers to access and utilization of emergency obstetric care at health facilities in sub-Saharan Africa: a systematic review of literature | Excluded by title |
| Genene, E.[[182](#_ENREF_182)] | 2018 | Assessment of private wing practice in all African leprosy rehabilitation center | Excluded by title |
| Gesese, K. T.[[183](#_ENREF_183)] | 2015 | Migration and socio-demographic determinants of women’s reproductive health services utilization in North Gondar, Ethiopia | Excluded by abstract |
| Geta, E. T. et al[[184](#_ENREF_184)] | 2022 | Does community-based health insurance reduce disparities in modern health service utilization among households in Ethiopia? A community-based comparative cross-sectional study | Excluded by abstract |
| Getachew, N.[[185](#_ENREF_185)] | 2023 | Catastrophic health expenditure and associated factors among households of non community based health insurance districts, Ilubabor zone, Oromia regional state, southwest Ethiopia | Excluded by abstract |
| Getahun, T. et al[[186](#_ENREF_186)] | 2022 | Magnitude of community-based health insurance utilization and associated factors in Bassona Worena District, North Shoa Zone, Ethiopia: a community-based cross-sectional study | Excluded by title |
| Getaneh, Z. et al[[187](#_ENREF_187)] | 2019 | Prevalence and determinants of stunting and wasting among public primary school children in Gondar town, northwest, Ethiopia | Excluded by title |
| Getiye, Y. & Fantahun, M.[[188](#_ENREF_188)] | 2017 | Factors associated with perinatal mortality among public health deliveries in Addis Ababa, Ethiopia, an unmatched case control study | Excluded by title |
| Gilson, L. & McIntyre, D.[[189](#_ENREF_189)] | 2005 | Removing user fees for primary care in Africa: the need for careful action | Excluded by title |
| Girishankar, N. [[190](#_ENREF_190)] | 2001 | "Governance and poverty reduction," Poverty Reduction Strategy Sourcebook | Excluded by title |
| Gilson, L. et al[[191](#_ENREF_191)] | 1995 | The political economy of user fees with targeting: developing equitable health financing policy | Excluded by title |
| Girma, B.[[190](#_ENREF_190)] | 2010 | Assessment of Fee Waiver Health Care Implementation Status in the New Health Care Financing Strategy in Bahir Dar, North West Ethiopia | No clear outcome measurement |
| Girmaw, F.et al [[192](#_ENREF_192)] | 2023 | Willingness to Pay for Social Health Insurance Among Health Care Professionals in North Wollo Zone, Amhara Region, Ethiopia: Mixed Method Study | Excluded by title |
| Girmay A. M. & Reta, M. T.[[193](#_ENREF_193)] | 2022 | Community‐based health insurance service utilization and associated factors in Addis Ababa, Ethiopia | Excluded by title |
| Gjerde, J. L. et al[[194](#_ENREF_194)] | 2013 | Silently waiting to heal: experiences among women living with urinary incontinence in northwest Ethiopia | Excluded by title |
| Goodwin, S. M. & Anderson, G. F.[[195](#_ENREF_195)] | 2012 | Effect of cost-sharing reductions on preventive service use among Medicare fee-for-service beneficiaries | Outside Ethiopia |
| Gordon-Strachan, G. et al[[196](#_ENREF_196)] | 2010 | The impact of user fees for Preventive Health Care—Jamaica | Excluded by title |
| Gotsadze, G. et al[[197](#_ENREF_197)] | 2005 | Health care-seeking behaviour and out-of-pocket payments in Tbilisi, Georgia | Excluded by title |
| Goudge, J. et al[[198](#_ENREF_198)] | 2009 | Affordability, availability and acceptability barriers to health care for the chronically ill: longitudinal case studies from South Africa | Excluded by title |
| Grosh, M. et al[[199](#_ENREF_199)] | 2022 | Unpacking the empirics of targeting in low-and middle-income countries | Excluded by title |
| Grosh, M. et al[[200](#_ENREF_200)] | 2008 | For protection and promotion: The design and implementation of effective safety nets | Excluded by title |
| Guda, A.[[201](#_ENREF_201)] | 2008 | Challenges of Healthcare Financing: Economic and Welfare Effects of User Fees in Urban Ethiopia | Excluded by abstract |
| Gulta, F. T. et al[[202](#_ENREF_202)] | 2024 | Patient satisfaction with pharmacy services among users and non users of community based health insurance scheme at public health facilities in Gamo Zone, South Ethiopia: a comparative cross sectional study | Excluded by title |
| Guo, A.[[203](#_ENREF_203)] | 2012 | User fees in primary healthcare in Sub-Saharan Africa: A study of the effects and legacy of World Bank neoliberal health polic | Excluded by title |
| Gurmu, E. & Mace, R.[[204](#_ENREF_204)] | 2008 | Fertility decline driven by poverty: the case of Addis Ababa, Ethiopia | Excluded by title |
| Guta, A. et al[[205](#_ENREF_205)] | 2021 | Utilization of modern contraceptives and associated factors among street women in Dire Dawa, Eastern Ethiopia: a mixed study | Excluded by title |
| Gwatkin, D. R.[[206](#_ENREF_206)] | 2012 | Paying for health care: moving beyond the user-fee debate | Excluded by title |
| Hadley, C. et al[[207](#_ENREF_207)] | 2012 | Rapidly rising food prices and the experience of food insecurity in urban Ethiopia: impacts on health and well-being | Excluded by title |
| Hagen-Zanker, J. & Holmes, R.[[208](#_ENREF_208)] | 2012 | Social protection in Nigeria, Synthesis report | Excluded by title |
| Hailegebreal, S. et al[[209](#_ENREF_209)] | 2019 | Alcohol control policy in Ethiopia and implications for public health | Excluded by title |
| Hailemariam, M. et al[[210](#_ENREF_210)] | 2016 | Equitable access to integrated primary mental healthcare for people with severe mental disorders in Ethiopia: a formative study | Excluded by title |
| Hailemichael, Y.[[211](#_ENREF_211)] | 2019 | Catastrophic health expenditure and impoverishment in households of persons with depression: a cross-sectional, comparative study in rural Ethiopia | Excluded by title |
| Hailemichael, Y. et al[[212](#_ENREF_212)] | 2019 | Mental health problems and socioeconomic disadvantage: a controlled household study in rural Ethiopia | Excluded by title |
| Hailu, A. et al[[213](#_ENREF_213)] | 2021 | Equity in public health spending in Ethiopia: a benefit incidence analysis | Excluded by abstract |
| Hailu, A. G. & Amare, Z. Y.[[214](#_ENREF_214)] | 2022 | Impact of productive safety net program on food security of beneficiary households in western Ethiopia: A matching estimator approach | Excluded by title |
| Hailu, K. et al[[215](#_ENREF_215)] | 2022 | Barriers to cleaning of shared latrines in slums of Addis Ababa, Ethiopia | Excluded by title |
| Hammad, M. et al[[216](#_ENREF_216)] | 2021 | Next practices: Innovations in the COVID-19 social protection responses and beyond | Excluded by abstract |
| Hamren, K. et al[[217](#_ENREF_217)] | 2015 | Religion, spirituality, social support and quality of life: measurement and predictors CASP-12(v2) amongst older Ethiopians living in Addis Ababa | Excluded by title |
| Hamza, Y. A. et al[[218](#_ENREF_218)] | 2023 | The impact of community-based health insurance on universal health coverage in Ethiopia: a systematic review and meta-analysis," | Excluded by title |
| Hangoma, P.[[219](#_ENREF_219)] | 2017 | Social Protection, Health Risk, and Household Welfare in Zambia | Excluded by title |
| Hanlon, C. et al[[220](#_ENREF_220)] | 2016 | Task sharing for the care of severe mental disorders in a low-income country (TaSCS): study protocol for a randomised, controlled, non-inferiority trial | Excluded by title |
| Hardeman, W. et al[[221](#_ENREF_221)] | 2004 | Access to health care for all? User fees plus a Health Equity Fund in Sotnikum, Cambodia | Excluded by title |
| Harris, B. et al[[222](#_ENREF_222)] | 2011 | Inequities in access to health care in South Africa | Excluded by title |
| Hauge, S. W. & Dalen, H.[[223](#_ENREF_223)] | 2021 | Short-term outcome after open-heart surgery for severe chronic rheumatic heart disease in a low-income country, with comparison with an historical control group: an observational study | Excluded by title |
| Henriques, J.[[224](#_ENREF_224)] | 1996 | Bearing the brunt of poverty | Excluded by title |
| Hensley, E.[[225](#_ENREF_225)] | 2020 | The Refugee Economy in Jordan |  |
| Hill, R. et al[[226](#_ENREF_226)] | 2017 | Fiscal incidence analysis for Ethiopia," The Distributional Impact of Fiscal Policy: Experience from Developing Countries | Excluded by title |
| Hirvonen, K. et al[[227](#_ENREF_227)] | 2017 | Complementarities between social protection and health sector policies: Evidence from the productive safety net program in Ethiopia | Excluded by abstract |
| Hjellbakk, V. K. et al[[228](#_ENREF_228)] | 2022 | Diet and nutritional status among hospitalised children in Hawassa, Southern Ethiopia | Excluded by title |
| Holmemo, C. et al [[229](#_ENREF_229)] | 2020 | Investing in People: Social Protection for Indonesia's 2045 Vision, | Excluded by title |
| Holmes, R. et al[[230](#_ENREF_230)] | 2012 | Social protection in Nigeria, Mapping programmes and their effectiveness. Overseas Development Institution (ODI) | Excluded by title |
| Huang Jinling, Z. Z.[[231](#_ENREF_231)] | 2023 | The Logic and Trend of Urban Community Health Service Policies in China | Excluded by title |
| Huffman, S.[[232](#_ENREF_232)] | 1988 | Women, work and pregnancy outcome | Excluded by title |
| Hurlburt, S. et al[[233](#_ENREF_233)] | 2020 | Assessing medical impoverishment and associated factors in health care in Ethiopia | Excluded by title |
| Hussien, M. et al[[234](#_ENREF_234)] | 2022 | Financial viability of a community-based health insurance scheme in two districts of northeast Ethiopia: a mixed methods study | Excluded by title |
| Hutton, G.[[235](#_ENREF_235)] | 2004 | Charting the path to the World Bank’s “No blanket policy on user fees”: a look over the past 25 years at the shifting support for user fees in health and education, and reflections on the future | Excluded by abstract |
| Hutton, G.[[236](#_ENREF_236)] | 2004 | "Is the jury still out on the impact of user fees in Africa? A review of the evidence from selected countries on user fees and determinants of health service utilisation | Excluded by title |
| Jacobs, B. & Price, A.[[237](#_ENREF_237)] | 2008 | A comparative study of the effectiveness of pre-identification and passive identification for hospital fee waivers at a rural Cambodian hospital | Excluded by title |
| Jacobs, B. & Price, N.[[238](#_ENREF_238)] | 2004 | The impact of the introduction of user fees at a district hospital in Cambodia | Excluded by title |
| Jacobs, E. et al[[239](#_ENREF_239)] | 2020 | Performance-based financing, basic packages of health services and user-fee exemption mechanisms: an analysis of health-financing policy integration in three fragile and conflict-affected settings | Excluded by title |
| James, C. et al[[240](#_ENREF_240)] | _ | To Retain or Remove User Fees? Reflections on the Current Debate | Excluded by title |
| Jehu-Appiah, C. et al[[241](#_ENREF_241)] | 2011 | Equity aspects of the National Health Insurance Scheme in Ghana: Who is enrolling, who is not and why? | Excluded by title |
| Jiahua, S. H. I. et al[[242](#_ENREF_242)] | 2022 | Current health service demands of new residents in shanghai | Excluded by title |
| Jingyi, Q. et al[[243](#_ENREF_243)] | 2023 | Progress in researches on disease burden and health service utilization of beta-thalassemia patients in China | Excluded by title |
| Jiwani, S. S. et al[[244](#_ENREF_244)] | 2020 | Trends and inequalities in the nutritional status of adolescent girls and adult women in sub-Saharan Africa since 2000: a cross-sectional series study | Excluded by title |
| Johansson, K. A.et al[[245](#_ENREF_245)] | 2017 | Health Gains and Financial Protection Provided by the Ethiopian Mental Health Strategy: an Extended Cost-Effectiveness Analysis | Excluded by title |
| John, E. U. | 2013 | The impacts of user fees on health services in sub-Saharan African countries: a ctirical analysis of the evidence | Excluded by title |
| Johnson, F. A. et al[[246](#_ENREF_246)] | 2016 | Two decades of maternity care fee exemption policies in Ghana: have they benefited the poor? | Excluded by title |
| Jones, N. & Pincock, K.[[247](#_ENREF_247)] | 2020 | Intersecting inequalities, gender and adolescent health in Ethiopia | Excluded by title |
| Karan, A. et al[[248](#_ENREF_248)] | 2017 | Extending health insurance to the poor in India: An impact evaluation of Rashtriya Swasthya Bima Yojana on out of pocket spending for healthcare | Excluded by title |
| Karim, A. M. et al[[249](#_ENREF_249)] | 2015 | Changes in equity of maternal, newborn, and child health care practices in 115 districts of rural Ethiopia: implications for the health extension program | Excluded by abstract |
| Kassa, A. M.[[250](#_ENREF_250)] | 2023 | In Ethiopia's Kutaber district, does community-based health insurance protect households from catastrophic health-care costs? A community- based comparative cross-sectional study | Excluded by title |
| Kassa, B. A. et al[[251](#_ENREF_251)] | 2018 | Opportunities and barriers to implementing antibiotic stewardship in low and middle-income countries: Lessons from a mixed-methods study in a tertiary care hospital in Ethiopia | Excluded by title |
| Kifle, M. et al[[252](#_ENREF_252)] | 2010 | Transfer and adoption of advanced information technology solutions in resource-poor environments: the case of telemedicine systems adoption in Ethiopia | Excluded by title |
| Khun, S. & Manderson, L.[[253](#_ENREF_253)] | 2008 | Poverty, user fees and ability to pay for health care for children with suspected dengue in rural Cambodia | Excluded by title |
| Kiima, D. & Jenkins, R.[[254](#_ENREF_254)] | 2010 | Mental health policy in Kenya-an integrated approach to scaling up equitable care for poor populations | Excluded by title |
| Kiros, M. et al[[255](#_ENREF_255)] | 2020 | The burden of household out-of-pocket health expenditures in Ethiopia: estimates from a nationally representative survey | Excluded by title |
| Klotz, S. A. et al | 2009 | Immune reconstitution inflammatory syndrome in a resource-poor setting | Excluded by title |
| Koch, S. F.[[256](#_ENREF_256)] | 2017 | User fee abolition and the demand for public health care | Excluded by title |
| Korachais, C. et al[[257](#_ENREF_257)] | 2019 | The impact of reimbursed user fee exemption of health centre outpatient consultations for the poor in pluralistic health systems: lessons from a quasi-experiment in two rural health districts in Cambodia | Excluded by title |
| Kruk, M. E. et al[[258](#_ENREF_258)] | 2008 | User fee exemptions are not enough: out‐of‐pocket payments for ‘free’delivery services in rural Tanzania | Excluded by title |
| Kumsa, M. J.[[259](#_ENREF_259)] | 2020 | Lack of pocket money impacts Ethiopian undergraduate health science students learning activities | Excluded by title |
| Koon, A. D.[[260](#_ENREF_260)] | 2022 | Aligning priorities in Ethiopian health finance: How do the essential health services package and health benefit plans compare? | Excluded by abstract |
| Kurji, J. et al[[261](#_ENREF_261)] | 2020 | Uncovering spatial variation in maternal healthcare service use at subnational level in Jimma Zone, Ethiopia | Excluded by title |
| La Forgia, G. M. & Griffin, C. C.[[262](#_ENREF_262)] | 1992 | Cost recovery in public hospitals in Belize: Health Financing and Sustainability Project | Excluded by title |
| Lambin, R. & Nyyssölä, M.[[263](#_ENREF_263)] | 2022 | Two decades of Tanzanian health policy: Examining policy developments and opportunities through a gender lens | Excluded by title |
| Langenbrunner, J. C. &Tandon, A.[[264](#_ENREF_264)] | 2012 | Health financing systems in East Asia and the Pacific: early successes and current challenges | Excluded by title |
| Laokri, S. et al[[265](#_ENREF_265)] | 2013 | Removal of user fees no guarantee of universal health coverage: observations from Burkina Faso | Excluded by title |
| Lapointe, M.[[266](#_ENREF_266)] | 2020 | Urbanization and human-nature relationships: a comparison of urban and rural dwellers' perceptions of ecosystem services in the Solomon Islands | Excluded by title |
| Lee, A. C.[[267](#_ENREF_267)] | 2009 | Linking families and facilities for care at birth: what works to avert intrapartum-related deaths? | Excluded by title |
| Lekashingo, L. D.[[268](#_ENREF_268)] | 2012 | Exploring the effects of user fees, quality of care and utilization of health services on enrolment in community health fund, Bagamoyo district, Tanzania | Excluded by title |
| Leighton, C.[[269](#_ENREF_269)] | 1995 | 22 Policy Questions About Health Care Financing in Africa | Excluded by title |
| Lemu, B. N. et al[[270](#_ENREF_270)] | 2020 | Inequalities in measles immunization coverage in Ethiopia: a cross-sectional analysis of demographic and health surveys 2000-2016 | Excluded by abstract |
| Leone, T. et al[[271](#_ENREF_271)] | 2016 | Financial accessibility and user fee reforms for maternal healthcare in five sub-Saharan countries: a quasi-experimental analysis | Excluded by title |
| M. Lewis[[272](#_ENREF_272)] | 2007 | Informal payments and the financing of health care in developing and transition countries | Excluded by title |
| Li, Y. et al[[273](#_ENREF_273)] | 2012 | Factors affecting catastrophic health expenditure and impoverishment from medical expenses in China: policy implications of universal health insurance | Excluded by title |
| Liang, C. et al[[274](#_ENREF_274)] | 2023 | Framework and Application Strategy of Smart Proactive Health Service | Excluded by title |
| Lindelöw, M. & Wagstaff, A.[[275](#_ENREF_275)] | 2003 | Health facility surveys: an introduction | Excluded by title |
| Lindstrom, D. P. & Berhanu, B.[[276](#_ENREF_276)] | 1999 | The impact of war, famine, and economic decline on marital fertility in Ethiopia | Excluded by title |
| Linnander, E. et al[[277](#_ENREF_277)] | 2016 | Use of a national collaborative to improve hospital quality in a low-income setting | Excluded by title |
| Litvack, J. I. & Bodart, C.[[278](#_ENREF_278)] | 1993 | User fees plus quality equals improved access to health care: results of a field experiment in Cameroon | Excluded by title |
| Loewe, M.[[279](#_ENREF_279)] | 2020 | Community effects of cash-for-work programmes in Jordan: supporting social cohesion, more equitable gender roles and local economic development in contexts of flight and migration | Excluded by title |
| Lothe, E. A. & Heggen, K.[[280](#_ENREF_280)] | 2003 | A study of resilience in young Ethiopian famine survivors | Excluded by title |
| Louw, G. & Duvenhage, A.[[281](#_ENREF_281)] | 2017 | Are the fees that the traditional health practitioner charges generally lower than that of the medical practitioner? | Excluded by title |
| Lufumpa, E. et al[[282](#_ENREF_282)] | 2018 | Barriers and facilitators to preventive interventions for the development of obstetric fistulas among women in sub-Saharan Africa: a systematic review | Excluded by title |
| Lukman, H. Y. & Ramadan, A. T.[[283](#_ENREF_283)] | 2003 | Critical appraisal of the law enforcement in abortion care in Ethiopia | Excluded by title |
| Maeda, A.et al[[284](#_ENREF_284)] | 2014 | Universal health coverage for inclusive and sustainable development: a synthesis of 11 country case studies | Excluded by title |
| Mahmood, S. S[[285](#_ENREF_285)]. | 2015 | Micro Health Insurance in Bangladesh: Prospects and Challenges | Excluded by title |
| Maina, T. & Onguti, E.[[286](#_ENREF_286)] | 2014 | Health Policy and Abolition of User Fees at Public Primary Healthcare Facilities | Excluded by title |
| Malenbaum, W.[[287](#_ENREF_287)] | 1973 | Health and economic expansion in poor lands | Excluded by title |
| Mall, S. et al[[288](#_ENREF_288)] | 2017 | Restoring the person's life': a qualitative study to inform development of care for people with severe mental disorders in rural Ethiopia | Excluded by title |
| Mamdani, B.[[289](#_ENREF_289)] | 2007 | Alternatives to user fees for public health care | Excluded by title |
| Mamo, A. et al[[290](#_ENREF_290)] | 2017 | Developing a measure of mental health service satisfaction for use in low income countries: a mixed methods study | Excluded by title |
| Marcus, R. et al[[291](#_ENREF_291)] | 2011 | Children and social protection in the Middle East and North Africa | Excluded by title |
| María, B. A. & Heflinger, C. A.[[292](#_ENREF_292)] | 2005 | Child behavioral health service use and caregiver strain: Comparison of managed care and fee-for-service Medicaid systems | Excluded by title |
| Marselian, Z. M.[[293](#_ENREF_293)] | 2020 | Studying the Impact of an Integrated Service Model on the Health and Well-Being of Vulnerable Populations | Excluded by title |
| Martin, M. & Stulgaitis, M.[[294](#_ENREF_294)] | 2022 | Refugees’ access to higher education in their host countries: Overcoming the ‘super-disadvantage | Excluded by title |
| Martin C. M. & Smith, D. G.[[295](#_ENREF_295)] | 2005 | The impact of donkey ownership on the livelihoods of female peri-urban dwellers in Ethiopia | Excluded by title |
| Marye, D. M. et al[[296](#_ENREF_296)] | 2023 | User Fee Exemption Policy Significantly Improved Adherence to Maternal Health Service Utilization in Bahir Dar City, Northwest Ethiopia: A Comparative Cross-Sectional Study | Excluded by title |
| Masinjila, M.[[297](#_ENREF_297)] | 2020 | Policy mapping: women’s economic empowerment in Kenya | Excluded by title |
| Masiye, F. et al[[298](#_ENREF_298)] | 2008 | Removal of user fees at primary health care facilities in Zambia: a study of the effects on utilisation and quality of care | Excluded by title |
| Masiye, F. et al[[299](#_ENREF_299)] | 2010 | From targeted exemptions to user fee abolition in health care: experience from rural Zambia | Excluded by title |
| Maweu, K. C. et al[[300](#_ENREF_300)] | 2011 | Assessment of the Constituency Development Fund in Enhancing KCPE Performance of Public Primary Schools in Kenya: A Case of Mwala Constituency | Excluded by title |
| Mbindandonyi, J. [[301](#_ENREF_301)] | 2021 | Utilization of free skilled birth attendance services among women of reproductive age in public health facilities in Kitui County, Kenya | Excluded by title |
| McCord, A.[[302](#_ENREF_302)] | 2013 | Community-based targeting in the Social Protection sector | Excluded by abstract |
| McIntyre, D.[[303](#_ENREF_303)] | 2018 | Challenges in financing universal health coverage in sub-Saharan Africa | Excluded by title |
| McKay, R.[[304](#_ENREF_304)] | 2010 | Post-social prescriptions: Medical welfare in Mozambique | Excluded by title |
| McKnight, J. & Holt, D. B.[[305](#_ENREF_305)] | 2014 | Designing the Expanded Programme on Immunisation (EPI) as a service: Prioritising patients over administrative logic | Excluded by title |
| McIntyre, D. et al[[306](#_ENREF_306)] | 2006 | What are the economic consequences for households of illness and of paying for health care in low-and middle-income country contexts? | Excluded by title |
| McPake, B.[[307](#_ENREF_307)] | 1993 | User charges for health services in developing countries: a review of the economic literature | Excluded by title |
| McPake, B. et al[[308](#_ENREF_308)] | 2008 | Freeing-up Healthcare: A guide to removing user fees | Excluded by title |
| Mebratie, A.[[309](#_ENREF_309)] | 2015 | Essays on evaluating a community based health insurance scheme in rural Ethiopia | Excluded by title |
| Mebratie, A. et al[[310](#_ENREF_310)] | 2014 | The impact of Ethiopia’s pilot community based health insurance scheme on healthcare utilization and cost of care | Excluded by title |
| Mebratie, A. et al[[311](#_ENREF_311)] | 2014 | Dropping out of Ethiopia’s Community Based Health Insurance scheme | Excluded by title |
| Mebratie, A. et al[[312](#_ENREF_312)] | 2013 | Self-reported health care seeking behavior in rural Ethiopia: evidence from clinical vignettes | Excluded by abstract |
| Mebratie, A. D. et al[[313](#_ENREF_313)] | 2024 | Willingness of urban formal sector workers to support a community-based health insurance scheme in Ethiopia | Excluded by title |
| Mebratie, A. D.[[314](#_ENREF_314)] | 2015 | Enrollment in Ethiopia’s community-based health insurance scheme | Excluded by title |
| Meessen, B. et al[[315](#_ENREF_315)] | 2011 | User fee removal in low-income countries: sharing knowledge to support managed implementation | Excluded by title |
| Meessen, B. et al[[316](#_ENREF_316)] | 2011 | Removing user fees in the health sector: a review of policy processes in six sub-Saharan African countries | Excluded by title |
| Meessen, B. et al[[317](#_ENREF_317)] | 2006 | Poverty and user fees for public health care in low-income countries: lessons from Uganda and Cambodia | Excluded by title |
| Mekasha, K. M.[[318](#_ENREF_318)] | 2015 | Assessment of Budget Preparation and Utilization: Case of Addis Ababa City Administration Health Bureau | Excluded by title |
| Mekonen, A. M. et al[[319](#_ENREF_319)] | 2018 | The effect of community based health insurance on catastrophic health expenditure in Northeast Ethiopia: A cross sectional study | Excluded by title |
| Mekonnen, A. & Jones, N.[[320](#_ENREF_320)] | 2005 | Tackling child malnutrition in Ethiopia | Excluded by title |
| Mekonnen, G. B. & Gelayee, D. A.[[321](#_ENREF_321)] | 2020 | Low Medication Knowledge and Adherence to Oral Chronic Medications among Patients Attending Community Pharmacies: A Cross-Sectional Study in a Low-Income Country | Excluded by title |
| Mekonnen, W. &. Dechassa, W[[322](#_ENREF_322)] | 2024 | Inter-district and Wealth-related Inequalities in Maternal and Child Health Service Coverage and Child Mortality within Addis Ababa City | Excluded by abstract |
| Melaku, Z. et al[[323](#_ENREF_323)] | 2006 | Pattern of admissions to the medical intensive care unit of Addis Ababa University Teaching Hospital | Excluded by title |
| Melchert, T. P.[[324](#_ENREF_324)] | 2020 | Foundations of Health Service Psychology : an evidence-based biopsychosocial approach, Second edition. ed. London | Excluded by title |
| Melese, A. T.[[325](#_ENREF_325)] | 2017 | Living Wage Report Non-Metropolitan Urban Ethiopia Ziway Region: Context Provided in the Horticulture Sector | Excluded by title |
| Melesse, D. Y. et al[[326](#_ENREF_326)] | 2020 | Why some women who attend focused antenatal care fail to deliver in health facilities: a qualitative study of women's perspectives from slums of Addis Ababa, Ethiopia | Excluded by title |
| Memirie, S. T. et al[[327](#_ENREF_327)] | 2016 | Inequalities in utilization of maternal and child health services in Ethiopia: the role of primary health care | Excluded by abstract |
| Merene, Y. et al[[328](#_ENREF_328)] | 2023 | Ground and tiger beetles (Coleoptera: Carabidae, Cicindelidae) of the Federal Democratic Republic of Ethiopia: a provisional faunistic checklist based on literature data | Excluded by title |
| Mersha, D.[[329](#_ENREF_329)] | 2015 | The Contribution of Heath Care Financing for Enhancing Drug Supply Capacity in Public Health Centers in Addis Ababa in the case of Bole Sub city | Excluded by abstract |
| Mesele, A. G. et al[[330](#_ENREF_330)] | 2024 | District health information system 2 data utilization among health professionals in Amara region private hospitals, Ethiopia | Excluded by title |
| Miles, S. & Malone, J. L.[[331](#_ENREF_331)] | 2013 | Perspectives from Ethiopia regarding U.S. military humanitarian assistance: how to build a better medical civil action project (MEDCAP) | Excluded by title |
| Mills, A. et al[[332](#_ENREF_332)] | 2012 | Equity in financing and use of health care in Ghana, South Africa, and Tanzania: implications for paths to universal coverage | Excluded by title |
| Misgina, K. H. et al[[333](#_ENREF_333)] | 2021 | Pre-conception and prenatal factors influencing gestational weight gain: a prospective study in Tigray region, northern Ethiopia | Excluded by title |
| Mojumdar, S. K.[[334](#_ENREF_334)] | 2015 | Trends in Maternal Care Utilization in Urban India: A Temporal Analysis | Excluded by title |
| Molla, M. et al[[335](#_ENREF_335)] | 2015 | Impacts of maternal mortality on living children and families: A qualitative study from Butajira, Ethiopia | Excluded by title |
| Moszynski, P.[[336](#_ENREF_336)] | 2006 | Zambia scraps healthcare fees for poor rural people | Excluded by title |
| Moyehodie, Y. A. et al[[337](#_ENREF_337)] | 2022 | Factors associated with community based health insurance healthcare service utilization of households in South Gondar zone, Amhara, Ethiopia. A community-based cross-sectional study | Excluded by title |
| Muche E. T. et al[[338](#_ENREF_338)] | 2022 | Diabetes mellitus and its association with central obesity, and overweight/obesity among adults in Ethiopia. A systematic review and meta-analysis | Excluded by title |
| Mugisha, J. O. et al[[339](#_ENREF_339)] | 2017 | Timing of most recent health care visit by older people living with and without HIV: findings from the SAGE well-being of older people study in Uganda, | Excluded by title |
| Munene, W. K. et al[[340](#_ENREF_340)] | 2024 | Implementation of User Fee Policy in Psychiatric Hospitals in Kenya: A Case of Mathari Hospital, Nairobi, | Excluded by title |
| Munishi, V. [[341](#_ENREF_341)] | 2010 | Assessment of user fee system: implementation of exemption and waiver mechanisms in Tanzania: successes and challenges | Excluded by title |
| Musau, A. M.[[342](#_ENREF_342)] | 2019 | Choice Of Place Of Birth In The Era Of The Maternity Subsidy In Kenya, University of Nairobi, Kenya | Excluded by title |
| Mussa, E. C. et al[[343](#_ENREF_343)] | 2023 | Impact of community-based health insurance on health services utilisation among vulnerable households in Amhara region, Ethiopia, | Excluded by title |
| Mustapher, M. H.[[344](#_ENREF_344)] | 2023 | Impacts of National Health Insurance Fund revenue on quality health service delivery at Dodoma City in Tanzania, The Open University of Tanzania | Excluded by title |
| Mwangi, D. & Jonah, M.[[345](#_ENREF_345)] | 2017 | Effect of the Free Maternity Programme on the Access and Outcomes of Maternal and Newborn Health (MNH) In the County of Kiambu | Excluded by title |
| Naeye, R. L. et al[[346](#_ENREF_346)] | 1977 | Amniotic fluid infections in an African city | Excluded by title |
| Negash, S. et al[[347](#_ENREF_347)] | 2024 | Correlation of Pediatric Surgical Infrastructure With Clinical and Economic Outcomes: A Cohort Study | Excluded by title |
| Nagelkerke, N. J.& De Vlas, S. J.[[348](#_ENREF_348)] | 2003 | The epidemiological impact of an HIV vaccine on the HIV/AIDS epidemic in Southern India | Excluded by title |
| Nenko, G.[[349](#_ENREF_349)] | 2017 | Assessment of free health service provision system in Dilla town, South Ethiopia | No clear outcome measurement |
| Ngwacho, G. A.[[350](#_ENREF_350)] | 2015 | Effects of hidden costs in free secondary education on transition and completion rates in public boarding schools in Kisii County, Kenya | Excluded by title |
| Nielsen, J. K. H.[[351](#_ENREF_351)] | 2021 | Brain Drain in Ethiopia’s Health Sector: Perceptions of and experiences with medical brain drain among Ethiopian health workers | Excluded by title |
| Nirmala Ravishankar, C. M. et al[[352](#_ENREF_352)] | 2014 | Health Financing Analysis for Countdown Case Studies | Excluded by title |
| Normand, E.[[353](#_ENREF_353)] | 2024 | OG-7-5 Sculpting Surgical Safety: The Transformative | Excluded by title |
| Obse, A. et al[[354](#_ENREF_354)] | 2015 | Knowledge of and preferences for health insurance among formal sector employees in Addis Ababa: a qualitative study | Excluded by title |
| Obse, A. G. & Ataguba, J. E.[[355](#_ENREF_355)] | 2020 | Assessing medical impoverishment and associated factors in health care in Ethiopia | Excluded by title |
| Obse, A. G. & Ataguba, J. E.[[356](#_ENREF_356)] | 2020 | Assessing catastrophic out-of-pocket payments in Ethiopia’s health system | Excluded by title |
| Obse, A. et al[[357](#_ENREF_357)] | 2016 | Eliciting preferences for social health insurance in Ethiopia: a discrete choice experiment | Excluded by title |
| Ocho, D. L. et al[[358](#_ENREF_358)] | 2012 | Assessing the levels of food shortage using the traffic light metaphor by analyzing the gathering and consumption of wild food plants, crop parts and crop residues in Konso, Ethiopia | Excluded by title |
| Ogundele, O.[[359](#_ENREF_359)] | 2020 | Inequalities in the use of reproductive health care in Sub-Saharan Africa: evidence from Ghana and Nigeria | Excluded by title |
| Oomman, N. et al[[360](#_ENREF_360)] | 2003 | Inequalities in health, nutrition and population | Excluded by abstract |
| Oomman, N. et al[[361](#_ENREF_361)] | 2007 | Inequalities in health and population | Excluded by abstract |
| Onarheim, K. H. et al[[362](#_ENREF_362)] | 2017 | What if the baby doesn't survive? Health-care decision making for ill newborns in Ethiopia | Excluded by title |
| Onarheim, K. H. et al[[363](#_ENREF_363)] | 2018 | Selling my sheep to pay for medicines - household priorities and coping strategies in a setting without universal health coverage | Excluded by abstract |
| W. H. Organization[[364](#_ENREF_364)] | 2002 | Health, economic growth and poverty reduction: the report of Working Group I of the Commission on Macroeconomics and Health | Excluded by title |
| W. H. Organization[[365](#_ENREF_365)] | 2003 | Building on the user-fee experience: The African case | Excluded by title |
| W. H. Organization[[366](#_ENREF_366)] | 2017 | Transformative accountability for adolescents: accountability for the health and human rights of women, children and adolescents in the 2030 agenda | Excluded by title |
| Oyugi, B. et al[[367](#_ENREF_367)] | 2024 | Examining the quality of care across the continuum of maternal care (antenatal, perinatal and postnatal care) under the expanded free maternity policy (Linda Mama Policy) in Kenya: a mixed-methods study | Excluded by title |
| Pearson, L.[[368](#_ENREF_368)] | 2011 | User fees and maternity services in Ethiopia | Excluded by abstract |
| Pearson, M. & Chandler, C.[[369](#_ENREF_369)] | 2019 | Knowing antmicrobial resistance in practice: a multi-country qualitative study with human and animal healthcare professionals | Excluded by title |
| Percival, V. et al[[370](#_ENREF_370)] | 2014 | Health systems and gender in post-conflict contexts: building back better? | Excluded by title |
| Pouw, N. & Bender, K.[[371](#_ENREF_371)] | 2022 | The poverty reduction effect of social protection: the pros and cons of a multidisciplinary approach | Excluded by title |
| Bradt, D. A. & Drummond, C. M.[[372](#_ENREF_372)] | 2016 | Epidemic preparedness and response | Excluded by title |
| Quentin, W. et al[[373](#_ENREF_373)] | 2014 | Inequalities in child mortality in ten major African cities | Excluded by title |
| Quimbo, S. et al[[374](#_ENREF_374)] | 2014 | Long-term effects of a randomized policy experiment on quality improvement in the Philippines | Excluded by title |
| Rad, M. H. et al[[375](#_ENREF_375)] | 2024 | Disparities in satisfaction among insured and uninsured adult outpatient department service users in Southern Ethiopia, 2022: a comparative cross-sectional study | Excluded by title |
| Rakotomalala, M. et al[[376](#_ENREF_376)] | 2019 | Complete Genome Sequences of Rice Yellow Mottle Virus Isolates from the Federal Democratic Republic of Ethiopia | Excluded by title |
| Radhika, M.[[377](#_ENREF_377)] | 2017 | Knowledge and Practice of Auxiliary Nurse Midwives (ANMS) Regarding Reproductive and Child Health Services | Excluded by title |
| Ravindran, T. S. & Govender, V. [[378](#_ENREF_378)] | 2020 | Sexual and reproductive health services in universal health coverage: a review of recent evidence from low-and middle-income countries | Excluded by title |
| Rickart, A. J. et al[[379](#_ENREF_379)] | 2020 | Facing Africa: Describing Noma in Ethiopia | Excluded by title |
| Robinson, A.[[380](#_ENREF_380)] | 2012 | Sanitation Finance in Rural Cambodia: Water and Sanitation Program | Excluded by title |
| Sado, E. & Gedif, T.[[381](#_ENREF_381)] | 2014 | Drug utilization at household level in Nekemte Town and surrounding rural areas, western Ethiopia: a cross-sectional study, | Excluded by abstract |
| Samuels, F. et al[[382](#_ENREF_382)] | 2012 | HIV vulnerabilities and the potential for strengthening social protection responses in the context of HIV in Nigeria | Excluded by title |
| Sanbata, H. et al[[383](#_ENREF_383)] | 2014 | Association of biomass fuel use with acute respiratory infections among under- five children in a slum urban of Addis Ababa, Ethiopia | Excluded by title |
| Scott, N. A.[[384](#_ENREF_384)] | 2013 | Orphans in Zambia: Program monitoring and evaluation practices and the association of external support with education status and psychosocial wellbeing | Excluded by title |
| Sdralevich, M. C. A. et al[[385](#_ENREF_385)] | 2014 | Subsidy reform in the Middle East and North Africa: Recent progress and challenges ahead | Excluded by title |
| Seery, E. et al[[386](#_ENREF_386)] | 2020 | From Catastrophe to Catalyst: Can the World Bank make COVID-19 a turning point for building universal and fair public healthcare systems? | Excluded by title |
| Seid, M. et al[[387](#_ENREF_387)] | 2021 | Willingness to pay for cataract surgery and associated factors among cataract patients in Outreach Site, North West Ethiopia | Excluded by title |
| Selenica, E.[[388](#_ENREF_388)] | 2018 | Universities between the State and the Market: Development Policy, Commercialization and Liberalization in Higher Education | Excluded by title |
| Semahagn, S.[[389](#_ENREF_389)] | 2023 | Health Centers Pharmacy Service Reform Practice and Associated factors among Health Professionals in Central Gondar Zone, North West Ethiopia | Excluded by title |
| Semahegn, A. & Mengistie, B.[[390](#_ENREF_390)] | 2015 | Domestic violence against women and associated factors in Ethiopia; systematic review | Excluded by title |
| Semrau, M. et al[[391](#_ENREF_391)] | 2015 | Strengthening mental health systems in low- and middle-income countries: the Emerald programme | Excluded by title |
| Sendo, E. G.[[392](#_ENREF_392)] | 2021 | Women's perspectives on the measures that need to be taken to increase the use of health-care facility delivery service among slums women, Addis Ababa, Ethiopia: a qualitative study | Excluded by title |
| Shahvisi, A. et al[[393](#_ENREF_393)] | 2018 | A Human Right to Shoes? Establishing Rights and Duties in the Prevention and Treatment of Podoconiosis | Excluded by title |
| Shega, S. A.et al[[394](#_ENREF_394)] | 2013 | Exploring factors affecting Health Extension Workers motivation in selected urban, rural and pastoralist districts of Ethiopia | Excluded by title |
| Shepherd, A. et al[[395](#_ENREF_395)] | 2011 | Addressing chronic poverty and vulnerability through social assistance in Tanzania: Assessing the options | Excluded by title |
| Shigute, Z. et al[[396](#_ENREF_396)] | 2023 | Social protection schemes in Ethiopia: the productive safety net program and the community based health insurance scheme | Excluded by title |
| Shigute, Z. et al[[397](#_ENREF_397)] | 2019 | The use and significance of vaccination cards | Excluded by title |
| Shimels, T. et al[[398](#_ENREF_398)] | 2021 | Magnitude and associated factors of poor medication adherence among diabetic and hypertensive patients visiting public health facilities in Ethiopia during the COVID-19 pandemic | Excluded by title |
| Shrime, M. G. et al[[399](#_ENREF_399)] | 2016 | Task-sharing or public finance for the expansion of surgical access in rural Ethiopia: an extended cost-effectiveness analysis | Excluded by title |
| Spangler, S. A. et al[[400](#_ENREF_400)] | 2014 | An evaluation of equitable access to a community-based maternal and newborn health program in rural Ethiopia | Excluded by title |
| Spitzer, H. & Mabeyo, M.[[401](#_ENREF_401)] | 2011 | In search of protection: Older people and their fight for survival in Tanzania | Excluded by title |
| Steurs, L.[[402](#_ENREF_402)] | 2019 | European aid and health system strengthening: an analysis of donor approaches in the DRC, Ethiopia, Uganda, Mozambique and the global fund | Excluded by title |
| Sumit, S.[[403](#_ENREF_403)] | 2006 | User fee Experience in Madhya Pradesh under Rogi Kalyan Samiti | Excluded by title |
| Sundaram, A. et al[[404](#_ENREF_404)] | 2010 | Benefits of meeting the contraceptive needs of Ethiopian women | Excluded by title |
| Tadesse, M. et al[[405](#_ENREF_405)] | 2015 | Countdown to 2015: Ethiopia's progress towards reduction in under-five mortality: 2014 country case study | Excluded by title |
| Taddesse, G. et al[[406](#_ENREF_406)] | 2023 | Spatial variation and predictors of missing birth preparedness and complication readiness (BPCR) messages in Ethiopia | Excluded by title |
| Tamiru, D. et al[[407](#_ENREF_407)] | 2015 | Developing a mental health care plan in a low resource setting: the theory of change approach | Excluded by title |
| Tarekegn, W. et al[[408](#_ENREF_408)] | 2022 | Skilled birth attendant utilization trends, determinant and inequality gaps in Ethiopia | Excluded by abstract |
| Tasic, H. et al[[409](#_ENREF_409)] | 2020 | Drivers of stunting reduction in Ethiopia: a country case study | Excluded by title |
| Taye, B. et al[[410](#_ENREF_410)] | 2013 | Podoconiosis and soil-transmitted helminths (STHs): double burden of neglected tropical diseases in Wolaita zone, rural Southern Ethiopia | Excluded by title |
| Tedla, M. et al[[411](#_ENREF_411)] | 2024 | Prevalence and associated factors of malnutrition among under-five children living in slum areas of Bahir Dar Town, Ethiopia | Excluded by title |
| Teferra, A. S. & Bergen, N.[[412](#_ENREF_412)] | 2018 | Perceptions and experiences related to health and health inequality among rural communities in Jimma Zone, Ethiopia: a rapid qualitative assessment | Excluded by title |
| Tegegne, A. & Legese, M.[[413](#_ENREF_413)] | 2014 | The Urban Poor and Health Seeking Behavior: The Healthcare Seeking Behavior of the ‘Poorest of the Poor’in Addis Ababa | Excluded by abstract |
| Tekelab, T. et al[[414](#_ENREF_414)] | 2015 | Predictors of modern contraceptive methods use among married women of reproductive age groups in Western Ethiopia: a community based cross-sectional study | Excluded by title |
| Teklehaimanot, H. D. & Teklehaimanot, A.[[415](#_ENREF_415)] | 2013 | Human resource development for a community-based health extension program: a case study from Ethiopia | Excluded by title |
| Temesgen, D. et al[[416](#_ENREF_416)] | 2022 | Utilization of Maternal Health Services in Western Ethiopia: A Community-Based Cross-Sectional Study | Excluded by abstract |
| Tenaw, Y.[[417](#_ENREF_417)] | 2017 | Analysis of factors influencing individual’s willingness to pay for the compulsory social health insurance scheme: The case of government shool teachers in Kolfe Keraniyo subcity | Excluded by title |
| Tenaw, Z. et al[[418](#_ENREF_418)] | 2022 | Determinants of maternity waiting home utilization in Sidama Zone, Southern Ethiopia: A cross-sectional study | Excluded by abstract |
| Teni, F. S. et al[[419](#_ENREF_419)] | 2018 | Costs incurred by outpatients at a university hospital in northwestern Ethiopia: a cross-sectional study | Excluded by title |
| Tesfaye, K. et al[[420](#_ENREF_420)] | 2018 | Assessment of Free Health Care Provision System in North Gondar Gondar Town, Ethiopia | Excluded by abstract |
| Teshome, Y. G.[[421](#_ENREF_421)] | 2017 | Maternal health in Ethiopia: Global and Local complexities | Excluded by title |
| Thomas, P.[[422](#_ENREF_422)] | 2005 | Ending child poverty and securing child rights: the role of social protection | Excluded by title |
| Tibebe, A. et al[[423](#_ENREF_423)] | 2012 | Examining out of pocket payments for maternal health in rural Ethiopia: paradox of free health care un-affordability | Excluded by abstract |
| Tigabu, Y. T. et al[[424](#_ENREF_424)] | 2023 | Rural household income mobility in Ethiopia: Dimensions, drivers and policy | Excluded by title |
| Tilahun, D. et al[[425](#_ENREF_425)] | 2016 | Stigma, explanatory models and unmet needs of caregivers of children with developmental disorders in a low-income African country: a cross-sectional facility-based survey | Excluded by title |
| Tirfessa, K. et al[[426](#_ENREF_426)] | 2019 | Food insecurity among people with severe mental disorder in a rural Ethiopian setting: a comparative, population-based study | Excluded by title |
| Tirfessa, K. et al[[427](#_ENREF_427)] | 2020 | Impact of integrated mental health care on food insecurity of households of people with severe mental illness in a rural African district: a community-based, controlled before-after study | Excluded by title |
| Tiyou, A. et al[[428](#_ENREF_428)] | 2012 | Food insecurity and associated factors among HIV-infected individuals receiving highly active antiretroviral therapy in Jimma zone Southwest Ethiopia | Excluded by title |
| Tjarks, B. A.[[429](#_ENREF_429)] | 2017 | Maternal Mortality in the Developing World–Simple Solutions Aren’t Enough | Excluded by title |
| Tolla, M. T.[[430](#_ENREF_430)] | 2018 | Prevention and treatment of cardiovascular disease in Ethiopia saves more than lives: cost-effectiveness analysis, extended cost-effectiveness analysis, and financial risk protection | Excluded by title |
| Tolla, M. T. et al[[431](#_ENREF_431)] | 2017 | Out-of-pocket expenditures for prevention and treatment of cardiovascular disease in general and specialised cardiac hospitals in Addis Ababa, Ethiopia: a cross-sectional cohort study | Excluded by title |
| Tomlin, K. et al[[432](#_ENREF_432)] | 2020 | Assessing capacity of health facilities to provide routine maternal and newborn care in low-income settings: what proportions are ready to provide good-quality care, and what proportions of women receive it? | Excluded by abstract |
| Trabitzsch, J. et al[[433](#_ENREF_433)] | 2024 | Understanding referral of patients with cancer in rural Ethiopia: a qualitative study | Excluded by title |
| Tsega, Y. et al[[434](#_ENREF_434)] | 2024 | Insured-non-insured disparity of catastrophic health expenditure in Northwest Ethiopia: a multivariate decomposition analysis | Excluded by title |
| Tsega, Y. et al[[435](#_ENREF_435)] | 2023 | Leaving no one behind in health: financial hardship to access health care in Ethiopia | Excluded by abstract |
| Tuoyire, D. A. et al[[436](#_ENREF_436)] | 2024 | Healthcare utilization in Ghana: Insights from the 2017 Ghana Living Standard Survey | Excluded by title |
| UNICEF [[437](#_ENREF_437)] | 2007 | Enhanced Protection for Children Affected by AIDS | Excluded by title |
| USAID[[438](#_ENREF_438)] | 2007 | Guidelines for supportive supervision in the health sector | Excluded by title |
| Verguet, S. et al[[439](#_ENREF_439)] | 2016 | Assessing the burden of medical impoverishment by cause: a systematic breakdown by disease in Ethiopia | Excluded by title |
| Verguet, S. et al[[440](#_ENREF_440)] | 2015 | Health gains and financial risk protection afforded by public financing of selected interventions in Ethiopia: an extended cost-effectiveness analysis | Excluded by abstract |
| Vogel, J. P. et al[[441](#_ENREF_441)] | 2016 | Barriers, Facilitators and Priorities for Implementation of WHO Maternal and Perinatal Health Guidelines in Four Lower-Income Countries: A GREAT Network Research Activity | Excluded by title |
| Wakayo, T. et al[[442](#_ENREF_442)] | 2016 | Vitamin D Deficiency is Associated with Overweight and/or Obesity among Schoolchildren in Central Ethiopia: A Cross-Sectional Study | Excluded by title |
| Wako, W. G. & Kassa, D. H.[[443](#_ENREF_443)] | 2017 | Institutional delivery service utilization and associated factors among women of reproductive age in the mobile pastoral community of the Liban District in Guji Zone, Oromia, Southern Ethiopia: a cross sectional study | Excluded by abstract |
| Wang, H. & Ramana, G.[[444](#_ENREF_444)] | 2014 | Universal health coverage for inclusive and sustainable development, Country Summary Report for Ethiopia | Excluded by title |
| Wang, H. & Rosemberg, N.[[445](#_ENREF_445)] | 2018 | Universal health coverage in low-income countries: Tanzania’s efforts to overcome barriers to equitable health service access | Excluded by title |
| Weiner C. M. et al[[446](#_ENREF_446)] | 2018 | Characteristics and follow-up of metastatic breast cancer in Ethiopia: A cohort study of 573 women | Excluded by title |
| Wellum, J.[[447](#_ENREF_447)] | 2014 | Critical analysis of the Kenyan healthcare system and models for improvement | Excluded by title |
| Wibaek, R. et al[[448](#_ENREF_448)] | 2019 | Higher Weight and Weight Gain after 4 Years of Age Rather than Weight at Birth Are Associated with Adiposity, Markers of Glucose Metabolism, and Blood Pressure in 5-Year-Old Ethiopian Children | Excluded by title |
| Wickremasinghe, D. et al[[449](#_ENREF_449)] | 2016 | District decision-making for health in low-income settings: a systematic literature review | Excluded by title |
| Wiggins, S. et al[[450](#_ENREF_450)] | 2020 | Policy Interventions to Mitigate Negative Effects on Poverty, Agriculture and Food Security, from Disease Outbreaks and Other Crises | Excluded by title |
| Willis, C. Y. & Leighton, C.[[451](#_ENREF_451)] | 1995 | Protecting the poor under cost recovery: the role of means testing," Health policy and planning | Excluded by title |
| Woldehanna, T. et al[[452](#_ENREF_452)] | 2011 | Understanding Changes in the Lives of Poor Children | Excluded by title |
| Woldie, M. et al[[453](#_ENREF_453)] | 2022 | Resource Mobilisation and allocation for primary health care: lessons from the Ethiopian health system | Excluded by title |
| Wondawek, T. M. & Ali, M. M.[[454](#_ENREF_454)] | 2019 | Delay in treatment seeking and associated factors among suspected pulmonary tuberculosis patients in public health facilities of Adama town, eastern Ethiopia | Excluded by title |
| Workicho, A. et al[[455](#_ENREF_455)] | 2016 | Household dietary diversity and Animal Source Food consumption in Ethiopia: evidence from the 2011 Welfare Monitoring Survey | Excluded by title |
| Worku, B. N. et al[[456](#_ENREF_456)] | 2018 | Effects of home-based play-assisted stimulation on developmental performances of children living in extreme poverty: a randomized single-blind controlled trial | Excluded by title |
| Worku, B. N. et al[[457](#_ENREF_457)] | 2018 | The relationship of undernutrition/psychosocial factors and developmental outcomes of children in extreme poverty in Ethiopia | Excluded by title |
| Yakob, B. & Ncama, B. P.[[458](#_ENREF_458)] | 2017 | Measuring health system responsiveness at facility level in Ethiopia: performance, correlates and implications | Excluded by title |
| Wright, C. & Govinda, R. | 1994 | Three years after Jomtien | Excluded by title |
| Wussobo, A. M. | 2014 | Health and Poverty: The Issue of Health Inequalities in Ethiopia | Excluded by abstract |
| Xu, Q.[[459](#_ENREF_459)] |  | Resident participation and community organization in China: Impacts and consequences of China's urban community services | Excluded by title |
| Yawkal, T.[[460](#_ENREF_460)] | 2022 | Financial Hardship of Healthcare and Associated factors Among Households in Debretabor Town, Amhara, Northwest Ethiopia | Excluded by abstract |
| Yeneabat, T. et al[[461](#_ENREF_461)] | 2019 | Maternal dietary diversity and micronutrient adequacy during pregnancy and related factors in East Gojjam Zone, Northwest Ethiopia | Excluded by title |
| Yilma, Z. et al[[462](#_ENREF_462)] | 2021 | Economic Consequences of Ill-Health in Rural Ethiopia | Excluded by title |

# References

[1] G. Abate, W. Kogi-Makau, and N. M. Muroki, "Health seeking and hygiene behaviours predict nutritional status of pre-school children in a slum area of Addis Ababa, Ethiopia," PLoS One, vol. 38, pp. 253-65, Oct 2000.

[2] S. Abayneh, H. Lempp, A. Alem, D. Alemayehu, T. Eshetu, C. Lund, et al., "Service user involvement in mental health system strengthening in a rural African setting: qualitative study," vol. 17, p. 187, May 18 2017.

[3] W. Abdallah, S. Chowdhury, and K. Iqbal, "Access and fees in public health care services for the poor: Bangladesh as a case study," Oxford Development Studies, vol. 50, pp. 209-224, 2022.

[4] S. G. Abdela, E. Diro, F. T. Zewdu, F. T. Berhe, W. E. Yeshaneh, K. S. Tamirat, et al., "Looking for NTDs in the skin; an entry door for offering patient centered holistic care," J Infect Dev Ctries, vol. 14, pp. 16s-21s, Jun 29 2020.

[5] M. M. Abdou Illou, S. Haddad, I. Agier, and V. Ridde, "The elimination of healthcare user fees for children under five substantially alleviates the burden on household expenses in Burkina Faso," BMC health services research, vol. 15, pp. 1-11, 2015.

[6] F. Abebaw, A. Atalay, H. Charlotte, L. Crick, H. Damen, C. Dan, et al., "Catastrophic health expenditure and impoverishment in households of persons with depression: a cross-sectional, comparative study in rural Ethiopia," BMC Public Health, vol. 19, pp. 1-13, 2019.

[7] T. G. Abessa, B. N. Worku, M. W. Kibebew, J. Valy, J. Lemmens, H. Thijs, et al., "Adaptation and standardization of a Western tool for assessing child development in non-Western low-income context," BMC Public Health, vol. 16, p. 652, Jul 28 2016.

[8] B. Abel-Smith and P. Rawal, "Can the poor afford ‘free’health services? A case study of Tanzania," Health Policy and Planning, vol. 7, pp. 329-341, 1992.

[9] A. ABEBE, "The Study on Effectiveness of Community Based Micro Health Insurance Scheme attached with Women Self Help GROUP (SHG) approach in Ethiopia: the Case of Jimma Town Women SHGs," St. Mary's University, 2014.

[10] Z. Abebe, "The Contribution of Community Based Health Insurance (CBHI) in Improving Access and Utilization of Healthcare Services: The Case of Adea District, East Shoa Zone, Oromia Region, Ethiopia," St. Mary’s University, 2018.

[11] J. Abelson and J. Lomas, "Do health service organizations and community health centres have higher disease prevention and health promotion levels than fee-for-service practices?," CMAJ: Canadian Medical Association Journal, vol. 142, p. 575, 1990.

[12] O. Abrahim, E. Linnander, H. Mohammed, N. Fetene, and E. Bradley, "A Patient-Centered Understanding of the Referral System in Ethiopian Primary Health Care Units," PLoS One, vol. 10, p. e0139024, 2015.

[13] D. Abu, M. Agajie, T. Sahilu, and C. Strupat, "Willingness to take COVID-19 vaccination in low-income countries: Evidence from Ethiopia," Environ Health Prev Med, vol. 17, p. e0264633, 2022.

[14] S. Accorsi, G. A. Barnabas, P. Farese, V. Padovese, M. Terranova, V. Racalbuto, et al., "Skin disorders and disease profile of poverty: analysis of medical records in Tigray, northern Ethiopia, 2005-2007," Trans R Soc Trop Med Hyg, vol. 103, pp. 469-75, May 2009.

[15] S. Accorsi, N. Kedir, P. Farese, S. Dhaba, V. Racalbuto, A. Seifu, et al., "Poverty, inequality and health: the challenge of the double burden of disease in a non-profit hospital in rural Ethiopia," Trans R Soc Trop Med Hyg, vol. 103, pp. 461-8, May 2009.

[16] M. A. Adabre and A. P. Chan, "Critical success factors (CSFs) for sustainable affordable housing," Building and Environment, vol. 156, pp. 203-214, 2019.

[17] K. Adane, Z. Abiy, and K. Desta, "The revenue generated from clinical chemistry and hematology laboratory services as determined using activity-based costing (ABC) model," Cost Effectiveness and Resource Allocation, vol. 13, pp. 1-7, 2015.

[18] M. Adane, B. Mengistie, G. Medhin, H. Kloos, and W. Mulat, "Piped water supply interruptions and acute diarrhea among under-five children in Addis Ababa slums, Ethiopia: A matched case-control study," PLoS One, vol. 12, p. e0181516, 2017.

[19] M. Adane, B. Mengistie, W. Mulat, H. Kloos, and G. Medhin, "Utilization of health facilities and predictors of health-seeking behavior for under-five children with acute diarrhea in slums of Addis Ababa, Ethiopia: a community-based cross-sectional study," J Health Popul Nutr, vol. 36, p. 9, Apr 4 2017.

[20] A. A. Adish, S. A. Esrey, T. W. Gyorkos, and T. Johns, "Risk factors for iron deficiency anaemia in preschool children in northern Ethiopia," Public Health Nutr, vol. 2, pp. 243-52, Sep 1999.

[21] T. W. Admassu, Y. T. Wolde, and M. Kaba, "Ethiopia has a long way to go meeting adolescent and youth sexual reproductive health needs," Reprod Health, vol. 19, p. 130, Jun 13 2022.

[22] R. Afkar, "Public Services, Social Protection, and Poverty," Universitäts-und Landesbibliothek Bonn, 2016.

[23] A. Agegnehu and A. Behaylu, "Health and Decentralization: The Case of Gozamin District, Amhara State, Ethiopia," Health, vol. 5, 2015.

[24] I. A. Agyepong, "Reforming health service delivery at district level in Ghana: the perspective of a district medical officer," Health policy and planning, vol. 14, pp. 59-69, 1999.

[25] S. Ahmed, L. E. Chase, J. Wagnild, N. Akhter, S. Sturridge, A. Clarke, et al., "Community health workers and health equity in low-and middle-income countries: systematic review and recommendations for policy and practice," International Journal for Equity in Health, vol. 21, p. 49, 2022.

[26] S. Ahmed, A. A. Creanga, D. G. Gillespie, and A. O. Tsui, "Economic status, education and empowerment: implications for maternal health service utilization in developing countries," PloS one, vol. 5, p. e11190, 2010.

[27] A. I. Ajayi, "“I am alive; my baby is alive”: understanding reasons for satisfaction and dissatisfaction with maternal health care services in the context of user fee removal policy in Nigeria," PloS one, vol. 14, p. e0227010, 2019.

[28] A. I. Ajayi and W. Akpan, "Who benefits from free institutional delivery? evidence from a cross sectional survey of North Central and Southwestern Nigeria," BMC Health Services Research, vol. 17, pp. 1-10, 2017.

[29] A. Alebachew, Y. Yusuf, C. Mann, and P. Berman, "Ethiopia’s Progress in health financing and the contribution of the 1998 health care and financing strategy in Ethiopia," MA, Addis Ababa: Harvard TH Chan School of Public Health and Breakthrough International Consultancy, PLC, vol. 95, 2015.

[30] A. Alem, "Mental health services and epidemiology of mental health problems in Ethiopia," Ethiop Med J, vol. 39, pp. 153-65, Apr 2001.

[31] Y. M. Alemu, F. Ambaw, and A. Wilder-Smith, "Utilization of HIV testing services among pregnant mothers in low income primary care settings in northern Ethiopia: a cross sectional study," BMC Pregnancy Childbirth, vol. 17, p. 199, Jun 24 2017.

[32] G. D. Alene and A. Worku, "Examining perceptions of rapid population growth in North and South Gondar zones, northwest Ethiopia," J Health Popul Nutr, vol. 27, pp. 784-93, Dec 2009.

[33] M. Alene, L. Yismaw, Y. Berelie, and B. Kassie, "Health care utilization for common childhood illnesses in rural parts of Ethiopia: evidence from the 2016 Ethiopian demographic and health survey," BMC Public Health, vol. 19, p. 57, Jan 14 2019.

[34] E. E. Ali, "Health care financing in Ethiopia: implications on access to essential medicines," Value in health regional issues, vol. 4, pp. 37-40, 2014.

[35] E. E. Ali, A.-H. Gilani, and T. Gedif, "Pharmaceutical pricing in Ethiopia," in Pharmaceutical prices in the 21st century, ed: Springer, 2014, pp. 79-91.

[36] G. O. Amarech and E. A. John, "Assessing medical impoverishment and associated factors in health care in Ethiopia," BMC International Health and Human Rights, vol. 20, pp. 1-9, 2020.

[37] M. Amexo, R. Tolhurst, G. Barnish, and I. Bates, "Malaria misdiagnosis: effects on the poor and vulnerable," The Lancet, vol. 364, pp. 1896-1898, 2004.

[38] G. Ameya, T. Tsalla, F. Getu, and E. Getu, "Antimicrobial susceptibility pattern, and associated factors of Salmonella and Shigella infections among under five children in Arba Minch, South Ethiopia," Ann Clin Microbiol Antimicrob, vol. 17, p. 1, Feb 1 2018.

[39] E. Amporfu, "Effect of regulated user fee on quality of healthcare for the poor and the non-poor," International Review of Economics, vol. 60, pp. 357-373, 2013.

[40] E. Amporfu, "A theoretical analysis of how user fee on healthcare can waste economic resources," Health, vol. 6, pp. 128-136, 2014.

[41] A. Anangwe, "Health sector reforms in Kenya: User fees," Sama, Martyn/Nguyen, Vinh-Kim (Hg.). Governing health systems in Africa. Council for the Development of Social Science Research in Africa (COSRIA), Dakar: S, pp. 44-59, 2008.

[42] J. R. Anderson, Rural extension services vol. 2976: World Bank Publications, 2003.

[43] G. F. Anderson, "From ‘soak the rich’to ‘soak the poor’: recent trends in hospital pricing," Health Affairs, vol. 26, pp. 780-789, 2007.

[44] E. Alessandra Cancedda, O. R. Jaromir Hurnik, E. Corrado Minardi, E. Jonathan Wolsey, and E. Amin Abdella, "Mitigating the socio-economic impacts of COVID-19 in Ethiopia, with a focus on vulnerable groups," 2020.

[45] M. D. Argaw, B. F. Desta, M. A. Kibret, M. G. Abebe, W. K. Heyi, E. Mamo, et al., "Accelerating the performance of district health systems towards achieving UHC via twinning partnerships," BMC Health Serv Res, vol. 20, p. 892, Sep 21 2020.

[46] D. Arhin-Tenkorang, "Mobilizing resources for health: the case for user fees revisited," CID Working Paper Series, 2001.

[47] C. Arsenault, B. Yakob, M. Kassa, G. Dinsa, and S. Verguet, "Using health management information system data: case study and verification of institutional deliveries in Ethiopia," vol. 6, Aug 2021.

[48] A. D. Aserese, A. Atenafu, M. Sisay, M. B. Sorrie, B. W. Yirdaw, and M. K. Zegeye, "Adequate vitamin A rich food consumption and associated factors among lactating mothers visiting child immunization and post-natal clinic at health institutions in Gondar Town, Northwest Ethiopia," PLoS One, vol. 15, p. e0239308, 2020.

[49] L. Asher, C. Hanlon, R. Birhane, A. Habtamu, J. Eaton, H. A. Weiss, et al., "Community-based rehabilitation intervention for people with schizophrenia in Ethiopia (RISE): a 12 month mixed methods pilot study," BMC Psychiatry, vol. 18, p. 250, Aug 3 2018.

[50] I. M. Asker and A. M. Nuh, "Utilization of health service and associated factors among women in childbearing age in selected internally displaced persons camps, Hargeisa–Somaliland," Open Health, vol. 5, p. 20230023, 2024.

[51] E. M. Assefa, "Knowledge, attitude and practice (KAP) of health providers towards safe abortion provision in Addis Ababa health centers," BMC Womens Health, vol. 19, p. 138, Nov 14 2019.

[52] N. Assefa, Y. Berhane, A. Worku, and A. Tsui, "The hazard of pregnancy loss and stillbirth among women in Kersa, East Ethiopia: a follow up study," Sex Reprod Healthc, vol. 3, pp. 107-12, Oct 2012.

[53] R. Atakoğlu Yılmaz, S. Türen, and D. İstengir, "Challenges and Affecting Factors for Nurses in the Integration of Home Health Service and Palliative Care," CURARE Journal of Nursing, pp. 23-30, 2023.

[54] N. Atchessi, V. Ridde, and M.-V. Zunzunegui, "User fees exemptions alone are not enough to increase indigent use of healthcare services," Health Policy and Planning, vol. 31, pp. 674-681, 2016.

[55] R. A. Atinga, S. A. Mensah, F. Asenso-Boadi, and F.-X. A. Adjei, "Migrating from user fees to social health insurance: exploring the prospects and challenges for hospital management," BMC health services research, vol. 12, pp. 1-10, 2012.

[56] R. Atun, C. Chaumont, J. R. Fitchett, A. Haakenstad, and D. Kaberuka, "Poverty alleviation and the economic benefits of investing in health," in Harvard TH Chan, Harvard Kennedy School, Ministerial Leadership, Forum for Finance Ministers, 2016, pp. 1-26.

[57] B. I. Avan, D. Berhanu, N. Umar, D. Wickremasinghe, and J. Schellenberg, "District decision-making for health in low-income settings: a feasibility study of a data-informed platform for health in India, Nigeria and Ethiopia," Health Policy Plan, vol. 31 Suppl 2, pp. ii3-ii11, Sep 2016.

[58] D. G. Ayele, T. T. Zewotir, and H. G. Mwambi, "Semiparametric models for malaria rapid diagnosis test result," BMC Public Health, vol. 14, p. 31, Jan 13 2014.

[59] M. Azage and D. Haile, "Factors affecting healthcare service utilization of mothers who had children with diarrhea in Ethiopia: evidence from a population based national survey," Rural Remote Health, vol. 15, p. 3493, 2015.

[60] D. M. Badasu, "Implementation of Ghana’s health user fee policy and the exemption of the poor: problems and prospects," 2004.

[61] S. Baird, W. Yadete, J. Hamory Hicks, and P. F. D. Scheelbeek, "Improving the use of focus group discussions in low income settings," Int J Equity Health, vol. 20, p. 287, Nov 30 2020.

[62] D. Balabanova, M. McKee, J. Pomerleau, R. Rose, and C. Haerpfer, "Health service utilization in the former Soviet Union: evidence from eight countries," Health services research, vol. 39, pp. 1927-1950, 2004.

[63] I. Barnett and B. Tefera, Poor Households’ Experiences and Perception of User Fees for Healthcare: a mixed-method study from Ethiopia: Young Lives, 2010.

[64] E. C. Baron, C. Hanlon, S. Mall, S. Honikman, E. Breuer, T. Kathree, et al., "Maternal mental health in primary care in five low- and middle-income countries: a situational analysis," BMC Health Serv Res, vol. 16, p. 53, Feb 16 2016.

[65] E. C. Baron, S. D. Rathod, C. Hanlon, M. Prince, A. Fedaku, F. Kigozi, et al., "Impact of district mental health care plans on symptom severity and functioning of patients with priority mental health conditions: the Programme for Improving Mental Health Care (PRIME) cohort protocol," BMC Psychiatry, vol. 18, p. 61, Mar 6 2018.

[66] A. Barrientos and R. Smith, "Social Assistance in Low Income Countries Database," London: Department for International Development (DFID), 2005.

[67] R. Baru, A. Acharya, S. Acharya, A. S. Kumar, and K. Nagaraj, "Inequities in access to health services in India: caste, class and region," Economic and political Weekly, pp. 49-58, 2010.

[68] P. BARUA, P. Barua, and K. Narattharaksa, "Investigation into health disparities among stateless children in Tak Province, Thailand," Naresuan University, 2019.

[69] R. K. Basaza, B. Criel, and P. Van der Stuyft, "Community health insurance amidst abolition of user fees in Uganda: the view from policy makers and health service managers," BMC Health services research, vol. 10, pp. 1-10, 2010.

[70] A. Baschieri and J. Hobson, "Support for the Ethiopian Health Sector Development Programme (Federal Ministry of Health MDG Performance Fund)," 2013.

[71] K. Baye, "Prioritizing the Scale-Up of Evidence-Based Nutrition and Health Interventions to Accelerate Stunting Reduction in Ethiopia," Nutrients, vol. 11, Dec 16 2019.

[72] E. M. Bayked, H. N. Toleha, S. Zewdie, A. M. Mekonen, B. D. Workneh, and M. H. Kahissay, "Beneficiaries’ satisfaction with community-based health insurance services and associated factors in Ethiopia: a systematic review and meta-analysis," Cost Effectiveness and Resource Allocation, vol. 22, pp. 1-12, 2024.

[73] Y. T. Bayou, Y. S. Mashalla, and G. Thupayagale-Tshweneagae, "The adequacy of antenatal care services among slum residents in Addis Ababa, Ethiopia," BMC Pregnancy Childbirth, vol. 16, p. 142, Jun 15 2016.

[74] Y. Beaugé, "Targeted User Fee Exemption for Equitable Access to Primary Healthcare Services for the Ultra-Poor: A multi-method study using the case of Burkina Faso," 2022.

[75] A. Bedaso, Y. Abraham, A. Temesgen, and N. Mekonnen, "Quality of sleep and associated factors among people living with HIV/AIDS attending ART clinic at Hawassa University comprehensive specialized Hospital, Hawassa, SNNPR, Ethiopia," PLoS One, vol. 15, p. e0233849, 2020.

[76] J. Bedford, "Qualitative research to identify solutions to local barriers to care-seeking and treatment for diarrhoea, malaria and pneumonia in select high burden countries," 2012.

[77] M. Bekele, M. Getnet, A. Getachew, Z. Shewamene, M. Abraha, N. Morrow, et al., "Farm forests, seasonal hunger, and biomass poverty: Evidence of induced intensification from panel data in the Ethiopian Highlands," PLoS One, vol. 53, pp. 435-451, Mar 2024.

[78] M. Belay and W. Deressa, "Use of insecticide treated nets by pregnant women and associated factors in a pre-dominantly rural population in northern Ethiopia," Trop Med Int Health, vol. 13, pp. 1303-13, Oct 2008.

[79] M. Belew, D. Kebede, M. Kassaye, and F. Enquoselassie, "The magnitude of khat use and its association with health, nutrition and socio-economic status," Ethiop Med J, vol. 38, pp. 11-26, Jan 2000.

[80] Y. Berhan, "Predictors of perinatal mortality associated with placenta previa and placental abruption: an experience from a low income country," J Pregnancy, vol. 2014, p. 307043, 2014.

[81] F. Berhane Defaye and M. Danis, "Bedside Rationing Under Resource Constraints-A National Survey of Ethiopian Physicians' Use of Criteria for Priority Setting," vol. 10, pp. 125-135, Apr-Jun 2019.

[82] Y. Berhane, Y. Gossaye, M. Emmelin, and U. Hogberg, "Women's health in a rural setting in societal transition in Ethiopia," Soc Sci Med, vol. 53, pp. 1525-39, Dec 2001.

[83] T. Berhan, M. Meseret, and H. Mizan, "Catastrophic out-of-pocket payments related to non-communicable disease multimorbidity and associated factors, evidence from a public referral hospital in Addis Ababa Ethiopia," BMC Health Services Research, vol. 24, pp. 1-13, 2024.

[84] M. Berk and L. M. McGivern, "Effects of a Facilitated Fee Waiver Program on Participation in Youth Sports Programs," Journal of Park & Recreation Administration, vol. 34, 2016.

[85] T. Besley and S. Coate, "Public provision of private goods and the redistribution of income," The American Economic Review, vol. 81, pp. 979-984, 1991.

[86] A. Beyene, T. Hailu, K. Faris, and H. Kloos, "Current state and trends of access to sanitation in Ethiopia and the need to revise indicators to monitor progress in the Post-2015 era," BMC Public Health, vol. 15, p. 451, May 2 2015.

[87] S. Bhattacharyya, D. Berhanu, N. Taddesse, A. Srivastava, D. Wickremasinghe, J. Schellenberg, et al., "District decision-making for health in low-income settings: a case study of the potential of public and private sector data in India and Ethiopia," Health Policy Plan, vol. 31 Suppl 2, pp. ii25-ii34, Sep 2016.

[88] S. Biliguo, "ACCESS TO COMPLEMENTARY SERVICES OF LIVELIHOOD EMPOWERMENT AGAINST POVERTY-(LEAP) SOCIAL PROTECTION INTERVENTION IN NADOWLI-KALEO DISTRICT, GHANA," 2020.

[89] M. Birara, T. Urgie, and A. F. Sium, "Locally advanced cervical cancer: Neoadjuvant chemotherapy plus radical surgery an alternative approach to chemo-radiation in a low-income setting: A descriptive study," vol. 19, p. e0310457, 2024.

[90] R. Bitran and B. Martorell, "AMFm: Reaching the poorest of the poor with effective malaria drugs," Washington DC: Resources for the Future, 2009.

[91] R. Bitrán and U. Giedion, "Waivers and exemptions for health services in developing countries. Final draft," World Bank, vol. 89, 2002.

[92] R. Bivins, J. Crane, K. Dodworth, A. Ercia, J. Hand, K. Logan, et al., Posters, protests, and prescriptions : Cultural histories of the National Health Service in Britain. Manchester: Manchester University Press, 2022.

[93] G. W. Bogale, H. Boer, and E. R. Seydel, "Reaching the hearts and minds of illiterate women in the Amhara highland of Ethiopia: Development and pre-testing of oral HIV/AIDS prevention messages," Sahara j, vol. 7, pp. 2-9, Jul 2010.

[94] Z. Bogetic, J. Bronfman, M. Jayawickrama, M. Klasnja, A. Mkrtchyan, M. Piatti, et al., "How Does Knowledge on Public Expenditures Integrate with the Design of Development Policy Operations?," Independent Evaluation Group (IEG), learning product, World Bank, 2015.

[95] M. T. Borde, "Geographical and Socioeconomic Inequalities in Maternal Mortality in Ethiopia," Int J Soc Determinants Health Health Serv, vol. 53, pp. 282-293, Jul 2023.

[96] I. Borowy, "Medical Aid, Repression, and International Relations: The East German Hospital at Metema," J Hist Med Allied Sci, vol. 71, pp. 64-92, Jan 2016.

[97] N. W. Boyce, H. Australia. Dept. of, and B. Family Services. Health Service Outcomes, Quality and outcome indicators for acute healthcare services : A research project for the National Hospital Outcomes Program (NHOP) Health Service Outcomes Branch. Canberra: Australian Government Publishing Service, 1997.

[98] N. Brikci, "The Failure of User Fees as a Health Financing Mechanism: An International Perspective," New Doctor, pp. 9-12, 2007.

[99] N. Brikci and M. Philips, "User fees or equity funds in low-income countries," The Lancet, vol. 369, pp. 10-11, 2007.

[100] A. S. Brink and S. F. Koch, "Did primary healthcare user fee abolition matter? Reconsidering South Africa's experience," Development Southern Africa, vol. 32, pp. 170-192, 2015.

[101] B. J. Burns, E. J. Costello, A. Angold, D. Tweed, D. Stangl, E. M. Farmer, et al., "Children's mental health service use across service sectors," Health affairs, vol. 14, pp. 147-159, 1995.

[102] C. M. CAFOD, "THE COST OF MEETING THE MDGs IN ZAMBIA," 2005.

[103] L. M. Calhoun, I. S. Speizer, D. Guilkey, and E. Bukusi, "The effect of the removal of user fees for delivery at public health facilities on institutional delivery in urban Kenya," Maternal and child health journal, vol. 22, pp. 409-418, 2018.

[104] A. Cambanis, M. A. Yassin, A. Ramsay, S. Bertel Squire, I. Arbide, and L. E. Cuevas, "Rural poverty and delayed presentation to tuberculosis services in Ethiopia," Trop Med Int Health, vol. 10, pp. 330-5, Apr 2005.

[105] D. Carr, "Improving the Health," 2004.

[106] F. Castro-Leal, J. Dayton, and L. Demery, "Public spending on health care in Africa: do the poor benefit?," Bulletin of the World health Organization, vol. 78, pp. 66-74, 2000.

[107] J. C. Chali, V. Nason, S. Nadakuditi, B. Kumar, and T. Kumar, Healthcare Paradigms: Cari Journals USA LLC, 2024.

[108] K. Chapman, Using social transfers to scale up equitable access to education and health services: Department for International Development London, 2006.

[109] J. Chapman and A. Brown, "Maternity units to the rescue," RCM Midwives, vol. 8, pp. 58-9, Feb 2005.

[110] M. E. Chauke, M. Ganga-Limando, T. A. Baykeda, W. D. Negash, T. B. Belachew, S. M. Fetene, et al., "Trends of socioeconomic and geographic inequalities in severe wasting among under-five children in Ethiopia from 2000 to 2019: using the WHO Health Equity Assessment Toolkit," BMJ Open, vol. 14, p. 948, Jan 10 2024.

[111] M. E. Chauke, M. Ganga-Limando, A. M. Mekonen, and M. G. Gebregziabher, "The effect of community based health insurance on catastrophic health expenditure in Northeast Ethiopia: A cross sectional study," Reprod Health, vol. 13, p. e0205972, 2018.

[112] M. Chernet, "Exploring The Facilitators of Community-Based Health Insurance Scheme Bankruptcy and its Magnitude in North Mecha District, West Gojjam Zone, Amhara, Ethiopia, 2021," 2022.

[113] M. Chersich, S. Luchters, D. Blaauw, F. Scorgie, E. Kern, A. Van den Heever, et al., "Safeguarding maternal and child health in South Africa by starting the Child Support Grant before birth: Design lessons from pregnancy support programmes in 27 countries," South African Medical Journal, vol. 106, pp. 1192-1210, 2016.

[114] A. Chikova, "An analysis of the effects of the political environment on the governance of orphans and vulnerable children by non-state actors in Mutare, Zimbabwe," University of Pretoria (South Africa), 2020.

[115] I. Chirisa, "Social protection amid increasing instability in Zimbabwe: Scope, institutions and policy options," Informal and Formal Social Protection Systems in Sub-Saharan Africa, pp. 121-154, 2013.

[116] N. Chukwu, "A blockchain-based framework and process guide for intelligent exchange and use of health information in low resource environments," 2024.

[117] J. Chuma, J. Musimbi, V. Okungu, C. Goodman, and C. Molyneux, "Reducing user fees for primary health care in Kenya: Policy on paper or policy in practice?," International journal for equity in health, vol. 8, pp. 1-10, 2009.

[118] B. Cielo, M. Santillan, and V. de Claro, "Effect of a case-capped, fee-for-service payment mechanism on accessibility and affordability of health care," Health Affairs Scholar, vol. 2, p. qxae004, 2024.

[119] D. Coady, B. J. Clements, and S. Gupta, "Health Financing Systems in East Asia and the Pacific: Early Successes and Current Challenges," in The Economics of Public Health Care Reform in Advanced and Emerging Economies, ed: International Monetary Fund, 2012.

[120] D. Coady, M. Grosh, and J. Hoddinott, "The targeting of transfers in developing countries: Review of experience and lessons," Social Safety Net Primer Series, World Bank, Washington DC, 2002.

[121] S. Çolakoğlu, "Turkey’s COVID-19 Diplomacy: International cooperation in the age of global health and economic crisis," VISITING SCHOLARS’OPINION PAPER, p. 123, 2020.

[122] A. Colantoni, L. Salvati, and R. Regassa, "Environmental enteropathy and its association with water sanitation and hygiene in slum areas of Jimma Town Ethiopia," Ambio, vol. 18, p. e0286866, 2023.

[123] D. Congo and B. Faso, "Health Accounts Impacted Policy in over 30 Countries Worldwide," 2018.

[124] J. A. Cook, G. Fitzgibbon, J. Burke-Miller, M. Williams, J.-B. Kim, C. A. Heflinger, et al., "A multi-site study of Medicaid-funded managed care versus fee-for-service plans' effects on mental health service utilization of children with severe emotional disturbance," The Journal of Behavioral Health Services & Research, vol. 31, pp. 384-402, 2004.

[125] D. L. Coppock, S. Desta, S. Tezera, and G. Gebru, "Capacity building helps pastoral women transform impoverished communities in Ethiopia," Science, vol. 334, pp. 1394-8, Dec 9 2011.

[126] A. L. Creese, "User charges for health care: a review of recent experience," Health policy and planning, vol. 6, pp. 309-319, 1991.

[127] E. Crystal, M. L. Laterveer, and M. M. Munga, "Equity Implications of Health Sector User Fees in Tanzania," Leusden: ETC Crystal, 2004.

[128] I. Cuellar Flores, L. Fernández Garzón, M. Ferreira González, M. Félix-Alcántara, I. de la Vega Rodríguez, B. Manzano Olivares, et al., "Variability in the practice of ambulatory Clinical Psychology in the Health Service of Madrid: Variabilidad en la práctica de la Psicología Clínica ambulatoria," Anuario de psicología (Barcelona, Spain), vol. 54, 2024.

[129] L. Curry, L. Taylor, P. G. Chen, and E. Bradley, "Experiences of leadership in health care in sub-Saharan Africa," Hum Resour Health, vol. 10, p. 33, Sep 13 2012.

[130] S. Damerow, "Maternal health in Sub-Saharan Africa: are national user fee waiver policies for intrapartum services the key to reducing maternal mortality? A quantitative cross-country comparison," Hochschule für Angewandte Wissenschaften Hamburg, 2023.

[131] W. V. Damme, L. V. Leemput, I. Por, W. Hardeman, and B. Meessen, "Out‐of‐pocket health expenditure and debt in poor households: evidence from Cambodia," Tropical Medicine & International Health, vol. 9, pp. 273-280, 2004.

[132] S. A. Damtew and S. Shiferaw, "Intimate Partner Violence during the Index Pregnancy and its correlates among a panel of pregnant women in Ethiopia, evidence from performance, and monitoring for action (PMA) 2021 cohort two baseline survey," vol. 24, p. 759, Nov 16 2024.

[133] H. Dao, H. Waters, and Q. Le, "User fees and health service utilization in Vietnam: how to protect the poor?," Public Health, vol. 122, pp. 1068-1078, 2008.

[134] E. Davidovich, E. Kooby, J. Shapira, and D. Ram, "Oral hygiene habits, dental home, and toothbrushing among immigrant and native low socioeconomic class populations," J Clin Pediatr Dent, vol. 37, pp. 341-4, Summer 2013.

[135] Z. Y. Debebe, A. Mebratie, R. Sparrow, M. Dekker, G. Alemu, and A. S. Bedi, "Impact of Ethiopia’s Community Based Health Insurance on household economic welfare," ISS Working Paper Series/General Series, vol. 590, pp. 1-27, 2014.

[136] A. Debie, R. B. Khatri, and Y. Assefa, "Contributions and challenges of healthcare financing towards universal health coverage in Ethiopia: a narrative evidence synthesis," BMC health services research, vol. 22, p. 866, 2022.

[137] J. T. de Jong, I. H. Komproe, M. Van Ommeren, M. El Masri, M. Araya, N. Khaled, et al., "Lifetime events and posttraumatic stress disorder in 4 postconflict settings," Jama, vol. 286, pp. 555-62, Aug 1 2001.

[138] C. M. Dean, A business health service: can businesses rely on professional specialist advice and assistance? Newcastle upon Tyne, UK: Cambridge Scholars Publishing, 2019.

[139] A. Defar, G. Molla, S. Abdella, M. Tessema, M. Ahmed, A. Tadele, et al., "Knowledge, practice and associated factors towards the prevention of COVID-19 among high-risk groups: A cross-sectional study in Addis Ababa, Ethiopia," PLoS One, vol. 16, p. e0248420, 2021.

[140] M. B. Degefa, B. T. Woldehanna, and A. D. Mebratie, "Effect of community-based health insurance on catastrophic health expenditure among chronic disease patients in Asella referral hospital, Southeast Ethiopia: a comparative cross-sectional study," BMC Health Serv Res, vol. 23, p. 188, Feb 23 2023.

[141] W. De La Haye and S. Alexis, "The impact of a no-user-fee policy on the quality of patient care/service delivery in Jamaica," West indian medical journal, vol. 61, p. 168, 2012.

[142] D. Demelash, "Willingness to Join and Pay for Social Health Insurance and Associated factors Among Civil Servants in Merawi Town, North West Ethiopia," 2021.

[143] B. Demissie and K. Gutema Negeri, "Effect of community-based health insurance on utilization of outpatient health care services in Southern Ethiopia: a comparative cross-sectional study," Risk management and healthcare policy, pp. 141-153, 2020.

[144] M. L. Dennis, "Pragmatic pluralism for health: Understanding the role of public financing and public-private engagement on use, quality, and equity in access to maternal health services in Kenya," London School of Hygiene & Tropical Medicine, 2020.

[145] M. L. Dennis, L. Benova, C. Goodman, E. Barasa, T. Abuya, and O. M. Campbell, "Examining user fee reductions in public primary healthcare facilities in Kenya, 1997–2012: effects on the use and content of antenatal care," International Journal for Equity in Health, vol. 19, pp. 1-13, 2020.

[146] S. Dercon and A. Sánchez, "Height in mid childhood and psychosocial competencies in late childhood: evidence from four developing countries," Econ Hum Biol, vol. 11, pp. 426-32, Dec 2013.

[147] A. Derseh, R. Sparrow, Z. Y. Debebe, G. Alemu, and A. S. Bedi, "Enrolment in Ethiopia’s community based health insurance scheme," ISS Working Paper Series/General Series, vol. 578, pp. 1-35, 2013.

[148] N. Deyessa, Y. Berhane, A. Alem, U. Hogberg, and G. Kullgren, "Depression among women in rural Ethiopia as related to socioeconomic factors: a community-based study on women in reproductive age groups," Scand J Public Health, vol. 36, pp. 589-97, Aug 2008.

[149] H. T. Dhufera, "Identifying Indigent Households for Subsidy and Exemption of Community Health Insurance Premium in Ethiopia: Considerations of Technical Approaches and Implementation Challenges," 2024.

[150] G.-C. Diego, P. Alberto, C.-M. Serafín, and P. M.-D. Pedro, "Impact of COVID-19 prevention measures on health service quality, perceived value and user satisfaction. A structural equation modelling (SEM) approach," Atención primaria, vol. 54, p. 102178, 2022.

[151] A. Dingle, "Equity of access to reproductive and maternal health services in Cambodia: equity trends, poverty targeting and demand-side financing," London School of Hygiene & Tropical Medicine, 2016.

[152] B. K. Doka, A. G. Worku, K. G. Negeri, and D. H. Kassa, "Assessment of data quality and associated factors in the routine health information system among health workers in public health institutions of Gofa Zone, Southern Ethiopia: A mixed methods study," Digital Health, vol. 10, p. 20552076241297227, 2024.

[153] M. Döll, "Making People Breathe: A Case Study of the Shanghai Social Assistance Programme Di Bao," 2012.

[154] D. B. Douglas, R. Waziry, E. P. McCarthy, A. W. Tadesse, M. D. Feyssa, M. Kawooya, et al., "Meeting the World Health Organization Maternal Antenatal Care Guidelines Is Associated with Improved Early and Middle Childhood Cognition in Ethiopia," J Pediatr, vol. 209, pp. 33-38.e1, Jun 2019.

[155] N. T. Douthit and H. K. Alemu, "Social determinants of health: poverty, national infrastructure and investment," BMJ Case Rep, vol. 2016, Jun 22 2016.

[156] D. B. Dutton, "Explaining the low use of health services by the poor: costs, attitudes, or delivery systems?," American sociological review, pp. 348-368, 1978.

[157] S. Dzakpasu, T. Powell-Jackson, and O. M. Campbell, "Impact of user fees on maternal health service utilization and related health outcomes: a systematic review," Health policy and planning, vol. 29, pp. 137-150, 2014.

[158] K. Eggleston, L. Ling, M. Qingyue, M. Lindelow, and A. Wagstaff, "Health service delivery in China: a literature review," Health economics, vol. 17, pp. 149-165, 2008.

[159] S. El Arifeen, A. Christou, L. Reichenbach, F. A. Osman, K. Azad, K. S. Islam, et al., "Community-based approaches and partnerships: innovations in health-service delivery in Bangladesh," The Lancet, vol. 382, pp. 2012-2026, 2013.

[160] M. El-Khoury, L. Hatt, and T. Gandaho, "User fee exemptions and equity in access to caesarean sections: an analysis of patient survey data in Mali," International Journal for Equity in Health, vol. 11, pp. 1-7, 2012.

[161] B. Enawgaw, M. Birhanie, B. Terefe, and F. Asrie, "Prevalence of Anemia and Iron Deficiency Among Pregnant Women Attending Antenatal Care Service at University of Gondar Hospital, Northwest Ethiopia," Clin Lab, vol. 65, Apr 1 2019.

[162] M. Endriyas, A. Kawza, A. Alano, and F. Lemango, "Quality of medical records in public health facilities: A case of Southern Ethiopia, resource limited setting," Health Informatics Journal, vol. 28, p. 14604582221112853, 2022.

[163] K. Engdawork and F. Sintayehu, "The State and Transformation of Female Wage Labour in Ethiopia: The Case of Textile/Garment Industries," The State and Transformation of Female Wage Labour in Ethiopia, p. 11, 2023.

[164] T. Ensor and J. Ronoh, "Effective financing of maternal health services: a review of the literature," Health policy, vol. 75, pp. 49-58, 2005.

[165] M. E. Estensen, R. M. Persson, S. Abebe, D. Mekonnen, B. Nega, A. Solholm, et al., "Fighting the good fight in Ethiopia," Open Heart, pp. 26-7, 1997.

[166] M. Etemadi and M. Hajizadeh, "User fee removal for the poor: a qualitative study to explore policies for social health assistance in Iran," BMC Health Services Research, vol. 22, p. 250, 2022.

[167] M. Ezra and G. E. Kiros, "Household vulnerability to food crisis and mortality in the drought-prone areas of northern Ethiopia," J Biosoc Sci, vol. 32, pp. 395-409, Jul 2000.

[168] E. L. Fink, A. von Saint Andre-von Arnim, R. Kumar, P. T. Wilson, T. Bacha, A. T. Aklilu, et al., "Traumatic Brain Injury and Infectious Encephalopathy in Children From Four Resource-Limited Settings in Africa," Pediatr Crit Care Med, vol. 19, pp. 649-657, Jul 2018.

[169] R. Fikre and Y. A. Fiche, "Provider's Knowledge and Availability of Emergency Obstetrics and Newborn Care Service in Case of Gedeo Zone, Southern Ethiopia 2017," Health Science Journal, vol. 12, 2018.

[170] R. Fikre, S. Gerards, W. Teklesilasie, and J. Gubbels, "Correlates of adverse outcomes of adolescent pregnancy in Sidama region, Ethiopia. An unmatched case-control study," Sex Reprod Healthc, vol. 41, p. 100986, Sep 2024.

[171] S. R. P. Franzen, C. Chandler, S. Siribaddana, J. Atashili, B. Angus, and T. Lang, "Strategies for developing sustainable health research capacity in low and middle-income countries: a prospective, qualitative study investigating the barriers and enablers to locally led clinical trial conduct in Ethiopia, Cameroon and Sri Lanka," BMJ Open, vol. 7, p. e017246, Oct 13 2017.

[172] T. Freeman, H. A. Gesesew, C. Bambra, E. R. J. Giugliani, J. Popay, D. Sanders, et al., "Why do some countries do better or worse in life expectancy relative to income? An analysis of Brazil, Ethiopia, and the United States of America," International journal for equity in health, vol. 19, pp. 1-19, 2020.

[173] L. M. Gadisa, "Decentralization and health sector reform: lessons from Ethiopia," Journal of Scientific Research and Biomedical Informatics, vol. 1, 2022.

[174] G. Y. Gan Jingwen, "Research and Effect Evaluation of Internal Performance Management Practice in Community Health Service Center in a District of Beijing," Zhongguo quanke yixue, vol. 27, pp. 1942-1949, 2024.

[175] K. Gandhi, E. Lim, J. Davis, and J. J. Chen, "Racial disparities in health service utilization among Medicare fee-for-service beneficiaries adjusting for multiple chronic conditions," Journal of Aging and Health, vol. 30, pp. 1224-1243, 2018.

[176] J. K. Ganle, M. Parker, R. Fitzpatrick, and E. Otupiri, "A qualitative study of health system barriers to accessibility and utilization of maternal and newborn healthcare services in Ghana after user-fee abolition," BMC pregnancy and childbirth, vol. 14, pp. 1-17, 2014.

[177] J. Gans, "Debates on US Immigration," ed: Sage Publications, 2012.

[178] T. Gebrehiwot and A. van der Veen, "Coping with food insecurity on a micro-scale: evidence from Ethiopian rural households," Ecol Food Nutr, vol. 53, pp. 214-40, 2014.

[179] A. Gebremariam, M. Assefa, A. Addissie, A. Worku, N. Dereje, A. Abreha, et al., "Delayed initiation of adjuvant chemotherapy among women with breast cancer in Addis Ababa, Ethiopia," Breast Cancer Res Treat, vol. 187, pp. 877-882, Jun 2021.

[180] T. Gebru and K. Lentiro, "The impact of community-based health insurance on health-related quality of life and associated factors in Ethiopia: a comparative cross-sectional study," PLoS One, vol. 16, p. 110, May 31 2018.

[181] A. Geleto, C. Chojenta, A. Musa, and D. Loxton, "Barriers to access and utilization of emergency obstetric care at health facilities in sub-Saharan Africa: a systematic review of literature," Systematic reviews, vol. 7, pp. 1-14, 2018.

[182] E. GENENE, "ASSESMENT OF PRIVATE WING PRACTICE IN ALL AFRICAN LEPROSY REHABILITATION CENTER," 2018.

[183] K. T. Gesese, "Migration and socio-demographic determinants of women’s reproductive health services utilization in North Gondar, Ethiopia," University of Leicester, 2015.

[184] E. T. Geta, A. Wakjira Bidika, and B. Etana, "Does community-based health insurance reduce disparities in modern health service utilization among households in Ethiopia? A community-based comparative cross-sectional study," Front Public Health, vol. 10, p. 1021660, 2022.

[185] N. Getachew, H. Shigut, G. Jeldu Edessa, and E. A. Yesuf, "Catastrophic health expenditure and associated factors among households of non community based health insurance districts, Ilubabor zone, Oromia regional state, southwest Ethiopia," International Journal for Equity in Health, vol. 22, p. 40, 2023.

[186] T. Getahun, L. Teklesilassie, M. Habtemichael, Y. Abebe, and H. Getahun, ""Magnitude of community-based health insurance utilization and associated factors in Bassona Worena District, North Shoa Zone, Ethiopia: a community-based cross-sectional study"," BMC Health Serv Res, vol. 22, p. 1405, Nov 24 2022.

[187] Z. Getaneh, M. Melku, M. Geta, T. Melak, and M. T. Hunegnaw, "Prevalence and determinants of stunting and wasting among public primary school children in Gondar town, northwest, Ethiopia," BMC Pediatr, vol. 19, p. 207, Jun 25 2019.

[188] Y. Getiye and M. Fantahun, "Factors associated with perinatal mortality among public health deliveries in Addis Ababa, Ethiopia, an unmatched case control study," BMC Pregnancy Childbirth, vol. 17, p. 245, Jul 26 2017.

[189] L. Gilson and D. McIntyre, "Removing user fees for primary care in Africa: the need for careful action," Bmj, vol. 331, pp. 762-765, 2005.

[190] B. Girma, "Assessment of Fee Waiver Health Care Implementation Status in the New Health Care Financing Strategy in Bahir Dar, North West Ethiopia," UNIVERSITY OF GONDAR, 2010.

[191] L. Gilson, S. Russell, and K. Buse, "The political economy of user fees with targeting: developing equitable health financing policy," Journal of international Development, vol. 7, pp. 369-401, 1995.

[192] F. Girmaw, E. Adane, A. T. Kassaw, G. Ashagrie, and T. Baye, "Willingness to Pay for Social Health Insurance Among Health Care Professionals in North Wollo Zone, Amhara Region, Ethiopia: Mixed Method Study," ClinicoEconomics and Outcomes Research, pp. 593-606, 2023.

[193] A. M. Girmay and M. T. Reta, "Community‐based health insurance service utilization and associated factors in Addis Ababa, Ethiopia," Public Health Challenges, vol. 1, p. e18, 2022.

[194] J. L. Gjerde, G. Rortveit, M. Muleta, and A. Blystad, "Silently waiting to heal: experiences among women living with urinary incontinence in northwest Ethiopia," Int Urogynecol J, vol. 24, pp. 953-8, Jun 2013.

[195] S. M. Goodwin and G. F. Anderson, "Effect of cost-sharing reductions on preventive service use among Medicare fee-for-service beneficiaries," Medicare & medicaid research review, vol. 2, p. 002.01. a03, 2012.

[196] G. Gordon-Strachan, W. Bailey, A. Henry-Lee, J. Barnett, S. Lalta, and D. Alleyne, "The impact of user fees for Preventive Health Care—Jamaica," Social and Economic Studies, pp. 123-152, 2010.

[197] G. Gotsadze, S. Bennett, K. Ranson, and D. Gzirishvili, "Health care-seeking behaviour and out-of-pocket payments in Tbilisi, Georgia," Health policy and planning, vol. 20, pp. 232-242, 2005.

[198] J. Goudge, L. Gilson, S. Russell, T. Gumede, and A. Mills, "Affordability, availability and acceptability barriers to health care for the chronically ill: longitudinal case studies from South Africa," BMC health services research, vol. 9, pp. 1-18, 2009.

[199] M. Grosh, C. P. R. Alas, U. Zafar, M. Wai-Poi, E. Tesliuc, and P. Leite, "Unpacking the empirics of targeting in low-and middle-income countries," Revisiting Targeting in Social Assistance: A New Look at Old Dilemmas, 2022.

[200] M. Grosh, C. Del Ninno, E. Tesliuc, and A. Ouerghi, For protection and promotion: The design and implementation of effective safety nets: World Bank Publications, 2008.

[201] A. Guda, "Challenges of Healthcare Financing: Economic and Welfare Effects of User Fees in Urban Ethiopia," MSc Thesis, Addis Ababa University, School of Economics, 2007.

[202] F. T. Gulta, T. Mulugeta, B. Wogayehu, and M. Mensa, "Patient satisfaction with pharmacy services among users and non users of community based health insurance scheme at public health facilities in Gamo Zone, South Ethiopia: a comparative cross sectional study," Journal of Pharmaceutical Health Care and Sciences, vol. 10, 2024.

[203] A. Guo, "User fees in primary healthcare in Sub-Saharan Africa: A study of the effects and legacy of World Bank neoliberal health policy," 2012.

[204] E. Gurmu and R. Mace, "Fertility decline driven by poverty: the case of Addis Ababa, Ethiopia," J Biosoc Sci, vol. 40, pp. 339-58, May 2008.

[205] A. Guta, B. Amsalu, T. Weldamanuel, A. Sema, L. Abera, B. S. Demissie, et al., "Utilization of modern contraceptives and associated factors among street women in Dire Dawa, Eastern Ethiopia: a mixed study," Reprod Health, vol. 18, p. 212, Oct 26 2021.

[206] D. R. Gwatkin, "Paying for health care: moving beyond the user-fee debate," The Lancet, vol. 380, pp. 88-90, 2012.

[207] C. Hadley, E. G. Stevenson, Y. Tadesse, and T. Belachew, "Rapidly rising food prices and the experience of food insecurity in urban Ethiopia: impacts on health and well-being," Soc Sci Med, vol. 75, pp. 2412-9, Dec 2012.

[208] J. Hagen-Zanker and R. Holmes, "Social protection in Nigeria," Synthesis report). London, UK: Overseas Development Institute, 2012.

[209] S. Hailegebreal, T. Melis, D. Haile, and N. Beyene, "Alcohol control policy in Ethiopia and implications for public health," PLoS One, vol. 40, pp. 423-435, Dec 2019.

[210] M. Hailemariam, A. Fekadu, M. Selamu, G. Medhin, M. Prince, and C. Hanlon, "Equitable access to integrated primary mental healthcare for people with severe mental disorders in Ethiopia: a formative study," Int J Equity Health, vol. 15, p. 121, Jul 26 2016.

[211] Y. Hailemichael, C. Hanlon, K. Tirfessa, S. Docrat, A. Alem, G. Medhin, et al., "Catastrophic health expenditure and impoverishment in households of persons with depression: a cross-sectional, comparative study in rural Ethiopia," BMC public health, vol. 19, pp. 1-13, 2019.

[212] Y. Hailemichael, C. Hanlon, K. Tirfessa, S. Docrat, A. Alem, G. Medhin, et al., "Mental health problems and socioeconomic disadvantage: a controlled household study in rural Ethiopia," BMJ Open, vol. 18, p. 121, Jul 31 2019.

[213] A. Hailu, R. Gebreyes, and O. F. Norheim, "Equity in public health spending in Ethiopia: a benefit incidence analysis," Health Policy and Planning, vol. 36, pp. i4-i13, 2021.

[214] A. G. Hailu and Z. Y. Amare, "Impact of productive safety net program on food security of beneficiary households in western Ethiopia: A matching estimator approach," PLoS One, vol. 17, p. e0260817, 2022.

[215] K. Hailu, Z. A. Alemu, and M. Adane, "Barriers to cleaning of shared latrines in slums of Addis Ababa, Ethiopia," vol. 17, p. e0263363, 2022.

[216] M. Hammad, F. Bacil, and F. V. Soares, "Next practices: Innovations in the COVID-19 social protection responses and beyond," Research Report2021.

[217] K. Hamren, H. S. Chungkham, and M. Hyde, "Religion, spirituality, social support and quality of life: measurement and predictors CASP-12(v2) amongst older Ethiopians living in Addis Ababa," Aging Ment Health, vol. 19, pp. 610-21, Jul 2015.

[218] Y. A. Hamza, J. Schellenberg, Z. Hill, and E. M. Bayked, "The impact of community-based health insurance on universal health coverage in Ethiopia: a systematic review and meta-analysis," BMC Med Res Methodol, vol. 16, p. 2189764, Dec 31 2023.

[219] P. Hangoma, "Social Protection, Health Risk, and Household Welfare in Zambia," 2017.

[220] C. Hanlon, A. Alem, G. Medhin, T. Shibre, D. A. Ejigu, H. Negussie, et al., "Task sharing for the care of severe mental disorders in a low-income country (TaSCS): study protocol for a randomised, controlled, non-inferiority trial," Trials, vol. 17, p. 76, Feb 11 2016.

[221] W. Hardeman, W. Van Damme, M. Van Pelt, I. Por, H. Kimvan, and B. Meessen, "Access to health care for all? User fees plus a Health Equity Fund in Sotnikum, Cambodia," Health policy and planning, vol. 19, pp. 22-32, 2004.

[222] B. Harris, J. Goudge, J. E. Ataguba, D. McIntyre, N. Nxumalo, S. Jikwana, et al., "Inequities in access to health care in South Africa," Journal of public health policy, vol. 32, pp. S102-S123, 2011.

[223] S. W. Hauge and H. Dalen, "Short-term outcome after open-heart surgery for severe chronic rheumatic heart disease in a low-income country, with comparison with an historical control group: an observational study," vol. 8, Aug 2021.

[224] J. Henriques, "Bearing the brunt of poverty," Nurs Stand, vol. 10, pp. 25-7, May 1 1996.

[225] E. Hensley, "The Refugee Economy in Jordan," 2020.

[226] R. Hill, G. Inchauste, N. Lustig, E. Tsehaye, and T. Woldehanna, "A fiscal incidence analysis for Ethiopia," The Distributional Impact of Fiscal Policy: Experience from Developing Countries, 2017.

[227] K. Hirvonen, A. Bossuyt, and R. Pigois, "Complementarities between social protection and health sector policies: Evidence from the productive safety net program in Ethiopia," International Food Policy Research Institute (IFPRI)2017.

[228] V. K. Hjellbakk, H. Hailemariam, F. Reta, and I. M. S. Engebretsen, "Diet and nutritional status among hospitalised children in Hawassa, Southern Ethiopia," BMC Pediatr, vol. 22, p. 57, Jan 21 2022.

[229] C. Holmemo, P. Acosta, T. George, R. J. Palacios, J. Pinxten, S. Sen, et al., "Investing in People: Social Protection for Indonesia's 2045 Vision," ed: World Bank, 2020.

[230] R. Holmes, B. Akinrimisi, J. Morgan, and R. Buck, "Social protection in Nigeria," Mapping programmes and their effectiveness. Overseas Development Institution (ODI), 2012.

[231] Z. Z. Huang Jinling, "The Logic and Trend of Urban Community Health Service Policies in China," Zhongguo quanke yixue, vol. 26, pp. 4239-4245, 2023.

[232] S. Huffman, "Women, work and pregnancy outcome," Mothers Child, vol. 7, pp. 1-3, 1988.

[233] S. Hurlburt, T. P. Hagen, M. Berhane, G. Abraham, A. Adamu, G. Tsega, et al., "Assessing medical impoverishment and associated factors in health care in Ethiopia," Inquiry, vol. 20, p. 7, Mar 30 2020.

[234] M. Hussien, M. Azage, and N. B. Bayou, "Financial viability of a community-based health insurance scheme in two districts of northeast Ethiopia: a mixed methods study," BMC Health Services Research, vol. 22, p. 1072, 2022.

[235] G. Hutton, "Charting the path to the World Bank’s “No blanket policy on user fees”: a look over the past 25 years at the shifting support for user fees in health and education, and reflections on the future," London, DFID Health Systems Resource Centre, 2004.

[236] G. Hutton, "Is the jury still out on the impact of user fees in Africa? A review of the evidence from selected countries on user fees and determinants of health service utilisation," East African medical journal, vol. 81, 2004.

[237] B. Jacobs and A. Price, "A comparative study of the effectiveness of pre-identification and passive identification for hospital fee waivers at a rural Cambodian hospital," Health and social protection: experiences from Cambodia, China and Lao PDF, 2008.

[238] B. Jacobs and N. Price, "The impact of the introduction of user fees at a district hospital in Cambodia," Health Policy and Planning, vol. 19, pp. 310-321, 2004.

[239] E. Jacobs, M. P. Bertone, J. Toonen, N. Akwataghibe, and S. Witter, "Performance-based financing, basic packages of health services and user-fee exemption mechanisms: an analysis of health-financing policy integration in three fragile and conflict-affected settings," Applied Health Economics and Health Policy, vol. 18, pp. 801-810, 2020.

[240] C. James, K. Hanson, B. McPake, D. Balabanova, D. Gwatkin, I. Hopwood, et al., "To Retain or Remove User Fees? Reflections on the Current Debate," ed: nd.

[241] C. Jehu-Appiah, G. Aryeetey, E. Spaan, T. De Hoop, I. Agyepong, and R. Baltussen, "Equity aspects of the National Health Insurance Scheme in Ghana: Who is enrolling, who is not and why?," Social science & medicine, vol. 72, pp. 157-165, 2011.

[242] S. H. I. Jiahua, H. Mingmin, S. U. N. Yongmei, W. Jiacheng, G. U. O. Jie, Q. I. Yongming, et al., "Current health service demands of new residents in shanghai," Shanghai Yufang Yixue, vol. 34, pp. 806-811, 2022.

[243] Q. Jingyi, M. Jian, Y. Jiaqi, L. U. O. Bingxing, W. Junling, Y. A. N. Juntao, et al., "Progress in researches on disease burden and health service utilization of beta-thalassemia patients in China," Zhongguo gong gong wei sheng = China public health, vol. 39, pp. 1354-1358, 2023.

[244] S. S. Jiwani, G. Gatica-Domínguez, I. Crochemore-Silva, and A. Maïga, "Trends and inequalities in the nutritional status of adolescent girls and adult women in sub-Saharan Africa since 2000: a cross-sectional series study," vol. 5, Oct 2020.

[245] K. A. Johansson, K. B. Strand, A. Fekadu, and D. Chisholm, "Health Gains and Financial Protection Provided by the Ethiopian Mental Health Strategy: an Extended Cost-Effectiveness Analysis," Health Policy Plan, vol. 32, pp. 376-383, Apr 1 2017.

[246] F. A. Johnson, F. Frempong-Ainguah, and S. S. Padmadas, "Two decades of maternity care fee exemption policies in Ghana: have they benefited the poor?," Health policy and planning, vol. 31, pp. 46-55, 2016.

[247] N. Jones and K. Pincock, "Intersecting inequalities, gender and adolescent health in Ethiopia," vol. 19, p. 97, Jun 15 2020.

[248] A. Karan, W. Yip, and A. Mahal, "Extending health insurance to the poor in India: An impact evaluation of Rashtriya Swasthya Bima Yojana on out of pocket spending for healthcare," Social Science & Medicine, vol. 181, pp. 83-92, 2017.

[249] A. M. Karim, A. Tamire, A. A. Medhanyie, and W. Betemariam, "Changes in equity of maternal, newborn, and child health care practices in 115 districts of rural Ethiopia: implications for the health extension program," BMC Pregnancy Childbirth, vol. 15, p. 238, Oct 5 2015.

[250] A. M. Kassa, "In Ethiopia's Kutaber district, does community-based health insurance protect households from catastrophic health-care costs? A community- based comparative cross-sectional study," PLoS One, vol. 18, p. e0281476, 2023.

[251] B. A. Kassa, F. T. Fantaye, A. Z. Armdie, S. Y. Berhe, D. G. Nerisho, A. Amogne, et al., "Opportunities and barriers to implementing antibiotic stewardship in low and middle-income countries: Lessons from a mixed-methods study in a tertiary care hospital in Ethiopia," BMC Pregnancy Childbirth, vol. 13, p. e0208447, 2018.

[252] M. Kifle, F. C. Payton, V. Mbarika, and P. Meso, "Transfer and adoption of advanced information technology solutions in resource-poor environments: the case of telemedicine systems adoption in Ethiopia," Telemed J E Health, vol. 16, pp. 327-43, Apr 2010.

[253] S. Khun and L. Manderson, "Poverty, user fees and ability to pay for health care for children with suspected dengue in rural Cambodia," International journal for equity in health, vol. 7, pp. 1-8, 2008.

[254] D. Kiima and R. Jenkins, "Mental health policy in Kenya-an integrated approach to scaling up equitable care for poor populations," International Journal of Mental Health Systems, vol. 4, pp. 1-8, 2010.

[255] M. Kiros, E. Dessie, A. Jbaily, M. T. Tolla, K. A. Johansson, O. F. Norheim, et al., "The burden of household out-of-pocket health expenditures in Ethiopia: estimates from a nationally representative survey (2015-16)," Health Policy Plan, vol. 35, pp. 1003-1010, Oct 1 2020.

[256] S. F. Koch, "User fee abolition and the demand for public health care," South African Journal of Economics, vol. 85, pp. 242-258, 2017.

[257] C. Korachais, P. Ir, E. Macouillard, and B. Meessen, "The impact of reimbursed user fee exemption of health centre outpatient consultations for the poor in pluralistic health systems: lessons from a quasi-experiment in two rural health districts in Cambodia," Health policy and planning, vol. 34, pp. 740-751, 2019.

[258] M. E. Kruk, G. Mbaruku, P. C. Rockers, and S. Galea, "User fee exemptions are not enough: out‐of‐pocket payments for ‘free’delivery services in rural Tanzania," Tropical medicine & international health, vol. 13, pp. 1442-1451, 2008.

[259] M. J. Kumsa, "Lack of pocket money impacts Ethiopian undergraduate health science students learning activities," BMJ Glob Health, vol. 15, p. e0243634, 2020.

[260] A. D. Koon, J. Wright, L. Ageze, J. Charles, and J. Holtz, "Aligning priorities in Ethiopian health finance: How do the essential health services package and health benefit plans compare?," The International Journal of Health Planning and Management, vol. 37, pp. 417-428, 2022.

[261] J. Kurji, B. Talbot, G. Bulcha, K. H. Bedru, S. Morankar, L. A. Gebretsadik, et al., "Uncovering spatial variation in maternal healthcare service use at subnational level in Jimma Zone, Ethiopia," BMC Health Serv Res, vol. 20, p. 703, Jul 31 2020.

[262] G. M. La Forgia and C. C. Griffin, Cost recovery in public hospitals in Belize: Health Financing and Sustainability Project, Abt Associates, 1992.

[263] R. Lambin and M. Nyyssölä, Two decades of Tanzanian health policy: Examining policy developments and opportunities through a gender lens: WIDER Working Paper, 2022.

[264] J. C. LANGENBRUNNER and A. TANDON, "Health financing systems in East Asia and the Pacific: early successes and current challenges," The Economics of Public Health Care Reform in Advanced and Emerging Economies, p. 133, 2012.

[265] S. Laokri, O. Weil, K. M. Drabo, S. M. Dembelé, B. Kafando, and B. Dujardin, "Removal of user fees no guarantee of universal health coverage: observations from Burkina Faso," Bulletin of the World Health Organization, vol. 91, pp. 277-282, 2013.

[266] M. Lapointe, "Urbanization and human-nature relationships: a comparison of urban and rural dwellers' perceptions of ecosystem services in the Solomon Islands," James Cook University, 2020.

[267] A. C. Lee, J. E. Lawn, S. Cousens, V. Kumar, D. Osrin, Z. A. Bhutta, et al., "Linking families and facilities for care at birth: what works to avert intrapartum-related deaths?," International Journal of Gynecology & Obstetrics, vol. 107, pp. S65-S88, 2009.

[268] L. D. Lekashingo, "Exploring the effects of user fees, quality of care and utilization of health services on enrolment in community health fund, Bagamoyo district, Tanzania," Muhimbili University of Health and Allied Sciences, 2012.

[269] C. Leighton, "22 Policy Questions About Health Care Financing in Africa," Report Submitted to USAID. <http://www>. dec. org/pdf_docs/PNABW870. pdf, accessed January, vol. 5, p. 2005, 1995.

[270] B. N. Lemu, T. M. Nguse, G. Shibre, B. Zegeye, D. Idriss-Wheeler, and S. Yaya, "Inequalities in measles immunization coverage in Ethiopia: a cross-sectional analysis of demographic and health surveys 2000-2016," PLoS One, vol. 20, p. 481, Jul 7 2020.

[271] T. Leone, V. Cetorelli, S. Neal, and Z. Matthews, "Financial accessibility and user fee reforms for maternal healthcare in five sub-Saharan countries: a quasi-experimental analysis," BMJ open, vol. 6, p. e009692, 2016.

[272] M. Lewis, "Informal payments and the financing of health care in developing and transition countries," Health Affairs, vol. 26, pp. 984-997, 2007.

[273] Y. Li, Q. Wu, L. Xu, D. Legge, Y. Hao, L. Gao, et al., "Factors affecting catastrophic health expenditure and impoverishment from medical expenses in China: policy implications of universal health insurance," Bulletin of the World Health Organization, vol. 90, pp. 664-671, 2012.

[274] C. Liang, H. Wang, and L. Tang, "Framework and Application Strategy of Smart Proactive Health Service," Zhongguo gong cheng ke xue (Online), vol. 25, pp. 30-42, 2023.

[275] M. Lindelöw and A. Wagstaff, Health facility surveys: an introduction vol. 2953: World Bank Publications, 2003.

[276] D. P. Lindstrom and B. Berhanu, "The impact of war, famine, and economic decline on marital fertility in Ethiopia," Demography, vol. 36, pp. 247-61, May 1999.

[277] E. Linnander, Z. McNatt, H. Sipsma, D. Tatek, Y. Abebe, A. Endeshaw, et al., "Use of a national collaborative to improve hospital quality in a low-income setting," Int Health, vol. 8, pp. 148-53, Mar 2016.

[278] J. I. Litvack and C. Bodart, "User fees plus quality equals improved access to health care: results of a field experiment in Cameroon," Social science & medicine, vol. 37, pp. 369-383, 1993.

[279] M. Loewe, "Community effects of cash-for-work programmes in Jordan: supporting social cohesion, more equitable gender roles and local economic development in contexts of flight and migration," DIE Studies, vol. 102, 2020.

[280] E. A. Lothe and K. Heggen, "A study of resilience in young Ethiopian famine survivors," J Transcult Nurs, vol. 14, pp. 313-20, Oct 2003.

[281] G. Louw and A. Duvenhage, "Are the fees that the traditional health practitioner charges generally lower than that of the medical practitioner?," 2017.

[282] E. Lufumpa, L. Doos, and A. Lindenmeyer, "Barriers and facilitators to preventive interventions for the development of obstetric fistulas among women in sub-Saharan Africa: a systematic review," BMC Pregnancy Childbirth, vol. 18, p. 155, May 10 2018.

[283] H. Y. Lukman and A. T. Ramadan, "Critical appraisal of the law enforcement in abortion care in Ethiopia," East Afr Med J, vol. 80, pp. 581-4, Nov 2003.

[284] A. Maeda, E. Araujo, C. Cashin, J. Harris, N. Ikegami, and M. R. Reich, Universal health coverage for inclusive and sustainable development: a synthesis of 11 country case studies: World Bank Publications, 2014.

[285] S. S. Mahmood, "Micro Health Insurance in Bangladesh: Prospects and Challenges," PQDT-Global, 2015.

[286] T. Maina and E. Onguti, "Health Policy and Abolition of User Fees at Public Primary Healthcare Facilities," 2014.

[287] W. Malenbaum, "Health and economic expansion in poor lands," Int J Health Serv, vol. 3, pp. 161-76, Spring 1973.

[288] S. Mall, M. Hailemariam, M. Selamu, A. Fekadu, C. Lund, V. Patel, et al., "'Restoring the person's life': a qualitative study to inform development of care for people with severe mental disorders in rural Ethiopia," Epidemiol Psychiatr Sci, vol. 26, pp. 43-52, Feb 2017.

[289] B. Mamdani, "Alternatives to user fees for public health care," Indian J Med Ethics, vol. 4, pp. 138-40, 2007.

[290] A. Mamo, S. Asfaw, L. Abebe, J. Kurji, G. Kiros, M. Abera, et al., "Developing a measure of mental health service satisfaction for use in low income countries: a mixed methods study," Int J Equity Health, vol. 17, p. 183, Mar 9 2017.

[291] R. Marcus, P. Pereznieto, E. Cullen, and N. Jones, "Children and social protection in the Middle East and North Africa," A mapping exercise.(No. ODI Working Paper 335). London, UK, 2011.

[292] A. María Brannan and C. A. Heflinger, "Child behavioral health service use and caregiver strain: Comparison of managed care and fee-for-service Medicaid systems," Mental Health Services Research, vol. 7, pp. 197-211, 2005.

[293] Z. M. Marselian, La Maestra’s Circle of Care: Studying the Impact of an Integrated Service Model on the Health and Well-Being of Vulnerable Populations: University of San Diego, 2020.

[294] M. Martin and M. Stulgaitis, "Refugees’ access to higher education in their host countries: Overcoming the ‘super-disadvantage.’," International Institute for Educational Planning(UNESCO), 2022.

[295] M. Martin Curran and D. G. Smith, "The impact of donkey ownership on the livelihoods of female peri-urban dwellers in Ethiopia," Trop Anim Health Prod, vol. 37 Suppl 1, pp. 67-86, Nov 2005.

[296] D. M. Marye, D. Debalkie Atnafu, M. Belayneh, and A. Takele Alemu, "User Fee Exemption Policy Significantly Improved Adherence to Maternal Health Service Utilization in Bahir Dar City, Northwest Ethiopia: A Comparative Cross-Sectional Study," ClinicoEconomics and Outcomes Research, pp. 775-785, 2023.

[297] M. Masinjila, "Policy mapping: women’s economic empowerment in Kenya," 2020.

[298] F. Masiye, B. M. Chitah, P. Chanda, and F. Simeo, "Removal of user fees at primary health care facilities in Zambia: a study of the effects on utilisation and quality of care," Regional Network for Equity in Health in East and Southern Africa Discussion Paper, vol. 57, 2008.

[299] F. Masiye, B. M. Chitah, and D. McIntyre, "From targeted exemptions to user fee abolition in health care: experience from rural Zambia," Social science & medicine, vol. 71, pp. 743-750, 2010.

[300] K. C. Maweu, A. Riechi, G. Kiranga, and M. Samuel, "Assessment of the Constituency Development Fund in Enhancing KCPE Performance of Public Primary Schools in Kenya: A Case of Mwala Constituency," 2011.

[301] J. MBINDANDONYI, "UTILIZATION OF FREE SKILLED BIRTH ATTENDANCE SERVICES AMONG WOMEN OF REPRODUCTIVE AGE IN PUBLIC HEALTH FACILITIES IN KITUI COUNTY, KENYA," KENYATTA UNIVERSITY, 2021.

[302] A. McCord, "Community-based targeting in the Social Protection sector," ed: Overseas Development Institute London, UK, 2013.

[303] D. McIntyre, A. G. Obse, E. W. Barasa, and J. E. Ataguba, "Challenges in financing universal health coverage in sub-Saharan Africa," in Oxford research encyclopedia of economics and finance, ed, 2018.

[304] R. McKay, Post-social prescriptions: Medical welfare in Mozambique: Stanford University, 2010.

[305] J. McKnight and D. B. Holt, "Designing the Expanded Programme on Immunisation (EPI) as a service: Prioritising patients over administrative logic," Glob Public Health, vol. 9, pp. 1152-66, 2014.

[306] D. McIntyre, M. Thiede, G. Dahlgren, and M. Whitehead, "What are the economic consequences for households of illness and of paying for health care in low-and middle-income country contexts?," Social science & medicine, vol. 62, pp. 858-865, 2006.

[307] B. McPake, "User charges for health services in developing countries: a review of the economic literature," Social science & medicine, vol. 36, pp. 1397-1405, 1993.

[308] B. McPake, A. Schmidt, E. Araujo, and C. Kirunga-Tashobya, "Freeing-up Healthcare: A guide to removing user fees," 2008.

[309] A. Mebratie, Essays on evaluating a community based health insurance scheme in rural Ethiopia, 2015.

[310] A. Mebratie, R. Sparrow, Z. Y. Debebe, D. Abebaw, G. Alemu, and A. S. Bedi, "The impact of Ethiopia’s pilot community based health insurance scheme on healthcare utilization and cost of care," ISS Working Paper Series/General Series, vol. 593, pp. 1-46, 2014.

[311] A. Mebratie, R. Sparrow, Z. Y. Debebe, G. Alemu, and A. S. Bedi, "Dropping out of Ethiopia’s Community Based Health Insurance scheme," ISS Working Paper Series/General Series, vol. 591, pp. 1-40, 2014.

[312] A. Mebratie, E. Van de Poel, Z. Y. Debebe, D. Abebaw, G. Alemu, and A. S. Bedi, "Self-reported health care seeking behavior in rural Ethiopia: evidence from clinical vignettes," ISS Working Paper Series/General Series, vol. 551, pp. 1-30, 2013.

[313] A. D. Mebratie, D. Shamebo, G. Alemu, Z. Shigute, and A. S. Bedi, "Willingness of urban formal sector workers to support a community-based health insurance scheme in Ethiopia," medRxiv, p. 2024.11. 15.24317322, 2024.

[314] A. D. Mebratie, R. Sparrow, Z. Yilma, G. Alemu, and A. S. Bedi, "Enrollment in Ethiopia’s community-based health insurance scheme," World Development, vol. 74, pp. 58-76, 2015.

[315] B. Meessen, L. Gilson, and A. Tibouti, "User fee removal in low-income countries: sharing knowledge to support managed implementation," vol. 26, ed: Oxford University Press, 2011, pp. ii1-ii4.

[316] B. Meessen, D. Hercot, M. Noirhomme, V. Ridde, A. Tibouti, C. K. Tashobya, et al., "Removing user fees in the health sector: a review of policy processes in six sub-Saharan African countries," Health policy and planning, vol. 26, pp. ii16-ii29, 2011.

[317] B. Meessen, W. Van Damme, C. K. Tashobya, and A. Tibouti, "Poverty and user fees for public health care in low-income countries: lessons from Uganda and Cambodia," The Lancet, vol. 368, pp. 2253-2257, 2006.

[318] K. M. Mekasha, "Assessment of Budget Preparation and Utilization: Case of Addis Ababa City Administration Health Bureau," 2015.

[319] A. M. Mekonen, M. G. Gebregziabher, and A. S. Teferra, "The effect of community based health insurance on catastrophic health expenditure in Northeast Ethiopia: A cross sectional study," PloS one, vol. 13, p. e0205972, 2018.

[320] A. Mekonnen and N. Jones, Tackling child malnutrition in Ethiopia, 2005.

[321] G. B. Mekonnen and D. A. Gelayee, "Low Medication Knowledge and Adherence to Oral Chronic Medications among Patients Attending Community Pharmacies: A Cross-Sectional Study in a Low-Income Country," vol. 2020, p. 4392058, 2020.

[322] W. Mekonnen and W. Dechassa, "Inter-district and Wealth-related Inequalities in Maternal and Child Health Service Coverage and Child Mortality within Addis Ababa City," vol. 101, pp. 68-80, Nov 2024.

[323] Z. Melaku, M. Alemayehu, K. Oli, and G. Tizazu, "Pattern of admissions to the medical intensive care unit of Addis Ababa University Teaching Hospital," BMC Psychiatry, vol. 44, pp. 33-42, Jan 2006.

[324] T. P. Melchert, Foundations of Health Service Psychology : an evidence-based biopsychosocial approach, Second edition. ed. London: Elsevier, Academic Press, 2020.

[325] A. T. Melese, "Living Wage Report Non-Metropolitan Urban Ethiopia Ziway Region: Context Provided inthe Horticulture Sector," 2017.

[326] D. Y. Melesse, N. Tejedor-Garavito, K. Nilsen, T. Getachew, S. Mulu, N. Wondrad, et al., "Why some women who attend focused antenatal care fail to deliver in health facilities: a qualitative study of women's perspectives from slums of Addis Ababa, Ethiopia," J Urban Health, vol. 10, p. e039189, Dec 31 2020.

[327] S. T. Memirie, S. Verguet, O. F. Norheim, C. Levin, and K. A. Johansson, "Inequalities in utilization of maternal and child health services in Ethiopia: the role of primary health care," BMC Health Serv Res, vol. 16, p. 51, Feb 12 2016.

[328] Y. Merene, W. Lorenz, L. Opgenoorth, Y. Woldehawariat, and J. Schmidt, "Ground and tiger beetles (Coleoptera: Carabidae, Cicindelidae) of the Federal Democratic Republic of Ethiopia: a provisional faunistic checklist based on literature data," Zootaxa, vol. 5247, pp. 1-345, Feb 27 2023.

[329] D. MERSHA, "The Contribution of Heath Care Financing for Enhancing Drug Supply Capacity in Public Health Centers in Addis Ababa in the case of Bole Sub city," St. Mary's University, 2015.

[330] A. G. Mesele, A. Y. Birhanu, A. M. Shiferaw, and N. D. Baykemagn, "District health information system 2 data utilization among health professionals in Amara region private hospitals, Ethiopia," Digital Health, vol. 10, p. 20552076241283239, 2024.

[331] S. Miles and J. L. Malone, "Perspectives from Ethiopia regarding U.S. military humanitarian assistance: how to build a better medical civil action project (MEDCAP)," Mil Med, vol. 178, pp. 1349-52, Dec 2013.

[332] A. Mills, J. E. Ataguba, J. Akazili, J. Borghi, B. Garshong, S. Makawia, et al., "Equity in financing and use of health care in Ghana, South Africa, and Tanzania: implications for paths to universal coverage," The Lancet, vol. 380, pp. 126-133, 2012.

[333] K. H. Misgina, E. M. van der Beek, H. M. Boezen, A. M. Bezabih, and H. Groen, "Pre-conception and prenatal factors influencing gestational weight gain: a prospective study in Tigray region, northern Ethiopia," BMC Pregnancy Childbirth, vol. 21, p. 718, Oct 26 2021.

[334] S. K. Mojumdar, "Trends in Maternal Care Utilization in Urban India: A Temporal Analysis," Environment and Urbanization ASIA, vol. 6, pp. 212-224, 2015.

[335] M. Molla, I. Mitiku, A. Worku, and A. Yamin, "Impacts of maternal mortality on living children and families: A qualitative study from Butajira, Ethiopia," Reprod Health, vol. 12 Suppl 1, p. S6, May 6 2015.

[336] P. Moszynski, "Zambia scraps healthcare fees for poor rural people," BMJ, vol. 332, p. 813, 2006.

[337] Y. A. Moyehodie, S. M. Fenta, S. S. Mulugeta, S. B. Agegn, E. Yismaw, H. B. Biresaw, et al., "Factors associated with community based health insurance healthcare service utilization of households in South Gondar zone, Amhara, Ethiopia. A community-based cross-sectional study," Health Services Insights, vol. 15, p. 11786329221096065, 2022.

[338] T. Muche Ewunie, D. Sisay, and R. H. Kabthymer, "Diabetes mellitus and its association with central obesity, and overweight/obesity among adults in Ethiopia. A systematic review and meta-analysis," PLoS One, vol. 17, p. e0269877, 2022.

[339] J. O. Mugisha, E. J. Schatz, J. Negin, P. Mwaniki, P. Kowal, and J. Seeley, "Timing of most recent health care visit by older people living with and without HIV: findings from the SAGE well-being of older people study in Uganda," The International Journal of Aging and Human Development, vol. 85, pp. 18-32, 2017.

[340] W. K. Munene, W. Mwaura-Tenambergen, and L. Muiruri, "Implementation of User Fee Policy in Psychiatric Hospitals in Kenya: A Case of Mathari Hospital, Nairobi," Journal of Medicine, Nursing and Public health, vol. 4, pp. 38-51, 2024.

[341] V. Munishi, "Assessment of user fee system: implementation of exemption and waiver mechanisms in Tanzania: successes and challenges," 2010.

[342] A. M. Musau, "Choice Of Place Of Birth In The Era Of The Maternity Subsidy In Kenya," University of Nairobi, 2019.

[343] E. C. Mussa, T. Palermo, G. Angeles, M. Kibur, and F. Otchere, "Impact of community-based health insurance on health services utilisation among vulnerable households in Amhara region, Ethiopia," BMC Health Services Research, vol. 23, p. 55, 2023.

[344] M. H. Mustapher, "Impacts of National Health Insurance Fund revenue on quality health service delivery at Dodoma City in Tanzania," The Open University of Tanzania, 2023.

[345] D. Mwangi and M. Jonah, "Effect of the Free Maternity Programme on the Access and Outcomes of Maternal and Newborn Health (MNH) In the County of Kiambu," 2017.

[346] R. L. Naeye, N. Tafari, D. Judge, D. Gilmour, and C. Marboe, "Amniotic fluid infections in an African city," J Pediatr, vol. 90, pp. 965-70, Jun 1977.

[347] S. Negash, A. Yap, C. Q. Stephens, B. G. Nigussie, R. F. Tefera, E. Bryce, et al., "Correlation of Pediatric Surgical Infrastructure With Clinical and Economic Outcomes: A Cohort Study," J Surg Res, vol. 303, pp. 215-223, Nov 2024.

[348] N. J. Nagelkerke and S. J. De Vlas, The epidemiological impact of an HIV vaccine on the HIV/AIDS epidemic in Southern India vol. 2978: World Bank Publications, 2003.

[349] G. Nenko, "ASSESSMENT OF FREE HEALTH SERVICE PROVISION SYSTEM IN DILLA TOWN, SOUTH ETHIOPIA," 2017.

[350] G. A. NGWACHO, "Effects of hidden costs in free secondary education on transition and completion rates in public boarding schools in Kisii County, Kenya," Kabarak University, 2015.

[351] J. K. H. Nielsen, "Brain Drain in Ethiopia’s Health Sector: Perceptions of and experiences with medical brain drain among Ethiopian health workers," 2021.

[352] C. M. Nirmala Ravishankar, Patricia Hernández-Peña, and Ravindra Rannan-Eliya, "Health Financing Analysis for Countdown Case Studies," 2014.

[353] E. Normand, "OG-7-5 Sculpting Surgical Safety: The Transformative," Canadian Medical Education Journal, p. 216, 2024.

[354] A. Obse, D. Hailemariam, and C. Normand, "Knowledge of and preferences for health insurance among formal sector employees in Addis Ababa: a qualitative study," BMC health services research, vol. 15, pp. 1-11, 2015.

[355] A. G. Obse and J. E. Ataguba, "Assessing medical impoverishment and associated factors in health care in Ethiopia," BMC international health and human rights, vol. 20, pp. 1-9, 2020.

[356] A. G. Obse and J. E. Ataguba, "Assessing catastrophic out-of-pocket payments in Ethiopia’s health system," 2020.

[357] A. Obse, M. Ryan, S. Heidenreich, C. Normand, and D. Hailemariam, "Eliciting preferences for social health insurance in Ethiopia: a discrete choice experiment," Health Policy Plan, vol. 31, pp. 1423-1432, Dec 2016.

[358] D. L. Ocho, P. C. Struik, L. L. Price, E. Kelbessa, and K. Kolo, "Assessing the levels of food shortage using the traffic light metaphor by analyzing the gathering and consumption of wild food plants, crop parts and crop residues in Konso, Ethiopia," J Ethnobiol Ethnomed, vol. 8, p. 30, Aug 7 2012.

[359] O. Ogundele, "Inequalities in the use of reproductive health care in Sub-Saharan Africa: evidence from Ghana and Nigeria," 2020.

[360] N. OOMMAN, E. LULE, and R. I. C. D EBORAH VAZIRANI, "INEQUALITIES IN H EALTH, NUTRITION AND POPULATION," Health, 2003.

[361] N. Oomman, E. Lule, E. Vazirani, and R. Chhabra, "Inequalities in health and population," ed, 2007.

[362] K. H. Onarheim, M. M. Sisay, M. Gizaw, K. M. Moland, and I. Miljeteig, "What if the baby doesn't survive? Health-care decision making for ill newborns in Ethiopia," Biomed Res Int, vol. 195, pp. 123-130, Dec 2017.

[363] K. H. Onarheim, M. M. Sisay, M. Gizaw, K. M. Moland, O. F. Norheim, and I. Miljeteig, "Selling my sheep to pay for medicines - household priorities and coping strategies in a setting without universal health coverage," BMC Health Serv Res, vol. 18, p. 153, Mar 2 2018.

[364] W. H. Organization, Health, economic growth and poverty reduction: the report of Working Group I of the Commission on Macroeconomics and Health: World Health Organization, 2002.

[365] W. H. Organization, "Building on the user-fee experience: The African case," 2003.

[366] W. H. Organization, "Transformative accountability for adolescents: accountability for the health and human rights of women, children and adolescents in the 2030 agenda," 2017.

[367] B. Oyugi, Z. Audi-Poquillon, S. Kendall, and S. Peckham, "Examining the quality of care across the continuum of maternal care (antenatal, perinatal and postnatal care) under the expanded free maternity policy (Linda Mama Policy) in Kenya: a mixed-methods study," BMJ open, vol. 14, p. e082011, 2024.

[368] L. Pearson, M. Gandhi, K. Admasu, and E. B. Keyes, "User fees and maternity services in Ethiopia," International journal of gynecology & obstetrics, vol. 115, pp. 310-315, 2011.

[369] M. Pearson and C. Chandler, "Knowing antmicrobial resistance in practice: a multi-country qualitative study with human and animal healthcare professionals," Glob Health Action, vol. 12, p. 1599560, 2019.

[370] V. Percival, E. Richards, T. MacLean, and S. Theobald, "Health systems and gender in post-conflict contexts: building back better?," Conflict and Health, vol. 8, pp. 1-14, 2014.

[371] N. Pouw and K. Bender, "The poverty reduction effect of social protection: the pros and cons of a multidisciplinary approach," The European Journal of Development Research, vol. 34, pp. 2204-2223, 2022.

[372] D. A. Bradt and C. M. Drummond, "EPIDEMIC PREPAREDNESS AND RESPONSE," Reference Manual for Humanitarian Health Professionals, p. 221, 2016.

[373] W. Quentin, O. Abosede, J. Aka, P. Akweongo, K. Dinard, A. Ezeh, et al., "Inequalities in child mortality in ten major African cities," BMC Med, vol. 12, p. 95, Jun 6 2014.

[374] S. Quimbo, N. Wagner, J. Florentino, O. Solon, and J. Peabody, "Long-term effects of a randomized policy experiment on quality improvement in the Philippines," in Abstracts of the Third Global Symposium on Health Systems Research, 2014.

[375] M. H. Rad, T. Ejajo, L. T. Elilo, S. A. Nedamo, D. Sullamo, A. H. Hailegebireal, et al., "Disparities in satisfaction among insured and uninsured adult outpatient department service users in Southern Ethiopia, 2022: a comparative cross-sectional study," BMC Health Serv Res, vol. 24, p. 807, Jul 12 2024.

[376] M. Rakotomalala, B. B. Abera, J. Rakotoarisoa, D. Alemu, E. Hébrard, A. Pinel-Galzi, et al., "Complete Genome Sequences of Rice Yellow Mottle Virus Isolates from the Federal Democratic Republic of Ethiopia," Microbiol Resour Announc, vol. 8, Jul 25 2019.

[377] M. Radhika, "Knowledge and Practice of Auxiliary Nurse Midwives (ANMS) Regarding Reproductive and Child Health Services," Rajiv Gandhi University of Health Sciences (India), 2017.

[378] T. S. Ravindran and V. Govender, "Sexual and reproductive health services in universal health coverage: a review of recent evidence from low-and middle-income countries," Sexual and reproductive health matters, vol. 28, p. 1779632, 2020.

[379] H. E. Rosen, P. F. Lynam, C. Carr, V. Reis, J. Ricca, E. S. Bazant, et al., "Direct observation of respectful maternity care in five countries: a cross-sectional study of health facilities in East and Southern Africa," BMC Pregnancy Childbirth, vol. 15, p. 306, Nov 23 2015.

[380] A. Robinson, Sanitation Finance in Rural Cambodia: Water and Sanitation Program, 2012.

[381] E. Sado and T. Gedif, "Drug utilization at household level in Nekemte Town and surrounding rural areas, western Ethiopia: a cross-sectional study," Open access library Journal, vol. 1, p. 1, 2014.

[382] F. Samuels, C. Blake, and B. Akinrimisi, "HIV vulnerabilities and the potential for strengthening social protection responses in the context of HIV in Nigeria," London: ODI, 2012.

[383] H. Sanbata, A. Asfaw, and A. Kumie, "Association of biomass fuel use with acute respiratory infections among under- five children in a slum urban of Addis Ababa, Ethiopia," BMC Public Health, vol. 14, p. 1122, Oct 31 2014.

[384] N. A. Scott, "Orphans in Zambia: Program monitoring and evaluation practices and the association of external support with education status and psychosocial wellbeing," Boston University, 2013.

[385] M. C. A. Sdralevich, M. Sab, M. Y. Zouhar, and M. G. Albertin, "Subsidy reform in the Middle East and North Africa: Recent progress and challenges ahead," 2014.

[386] E. Seery, A. Marriott, K. Malouf Bous, and R. Shadwick, "From Catastrophe to Catalyst: Can the World Bank make COVID-19 a turning point for building universal and fair public healthcare systems?," 2020.

[387] M. Seid, A. Minyihun, G. Tilahun, A. Atnafu, and G. Amare, "Willingness to pay for cataract surgery and associated factors among cataract patients in Outreach Site, North West Ethiopia," Plos one, vol. 16, p. e0248618, 2021.

[388] E. Selenica, "Universities between the State and the Market: Development Policy, Commercialization and Liberalization in Higher Education," 2018.

[389] S. Semahagn, "Health Centers Pharmacy Service Reform Practice and Associated factors among Health Professionals in Central Gondar Zone, North West Ethiopia, 2022," 2023.

[390] A. Semahegn and B. Mengistie, "Domestic violence against women and associated factors in Ethiopia; systematic review," Reprod Health, vol. 12, p. 78, Aug 29 2015.

[391] M. Semrau, S. Evans-Lacko, A. Alem, J. L. Ayuso-Mateos, D. Chisholm, O. Gureje, et al., "Strengthening mental health systems in low- and middle-income countries: the Emerald programme," BMC Med, vol. 13, p. 79, Apr 10 2015.

[392] E. G. Sendo, "Women's perspectives on the measures that need to be taken to increase the use of health-care facility delivery service among slums women, Addis Ababa, Ethiopia: a qualitative study," BMC Infect Dis, vol. 18, p. 174, Aug 23 2021.

[393] A. Shahvisi, E. Meskele, and G. Davey, "A Human Right to Shoes? Establishing Rights and Duties in the Prevention and Treatment of Podoconiosis," BMC Health Serv Res, vol. 20, pp. 53-65, Jun 2018.

[394] S. A. Shega, S. Nieuwoudt, and B. Makinwa, "Exploring factors affecting Health Extension Workers motivation in selected urban, rural and pastoralist districts of Ethiopia," Accessible: Univeristy of the Witwatersrand, 2013.

[395] A. Shepherd, F. Kessy, K. Higgins, L. Scott, and E. Luvanda, "Addressing chronic poverty and vulnerability through social assistance in Tanzania: Assessing the options," Manchester: CPRC, 2011.

[396] Z. Shigute, A. D. Mebratie, and A. S. Bedi, "Social protection schemes in Ethiopia: the productive safety net program and the community based health insurance scheme," in Global Labour in Distress, Volume II: Earnings,(In) decent Work and Institutions, ed: Springer, 2023, pp. 559-565.

[397] Z. Shigute, A. S. Bedi, M. Rieger, and A. L. Wagner, "The use and significance of vaccination cards," PLoS One, vol. 15, pp. 2844-2846, 2019.

[398] T. Shimels, R. Asrat Kassu, and G. Bogale, "Magnitude and associated factors of poor medication adherence among diabetic and hypertensive patients visiting public health facilities in Ethiopia during the COVID-19 pandemic," vol. 16, p. e0249222, 2021.

[399] M. G. Shrime, S. Verguet, K. A. Johansson, D. Desalegn, D. T. Jamison, and M. E. Kruk, "Task-sharing or public finance for the expansion of surgical access in rural Ethiopia: an extended cost-effectiveness analysis," Health Policy Plan, vol. 31, pp. 706-16, Jul 2016.

[400] S. A. Spangler, D. Barry, and L. Sibley, "An evaluation of equitable access to a community-based maternal and newborn health program in rural Ethiopia," J Midwifery Womens Health, vol. 59 Suppl 1, pp. S101-9, Jan 2014.

[401] H. Spitzer and M. Mabeyo, In search of protection: Older people and their fight for survival in Tanzania: African Books Collective, 2011.

[402] L. Steurs, "European aid and health system strengthening: an analysis of donor approaches in the DRC, Ethiopia, Uganda, Mozambique and the global fund," Glob Health Action, vol. 12, p. 1614371, 2019.

[403] S. Sumit, "User fee Experience in Madhya Pradesh under Rogi Kalyan Samiti," SCTIMST, 2006.

[404] A. Sundaram, M. Vlassoff, A. Bankole, L. Remez, and Y. Gebrehiwot, "Benefits of meeting the contraceptive needs of Ethiopian women," Issues Brief (Alan Guttmacher Inst), pp. 1-8, Jul 2010.

[405] M. Tadesse, A. Defar, T. Getachew, K. Amenu, H. Teklie, E. Asfaw, et al., "Countdown to 2015: Ethiopia's progress towards reduction in under-five mortality: 2014 country case study," 2015.

[406] G. Taddesse, G. Getaneh, and A. Habte, "Spatial variation and predictors of missing birth preparedness and complication readiness (BPCR) messages in Ethiopia," PLoS One, vol. 18, p. e0295744, 2023.

[407] D. Tamiru, M. Duguma, T. Belachew, M. Hailemariam, A. Fekadu, M. Selamu, et al., "Developing a mental health care plan in a low resource setting: the theory of change approach," PLoS One, vol. 15, p. 429, Sep 28 2015.

[408] W. Tarekegn, S. Tsegaye, and Y. Berhane, "Skilled birth attendant utilization trends, determinant and inequality gaps in Ethiopia," BMC Womens Health, vol. 22, p. 466, Nov 22 2022.

[409] H. Tasic, N. Akseer, S. H. Gebreyesus, A. Ataullahjan, S. Brar, E. Confreda, et al., "Drivers of stunting reduction in Ethiopia: a country case study," PLoS One, vol. 112, pp. 875s-893s, Sep 14 2020.

[410] B. Taye, B. Alemayehu, A. Birhanu, K. Desta, S. Addisu, B. Petros, et al., "Podoconiosis and soil-transmitted helminths (STHs): double burden of neglected tropical diseases in Wolaita zone, rural Southern Ethiopia," PLoS Negl Trop Dis, vol. 7, p. e2128, 2013.

[411] M. Tedla, A. Malede, and Z. Berhan, "Prevalence and associated factors of malnutrition among under-five children living in slum areas of Bahir Dar Town, Ethiopia," AJOB Empir Bioeth, vol. 47, p. 176, 2024.

[412] A. S. Teferra and N. Bergen, "Perceptions and experiences related to health and health inequality among rural communities in Jimma Zone, Ethiopia: a rapid qualitative assessment," PLoS One, vol. 17, p. 84, Jun 18 2018.

[413] A. Tegegne and M. Legese, "The Urban Poor and Health Seeking Behavior: The Healthcare Seeking Behavior of the ‘Poorest of the Poor’in Addis Ababa," 2014.

[414] T. Tekelab, A. S. Melka, and D. Wirtu, "Predictors of modern contraceptive methods use among married women of reproductive age groups in Western Ethiopia: a community based cross-sectional study," BMC Womens Health, vol. 15, p. 52, Jul 17 2015.

[415] H. D. Teklehaimanot and A. Teklehaimanot, "Human resource development for a community-based health extension program: a case study from Ethiopia," Hum Resour Health, vol. 11, p. 39, Aug 20 2013.

[416] D. Temesgen, T. Tilahun, R. Oljira, and B. Etana, "Utilization of Maternal Health Services in Western Ethiopia: A Community-Based Cross-Sectional Study," Asian Research Journal of Gynaecology and Obstetrics, vol. 8, pp. 22-32, 2022.

[417] Y. TENAW, "ANALYSIS OF FACTORS INFLUENCING INDIVIDUAL’S WILLINGNESS TO PAY FOR THE COMPULSORY SOCIAL HEALTH INSURANCE SCHEME: THE CASE OF GOVERNMENT SCHOOL TEACHERS IN KOLFE KERANIYO SUBCITY," St. Mary's University, 2017.

[418] Z. Tenaw, R. Fikre, H. Gemeda, and A. Astatkie, "Determinants of maternity waiting home utilization in Sidama Zone, Southern Ethiopia: A cross-sectional study," vol. 17, p. e0264416, 2022.

[419] F. S. Teni, B. M. Gebresillassie, E. M. Birru, S. A. Belachew, Y. G. Tefera, B. L. Wubishet, et al., "Costs incurred by outpatients at a university hospital in northwestern Ethiopia: a cross-sectional study," BMC health services research, vol. 18, pp. 1-10, 2018.

[420] K. Tesfaye, E. Tesfaye, and M. Kassa, "Assessment of Free Health Care Provision System in North Gondar Gondar Town, Ethiopia, 2011," Assessment, vol. 1, 2018.

[421] Y. G. Teshome, "Maternal health in Ethiopia: Global and Local complexities," Arizona State University2017.

[422] P. Thomas, "Ending child poverty and securing child rights: the role of social protection," Addis Ababa, Ethiopia: Plan Ethiopia, 2005.

[423] A. Tibebe, G. Amarech, T. Melesse, and D. H. Mariam, "Examining out of pocket payments for maternal health in rural Ethiopia: paradox of free health care un-affordability," Ethiopian Journal of Health Development, vol. 26, pp. 251-257, 2012.

[424] Y. T. Tigabu, M. K. Aredo, and A. Ademe, "Rural household income mobility in Ethiopia: Dimensions, drivers and policy," PLoS One, vol. 18, p. e0284987, 2023.

[425] D. Tilahun, C. Hanlon, A. Fekadu, B. Tekola, Y. Baheretibeb, and R. A. Hoekstra, "Stigma, explanatory models and unmet needs of caregivers of children with developmental disorders in a low-income African country: a cross-sectional facility-based survey," BMC Health Serv Res, vol. 16, p. 152, Apr 27 2016.

[426] K. Tirfessa, C. Lund, G. Medhin, Y. Hailemichael, A. Fekadu, and C. Hanlon, "Food insecurity among people with severe mental disorder in a rural Ethiopian setting: a comparative, population-based study," Epidemiol Psychiatr Sci, vol. 28, pp. 397-407, Aug 2019.

[427] K. Tirfessa, C. Lund, G. Medhin, M. Selamu, R. Birhane, Y. Hailemichael, et al., "Impact of integrated mental health care on food insecurity of households of people with severe mental illness in a rural African district: a community-based, controlled before-after study," Trop Med Int Health, vol. 25, pp. 414-423, Apr 2020.

[428] A. Tiyou, T. Belachew, F. Alemseged, and S. Biadgilign, "Food insecurity and associated factors among HIV-infected individuals receiving highly active antiretroviral therapy in Jimma zone Southwest Ethiopia," Nutr J, vol. 11, p. 51, Jul 23 2012.

[429] B. A. Tjarks, "Maternal Mortality in the Developing World–Simple Solutions Aren’t Enough," 2017.

[430] M. T. Tolla, "Prevention and treatment of cardiovascular disease in Ethiopia saves more than lives: cost-effectiveness analysis, extended cost-effectiveness analysis, and financial risk protection," 2018.

[431] M. T. Tolla, O. F. Norheim, S. Verguet, A. Bekele, K. Amenu, S. G. Abdisa, et al., "Out-of-pocket expenditures for prevention and treatment of cardiovascular disease in general and specialised cardiac hospitals in Addis Ababa, Ethiopia: a cross-sectional cohort study," BMJ global health, vol. 2, p. e000280, 2017.

[432] K. Tomlin, D. Berhanu, M. Gautham, N. Umar, J. Schellenberg, D. Wickremasinghe, et al., "Assessing capacity of health facilities to provide routine maternal and newborn care in low-income settings: what proportions are ready to provide good-quality care, and what proportions of women receive it?," BMC Pregnancy Childbirth, vol. 20, p. 289, May 12 2020.

[433] J. Trabitzsch, M. Marquardt, S. Negash, W. Belay, Y. Abebe, E. Seife, et al., "Understanding referral of patients with cancer in rural Ethiopia: a qualitative study," BMC cancer, vol. 24, p. 553, 2024.

[434] Y. Tsega, G. Tsega, A. M. Mekonen, T. Birhane, E. Addisu, A. Getie, et al., "Insured-non-insured disparity of catastrophic health expenditure in Northwest Ethiopia: a multivariate decomposition analysis," Health Economics Review, vol. 14, p. 53, 2024.

[435] Y. Tsega, G. Tsega, G. Taddesse, and G. Getaneh, "Leaving no one behind in health: financial hardship to access health care in Ethiopia," Plos one, vol. 18, p. e0282561, 2023.

[436] D. A. Tuoyire, L. Baatiema, D. Dwomoh, and S. Bosomprah, "Healthcare utilization in Ghana: Insights from the 2017 Ghana Living Standard Survey," Plos one, vol. 19, p. e0306032, 2024.

[437] UNICEF, "Enhanced Protection for Children Affected by AIDS," 2007.

[438] USAID, "GUIDELINES FOR SUPPORTIVE SUPERVISION IN TH EH EA LTH SEC TOR," 2007.

[439] S. Verguet, S. T. Memirie, and O. F. Norheim, "Assessing the burden of medical impoverishment by cause: a systematic breakdown by disease in Ethiopia," BMC Med, vol. 14, p. 164, Oct 21 2016.

[440] S. Verguet, Z. D. Olson, J. B. Babigumira, D. Desalegn, K. A. Johansson, M. E. Kruk, et al., "Health gains and financial risk protection afforded by public financing of selected interventions in Ethiopia: an extended cost-effectiveness analysis," Lancet Glob Health, vol. 3, pp. e288-96, May 2015.

[441] J. P. Vogel, J. E. Moore, C. Timmings, S. Khan, D. N. Khan, A. Defar, et al., "Barriers, Facilitators and Priorities for Implementation of WHO Maternal and Perinatal Health Guidelines in Four Lower-Income Countries: A GREAT Network Research Activity," PLoS One, vol. 11, p. e0160020, 2016.

[442] T. Wakayo, S. J. Whiting, and T. Belachew, "Vitamin D Deficiency is Associated with Overweight and/or Obesity among Schoolchildren in Central Ethiopia: A Cross-Sectional Study," Nutrients, vol. 8, p. 190, Apr 1 2016.

[443] W. G. Wako and D. H. Kassa, "Institutional delivery service utilization and associated factors among women of reproductive age in the mobile pastoral community of the Liban District in Guji Zone, Oromia, Southern Ethiopia: a cross sectional study," BMC pregnancy and childbirth, vol. 17, pp. 1-10, 2017.

[444] H. Wang and G. Ramana, "Universal health coverage for inclusive and sustainable development," Country Summary Report for Ethiopia, 2014.

[445] H. Wang and N. Rosemberg, "Universal health coverage in low-income countries: Tanzania’s efforts to overcome barriers to equitable health service access," 2018.

[446] C. M. Weiner, A. Mathewos, A. Addissie, W. Ayele, A. Aynalem, T. Wondemagegnehu, et al., "Characteristics and follow-up of metastatic breast cancer in Ethiopia: A cohort study of 573 women," Breast, vol. 42, pp. 23-30, Dec 2018.

[447] J. Wellum, "Critical analysis of the Kenyan healthcare system and models for improvement," 2014.

[448] R. Wibaek, T. Girma, B. Admassu, M. Abera, A. Abdissa, Z. Geto, et al., "Higher Weight and Weight Gain after 4 Years of Age Rather than Weight at Birth Are Associated with Adiposity, Markers of Glucose Metabolism, and Blood Pressure in 5-Year-Old Ethiopian Children," J Nutr, vol. 149, pp. 1785-1796, Oct 1 2019.

[449] D. Wickremasinghe, I. E. Hashmi, J. Schellenberg, and B. I. Avan, "District decision-making for health in low-income settings: a systematic literature review," Health Policy Plan, vol. 31 Suppl 2, pp. ii12-ii24, Sep 2016.

[450] S. Wiggins, R. Calow, J. Feyertag, S. Levine, and A. Lowe, "Policy Interventions to Mitigate Negative Effects on Poverty, Agriculture and Food Security, from Disease Outbreaks and Other Crises," ed: Agriculture Policy Research in Africa (APRA), ODI: London, UK, 2020.

[451] C. Y. Willis and C. Leighton, "Protecting the poor under cost recovery: the role of means testing," Health policy and planning, vol. 10, pp. 241-256, 1995.

[452] T. Woldehanna, R. Gudisa, Y. Tafere, and A. Pankhurst, "Understanding Changes in the Lives of Poor Children," ed: Citeseer, 2011.

[453] M. Woldie, K. Yitbarek, and G. Dinsa, "Resource Mobilisation and allocation for primary health care: lessons from the Ethiopian health system," Lancet Global Health Commission on Financing Primary Health Care, 2022.

[454] T. M. Wondawek and M. M. Ali, "Delay in treatment seeking and associated factors among suspected pulmonary tuberculosis patients in public health facilities of Adama town, eastern Ethiopia," BMC Public Health, vol. 19, p. 1527, Nov 14 2019.

[455] A. Workicho, T. Belachew, G. T. Feyissa, B. Wondafrash, C. Lachat, R. Verstraeten, et al., "Household dietary diversity and Animal Source Food consumption in Ethiopia: evidence from the 2011 Welfare Monitoring Survey," BMC Public Health, vol. 16, p. 1192, Nov 25 2016.

[456] B. N. Worku, T. G. Abessa, M. Wondafrash, J. Lemmens, J. Valy, L. Bruckers, et al., "Effects of home-based play-assisted stimulation on developmental performances of children living in extreme poverty: a randomized single-blind controlled trial," BMC Pediatr, vol. 18, p. 29, Feb 5 2018.

[457] B. N. Worku, T. G. Abessa, M. Wondafrash, M. Vanvuchelen, L. Bruckers, P. Kolsteren, et al., "The relationship of undernutrition/psychosocial factors and developmental outcomes of children in extreme poverty in Ethiopia," BMC Pediatr, vol. 18, p. 45, Feb 9 2018.

[458] B. Yakob and B. P. Ncama, "Measuring health system responsiveness at facility level in Ethiopia: performance, correlates and implications," BMC Health Serv Res, vol. 17, p. 263, Apr 11 2017.

[459] Q. Xu, Resident participation and community organization in China: Impacts and consequences of China's urban community services: University of Denver, 2002.

[460] T. Yawkal, "Financial Hardship of Healthcare and Associated factors Among Households in Debretabor Town, Amhara, Northwest Ethiopia, 2022," 2022.

[461] T. Yeneabat, H. Adugna, T. Asmamaw, M. Wubetu, M. Admas, G. Hailu, et al., "Maternal dietary diversity and micronutrient adequacy during pregnancy and related factors in East Gojjam Zone, Northwest Ethiopia, 2016," BMC Pregnancy Childbirth, vol. 19, p. 173, May 15 2019.

[462] Z. Yilma, A. D. Mebratie, R. Sparrow, M. Dekker, G. Alemu, and A. S. Bedi, "Economic Consequences of Ill-Health in Rural Ethiopia," Health Syst Reform, vol. 7, p. e1885577, Jul 1 2021.
