# Supplementary material for: Health service utilization and associated factors among fee waiver beneficiaries in Ethiopia: Systematic review and meta-analysis
Source: PLoS One. 2025 Jun 11;20(6):e0326131. doi: 10.1371/journal.pone.0326131 (PMC12157077; doi:10.1371/journal.pone.0326131)
Supplement: Supplemental File 3 — (DOCX) [file pone.0326131.s003.docx]

# Quality appraisal results of included analytical cross-sectional studies in Ethiopia, Using Joanna Briggs Institute (JBI) quality appraisal checklist for systematic review and meta-analysis

| **Included articles** | **Criterion number** | | | | | | | | **Score** | **Risk of bias** |
| --- | --- | --- | --- | --- | --- | --- | --- | --- | --- | --- |
|  | 1 | 2 | 3 | 4 | 5 | 6 | 7 | 8 |  |  |
| Amare et al. [[1](#_ENREF_1)] | ✓ | ✓ | ✓ | ✓ | ✓ | ✓ | ✓ | ✓ | 8 | Low |
| Nigusie et al. [[2](#_ENREF_2)] | ✓ | ✓ | ✓ | ✓ | ✓ | ✓ | ✓ | ✓ | 8 | Low |
| Chote et al.[[3](#_ENREF_3)] | ✓ | ✓ | ✓ | ✓ | ✓ | ✓ | ✓ | ✓ | 8 | Low |
| Damte et al.[[4](#_ENREF_4)] | ✓ | ✓ | ✓ | ✓ | ✓ | ✓ | ✓ | ✓ | 8 | Low |
| Dessie[[5](#_ENREF_5)] | ✓ | ✓ | ✓ | x | ✓ | ✓ | x | ✓ | 6 | Low |
| Jemal et al. [[6](#_ENREF_6)] | ✓ | ✓ | ✓ | ✓ | ✓ | ✓ | x | ✓ | 7 | Low |
| Tesfaye[[7](#_ENREF_7)] | ✓ | ✓ | x | ✓ | ✓ | ✓ | x | ✓ | 6 | Low |

# References

[1] G. Amare, A. Gessesse, M. Kebede, and M. Yitayal, "Health Service Utilization Among Out-of-Pocket Payers and Fee-Wavier Users in Saesie Tsaeda-Emba District, Tigray Region, Northern Ethiopia: A Comparative Cross-Sectional Study," *Risk Management and Healthcare Policy,* vol. Volume 14, pp. 695-703, 2021.

[2] F. Amsalu, M. Asnakew Molla, A. Kidist, C. Muluken Genetu, and T. Nigusie, "Health services utilization and associated factors among fee waiver beneficiaries’ in Dessie city administration, Northeast Ethiopia: a cross-sectional study design," *BMC Health Services Research,* vol. 22, pp. 1-10, 2022.

[3] T. Chote, K. Tushune, K. Yitbarek, and M. Woldie, "The utilization of health services among poor households with user fee payment waiver certificate in Gamo Gofa zone, southern Ethiopia," *Divers Equality Health Care,* vol. 14, pp. 243-8, 2017.

[4] B. Damte Tegegn and K. G. Negeri, "Assessment of utilization of health care services and the associated factors among adult fee-waiver beneficiaries in Hawassa, southern Ethiopia: a community based cross sectional study," *Journal of Public Health Research,* vol. 11, p. 22799036221139940, 2022.

[5] T. Dessie, "The Effect of Fee Waiver System on Health Service Utilization and Associated Factors Among Households in Farta District, Northwest Ethiopia, 2022," 2023.

[6] J. Jemal, T. Hagos, M. Fentie, and G. A. Zenebe, "Utilization of health services and associated factors among fee waiver beneficiaries in Dawunt district, North Wollo zone, Ethiopia," *Public Health,* vol. 205, pp. 110-115, 2022.

[7] H. Tesfaye, "Assessment of Utilization of Fee Waiver System among Beneficiaries in Addis Ababa, Ethiopia, 2017," Addis Ababa University, 2017.
