# Supplementary material for: Health service utilization and associated factors among fee waiver beneficiaries in Ethiopia: Systematic review and meta-analysis
Source: PLoS One. 2025 Jun 11;20(6):e0326131. doi: 10.1371/journal.pone.0326131 (PMC12157077; doi:10.1371/journal.pone.0326131)
Supplement: Attachment — Submitted filename: T_comments_PONE D-25-04792.docx [file pone.0326131.s006.docx]

Review Plos One article PONE-D-25-04792

Health service utilization and associated factors among fee waiver beneficiaries in

Ethiopia: Systematic review and meta-analysis

Abstract

- Conclusion 🡪 no need to put again the percentage. Re-paraphrase the conclusion as the findings. Line 41-42 can be leaved there as for the recommendation of the study.

Introduction: Perfectly clear

Method: okey

Results:

- Line 208 (OR = 0.69; 95% CI: 0.51, 0.95) 🡪 please check for the notation at the CI. I think it supposes to be minus (to) to replace coma (,).

Discussion: okey

Conclusion

- Line 285-286 can be deleted for this sentence “Based on seven primary studies with a total of 11,488 participants, the pooled prevalence of health service utilization was 60.57%, indicating moderate utilization levels” as it has already mentioned at the results and discussion.
