# Supplementary material for: Health service utilization and associated factors among fee waiver beneficiaries in Ethiopia: Systematic review and meta-analysis
Source: PLoS One. 2025 Jun 11;20(6):e0326131. doi: 10.1371/journal.pone.0326131 (PMC12157077; doi:10.1371/journal.pone.0326131)
Supplement: Attachment — Submitted filename: Response_to_reviewers_auresp_2.docx [file pone.0326131.s009.docx]

**Point by Point Response to the Editor, and Reviewers Comments**

Title: Health service utilization and associated factors among fee waiver beneficiaries in Ethiopia: Systematic review and meta-analysis

Manuscript ID: PONE-D-25-04792

Date: May, 2025

**Subject: Revision of the manuscript**

Dear Editor and Reviewers, we sincerely appreciate the time and effort you have dedicated to reviewing our manuscript and providing insightful comments. We found your feedback to be constructive and vital in improving the quality of our work. In response, we have carefully revised the manuscript and addressed all the questions and comments raised by the editors and reviewers. As requested, we have included a point-by-point response to your feedback below.

Thank you once again for your valuable input.

Sincerely,

On behalf of Co-authors.

Bizunesh Fantahun Kase (Corresponding Author)

**Journal Requirements**

1. Please ensure that your manuscript meets PLOS ONE's style requirements, including those for file naming. The PLOS ONE style templates can be found at https://journals.plos.org/plosone/s/file?id=wjVg/PLOSOne_formatting_sample_main_body.pdf and <https://journals.plos.org/plosone/s/file?id=ba62/PLOSOne_formatting_sample_title_authors_affiliations.pdf>

**Response:** Thank you for the reminder. We have reviewed and updated the manuscript and all associated files to ensure they meet PLOS ONE's style requirements.

2. As required by our policy on Data Availability, please ensure your manuscript or supplementary information includes the following:

A numbered table of all studies identified in the literature search, including those that were excluded from the analyses.

For every excluded study, the table should list the reason(s) for exclusion.

If any of the included studies are unpublished, include a link (URL) to the primary source or detailed information about how the content can be accessed.

A table of all data extracted from the primary research sources for the systematic review and/or meta-analysis. The table must include the following information for each study:

Name of data extractors and date of data extraction

Confirmation that the study was eligible to be included in the review.

All data extracted from each study for the reported systematic review and/or meta-analysis that would be needed to replicate your analyses.

If data or supporting information were obtained from another source (e.g. correspondence with the author of the original research article), please provide the source of data and dates on which the data/information were obtained by your research group.

If applicable for your analysis, a table showing the completed risk of bias and quality/certainty assessments for each study or outcome. Please ensure this is provided for each domain or parameter assessed. For example, if you used the Cochrane risk-of-bias tool for randomized trials, provide answers to each of the signalling questions for each study. If you used GRADE to assess certainty of evidence, provide judgements about each of the quality of evidence factor. This should be provided for each outcome.

An explanation of how missing data were handled.

This information can be included in the main text, supplementary information, or relevant data repository. Please note that providing these underlying data is a requirement for publication in this journal, and if these data are not provided your manuscript might be rejected.

**Response:** Thank you for your detailed and helpful instructions regarding data availability. We have ensured that all required information is included in the revised manuscript and supplementary materials.

3. We note you have included a table to which you do not refer in the text of your manuscript. Please ensure that you refer to Table 2 in your text; if accepted, production will need this reference to link the reader to the Table.

**Response:** Thank you for your helpful comment. We have now referenced Table 2 in the text of the manuscript.

4. Please include captions for your Supporting Information files at the end of your manuscript, and update any in-text citations to match accordingly. Please see our Supporting Information guidelines for more information: <http://journals.plos.org/plosone/s/supporting-information>.

**Response:** Thank you for your helpful suggestion. We have included captions for all Supporting Information files at the end of the revised manuscript.

5. Please review your reference list to ensure that it is complete and correct. If you have cited papers that have been retracted, please include the rationale for doing so in the manuscript text, or remove these references and replace them with relevant current references. Any changes to the reference list should be mentioned in the rebuttal letter that accompanies your revised manuscript. If you need to cite a retracted article, indicate the article’s retracted status in the References list and also include a citation and full reference for the retraction notice.

**Response:** Thank you for your careful review. We have thoroughly reviewed the reference list and can confirm that no retracted references are included in the manuscript. All references cited are current and appropriate for the study.

**Editor's Comments and Authors’ Response**

1. The finding on residence is missing from the abstract results.

**Response:** We appreciate your careful review of our manuscript. In response to your comment, we have now included the finding on residence in the abstract results section.

2. The introduction can be improved by consolidating the first two paragraphs into one and highlighting fee waiver as a pathway to universal health coverage and equitable access to health care.

**Response:** Thank you for your valuable comment. We have addressed this point in the revised manuscript as requested.

3. Lines 50-51 focus on rural and remote areas. The authors should also highlight urban inequities in access to healthcare.

**Response:** We appreciate the suggestion to address urban inequities in access to healthcare. In response, we have revised the manuscript to highlight urban inequalities alongside those in rural areas.

4. In line 67-71, the authors describe the fee waiver policy in Ethiopia, but this case description needs expansion.

**Response:** Thank you for your valuable comment. We have now expanded the description to provide greater clarity and context.

5. In line 72-75, the authors analyse the knowledge gap, but this gap analysis is not comprehensive. The authors should review the pertinent literature on how fee waiver policies influence health service utilization elsewhere to enrich the gap analysis.

**Response:** Thank you for your insightful suggestion. We have addressed this comment by expanding the gap analysis in the revised manuscript. We have reviewed and incorporated relevant literature on the influence of fee waiver policies on health service utilization in other settings.

6. The authors should also be more systematic in the Discussion section. Discuss the pooled prevalence, regional and sub-group variations first, and then the factors affecting health service utilisation.

**Response:** Thank you for your constructive feedback. We have considered your suggestion and revised the Discussion section accordingly.

7. Notably, the findings about place of residence was not discussed. So, the authors should include a discussion of the urba--n-rural disparity in health service utilisation.

**Response:** Thank you for highlighting this important point. We have addressed your comment by including a discussion of the urban-rural disparity in health service utilization in the revised manuscript.

8. In the Background, line 57-61 of the revised manuscript focused on the pertinent literature. However, four papers are few, and do not represent the existing scholarship in this area. Equally, the ommitted the review of the factors associated with utilization of free care services.

**Response:** Thank you for the comment. We have expanded the Background section to include additional relevant literature and add information on key factors associated with the utilization of health services among fee waiver beneficiaries as suggested**.**

9. In the Discussion section, the authors should expunge the last sentence of the first paragraph of the Discussion section. In its place, they should summarize the key findings that were subsequently discussed.

**Response:** Thank you. We have removed the sentence and replaced it with a summary of the key findings as advised.

10. In the Discussion, the authors completely ommitted comparing the study's findings with existing literature. It is the practice to compare study findings to existing literature and explain any variations. Doing this will enrich the discussion.

**Response:** Thank you for the insightful comment. We have now expanded the Discussion section to compare our findings with existing literature.

**Reviewer #1**

1. The manuscript is well-structured, and the data presented robustly supports the conclusions drawn. All data is fully accessible, and the statistical analysis has been conducted with appropriate rigor. While the manuscript is generally clear and written in standard English, several grammatical errors are present throughout. These should be carefully reviewed and revised to ensure the precise and accurate communication of the research findings, thereby enhancing clarity and reinforcing the author's expertise.

**Response:** Thank you for your positive feedback. We have carefully reviewed the manuscript and revised the grammatical errors to ensure accurate communication of our findings.

**Reviewer #2**

1. Abstract: no need to put again the percentage at the conclusion. Re-paraphrase the conclusion as the findings.

**Response:** We appreciate your suggestion. We have revised the conclusion section of the abstract by removing the repeated percentage and rephrasing it.

2. Line 41-42 can leave there as for the recommendation of the study.

**Response:** Thank you for your valuable comment. We included the statement in the abstract in accordance with the PLOS ONE submission guidelines, which recommend summarizing the most important results and their significance. Additionally, previously published research articles in the journal also report recommendations under the conclusion section of the abstract. However, if our explanation does not fully address your concern, we are willing to revise the manuscript based on your recommendation.

3. Introduction: perfect and clear

**Response:** Thank you for the positive feedback on the introduction.

4. Methods: okey

**Response:** We appreciate your positive assessment.

5. Results: Line 208 (OR = 0.69; 95% CI: 0.51, 0.95), please check for the notation at the CI. I think it supposes to be minus (-) to replace coma (,).

**Response:** Thank you for your comment. We have reviewed and corrected the notation in the confidence interval.

6. Discussion: Okey

**Response:** Thank you for the positive feedback

7. Conclusion: Line 285-286 can be deleted for this sentence “Based on seven primary studies with a total of 11,488 participants, the pooled prevalence of health service utilization was 60.57%, indicating moderate utilization levels” as it has already mentioned at the results and discussion.

**Response:** Thank you for your insightful comment. We agree with your suggestion and have deleted the sentence to avoid redundancy.
